# Target Design of Novel Histone Deacetylase 6 Selective Inhibitors with 2-Mercaptoquinazolinone as the Cap Moiety

**DOI:** 10.3390/molecules27072204

**Published:** 2022-03-28

**Authors:** Hue Thi Buu Bui, Phuong Hong Nguyen, Quan Minh Pham, Hoa Phuong Tran, De Quang Tran, Hosun Jung, Quang Vinh Hong, Quoc Cuong Nguyen, Quy Phu Nguyen, Hieu Trong Le, Su-Geun Yang

**Affiliations:** 1 Department of Chemistry, College of Natural Sciences, Can Tho University, Can Tho 900000, Vietnam; tqde@ctu.edu.vn (D.Q.T.); quangb1705294@student.ctu.edu.vn (Q.V.H.); quocb1703737@student.ctu.edu.vn (Q.C.N.); phuquy080@gmail.com (Q.P.N.); hieule1509@gmail.com (H.T.L.); 2 Department of Biomedical Science, BK21 FOUR Program in Biomedical Science and Engineering, Inha University College of Medicine, Incheon 22212, Korea; phuong.hn.jcb@gmail.com (P.H.N.); phuonghoa1404@gmail.com (H.P.T.); wjdghtjs1234@naver.com (H.J.); 3 Inha Institute of Aerospace Medicine, Inha University College of Medicine, Incheon 22332, Korea; 4 Institute of Natural Products Chemistry, Vietnam Academy of Science and Technology, Ha Noi 100000, Vietnam; minhquanaries@gmail.com; 5 Faculty of Chemistry; Vietnam Academy of Science and Technology, Graduate University of Science and Technology, Ha Noi 100000, Vietnam

**Keywords:** hydroxamic acid-based histone deacetylase inhibitors, 2-mercaptoquinazolinone, anticancer drugs, antiproliferative activity, tubulin acetylation level

## Abstract

Epigenetic alterations found in all human cancers are promising targets for anticancer therapy. In this sense, histone deacetylase inhibitors (HDACIs) are interesting anticancer agents that play an important role in the epigenetic regulation of cancer cells. Here, we report 15 novel hydroxamic acid-based histone deacetylase inhibitors with quinazolinone core structures. Five compounds exhibited antiproliferative activity with IC_50_ values of 3.4–37.8 µM. Compound **8** with a 2-mercaptoquinazolinone cap moiety displayed the highest antiproliferative efficacy against MCF-7 cells. For the HDAC6 target selectivity study, compound **8** displayed an IC_50_ value of 2.3 µM, which is 29.3 times higher than those of HDAC3, HDAC4, HDAC8, and HDAC11. Western blot assay proved that compound **8** strongly inhibited tubulin acetylation, a substrate of HDAC6. Compound **8** also displayed stronger inhibition activity against HDAC11 than the control drug Belinostat. The inhibitory mechanism of action of compound **8** on HDAC enzymes was then explored using molecular docking study. The data revealed a high binding affinity (−7.92 kcal/mol) of compound **8** toward HDAC6. In addition, dock pose analysis also proved that compound **8** might serve as a potent inhibitor of HDAC11.

## 1. Introduction

Histone deacetylases (HDACs) are involved in the epigenetic regulation of gene transcription by removing the acetyl groups from histones. Due to the robust power of target gene regulation, HDACs are closely related with various cellular biological events and have been investigated for potential drug targets of various diseases, such as cancer, cardiac events, degenerative nerve diseases and other inflammation-related diseases [1].

The HDACs are divided into 18 subtypes that are categorized into four groups, namely, class I with subclasses Ia (HDAC1 and 2), Ib (HDAC3), and Ic (HDAC8); class II with subclasses IIa (HDACs 4, 5, 7, and 9) and IIb (HDACs 6 and 10); class III (Sirt1-7); and class IV (HDAC11) [2,3]. Classes I, II and IV are zinc-dependent amidohydrolases and class III consists of NAD+-dependent HDAC homologous to the yeast Sir2 protein (Sir2-like or sirtuins: Sirt1-7). Each subtype of HDAC has been proved to be directly associated with cancer and other related diseases. Therefore, specific targeting design of HDAC inhibitors (HDACIs) is very critical to achieve the intended therapeutic effects.

**Figure 1:**
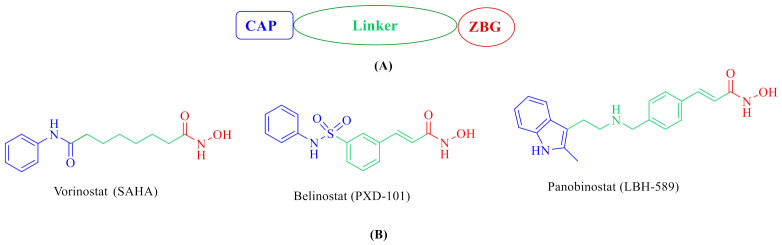
(**A**) Basic pharmacophore features of HDACIs; (**B**) FDA approved HDACIs.

In general, HDACI consists of three functional moieties: a lipophilic and surface recognition cap group, which interacts with residues of the active site, a linker that occupies the narrow channel of active site of HDACs, and a zinc binding group (ZBG) interacting with the zinc ion at the active site (Figure 1A) [4]. HDACIs can be structurally categorized into principal structural classes: hydroxamic acids, small molecular weight carboxylates, benzamides, and cyclic peptides. However, natural HDACIs also exist, belonging to bioflavonoids and called flavones, structurally different from known HDACIs [5,6]. Among HDACIs, hydroxamate-based organic molecules are the most potent due to their strong metal ion binding property [7]. Various hydroxamic acid-containing molecules have been synthesized and evaluated for their activities [8]. Vorinostat (SAHA) [9], Panobinostat (LBH-589) [10], and Belinostat (PXD-101) [11] are all hydroxamic acid-based HDACIs which have been approved by the US FDA for the treatment of refractory cutaneous/peripheral T-cell lymphoma (Figure 1B). However, most hydroxamates are pan-HDACIs that act on all HDAC classes with several obvious side effects including fatigue, thrombo-cytopenia and gastrointestinal issues [12,13], and isoform selective HDACIs may possess a better therapeutic index and fewer adverse effects [14,15,16].

**Figure 2:**
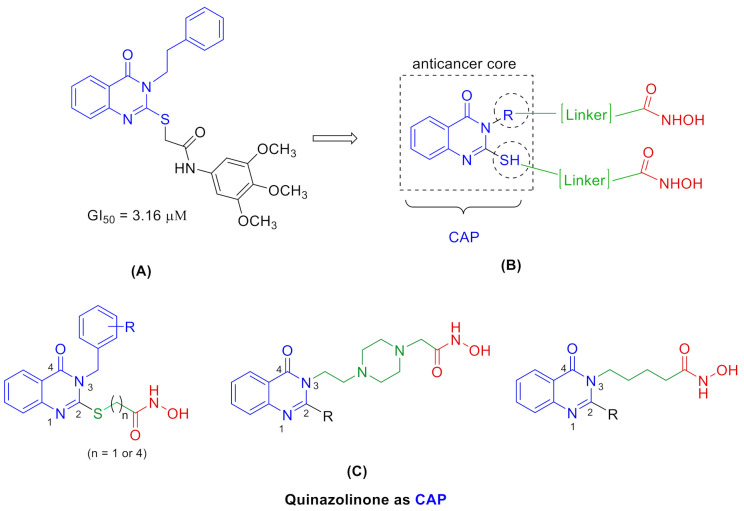
(**A**) Structure of 2-mercaptoquinazoline as the antitumour activity; (**B**) rational design template for quinazolinone hydroxamate conjugates; and (**C**) general structures of the target compounds.

Among the HDACs, HDAC6 regulates microtubule-dependent cell movement via deacetylation of non-histones, such as tubulin, Hsp90 and cortactin [17]. Over-expression of HDAC6 is closely linked to the invasive metastatic properties of cancer cells [18]. Genetic ablation, induced by HDAC1-3, had a lethal effect, whereas HDAC6 knockout mice showed a normal phenotype. HDAC6 inhibitors do not seem to be cytotoxic toward mammalian cells and may have fewer side effects than pan-HDAC inhibitors and HDAC1–3 selective inhibitors [19,20,21]. Thus, target-specific design of HDAC6 inhibitors appears to be more appropriate than design of inhibitors targeting other isoforms. To date, many hydroxamate-based HDAC6 inhibitors with modified the cap structural entity have been reported, as this pharmacophore is responsible for the HDAC isoform selectivity by mediating interactions with the amino acid residues at the active site of the enzyme [22,23,24]. Additionally, HDACI with quinazolinones cap moiety has attracted the great interest of many medicinal chemists because of its strong anticancer activity [25,26,27]. Adel et al. have recently reported that 2-mercaptoquinazoline analogues (Figure 2A) showed broad spectrum antitumor activity toward several tumor cell lines and proved to be 10-fold more active than 5-FU, with half maximal growth inhibition concentration (GI_50_) values of 2.2 and 2.4 µM, respectively [28].

To the best of our knowledge, there have been no reports in the literature using the 2-mercaptoquinazoline as a cap for HDAC6 selective inhibitors. In continuation with our anticancer drug development studies [29,30,31], herein, we extended our approach with quinazolinone-conjugated hydroxamic acid, which leads to potent and selective HDAC6 inhibitors. Our design used a quinazolinone core as a cap with the hydroxamic acid side chains at C-2 or N-3 position. This design, manipulating the surface recognition cap group and linker region while retaining the hydroxamic acid as ZBG, is expected to generate a novel hybrid structure that improves HDAC6 inhibitory activity.

In this study, a series of 15 novel HDACIs with 2-mercaptoquinazolinone cap moiety were synthesized and evaluated. For rationalization of the selectivity of novel HDACIs for HDAC6, we performed a molecular docking study with ligand binding free energy estimation and in vitro HDAC isoform inhibition assay. Target effect of HDACIs was confirmed through Western blot analysis of acetylated tubulin (HDAC6 substrate) in MCF-7 human breast cancer cells.

## 2. Results and Discussion

### 2.1. Structural Design and Synthesis

To search the structure and activity relationship analysis (SAR) of the cap and the linker, we designed two series of HDAC inhibitors which used quinazolinone analogues as capping groups and different types of linkers (Figure 2B). As illustrated in Figure 2C, the general structures of the target compounds include the capping quinazolinone moieties which are linked to the hydroxamate-based ZBG group through a one or four carbon thioether linkage at position C-2 or through an alkyl or 3-piperazinylethyl at position N-3.

**Scheme 1:**
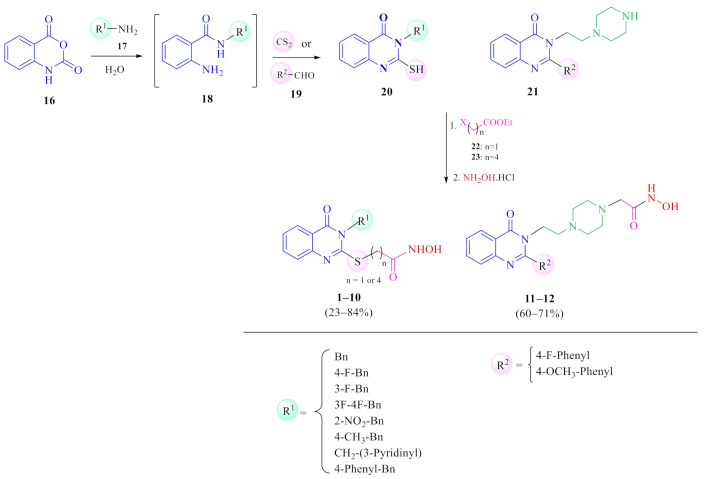
Synthesis of quinazolinone-based hydroxamate derivatives (**1–12**). (Chemical structures of the compounds were prepared by using the Chemdraw Professional 2016 Program Version 16.0.1.4.)

The synthesis of the target quinazolinone bearing the hydroxamic acid at the C-2 position through a thioether linkage (**1–10**) and at the N-3 position through the 3-piperazinylethyl linkage (**11–12**) is presented in Scheme 1. Starting from isatoic anhydride (**16**), ring opening occurred by appropriate substituted benzylamines or 2-(piperazin-1-yl)ethan-1-amine in water at room temperature to provide the corresponding *N*-substituted 2-aminobenzamides **18**, which were then condensed with either carbon disulfide or aromatic aldehyde **19** to afford 2-mercaptoquinazolinones **20** or 3-piperazinylethyl quinazolinone **21**, respectively (Scheme 1) [29,32,33,34]. The thiol moiety of **20** was then coupled with ethyl 2-chloroacetate **22** or ethyl 5-bromopentanoate **23** via nucleophilic substitution mechanism under basic conditions (K_2_CO_3_) to incorporate the ester function which then underwent aminolysis with hydroxylamine to afford the desired hybrid thioether linked hydroxamate quinazolinones **1–10** in moderate to good yields over three steps (13–69%, Table 1) [35]. The same reaction sequence was applied for **21** with ethyl 2-chloroacetate **22** to form quinazolinone-based hydroxamates **11–12** in good overall yields (30–40%).

**Scheme 2:**
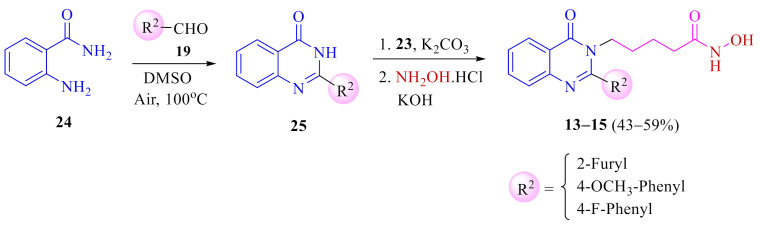
Synthesis of quinazolinone-based hydroxamate derivatives (**13–15**).

Scheme 2 shows the preparation of quinazolinone-based hydroxamate derivatives in which the 3-piperazinylethyl group at the N-3 position of compounds **11–12** is replaced with a chain linker having five carbons. The target compounds (**13–15**) were conveniently obtained via an aerobic oxidative cyclization of anthranilamide (**24**) with appropriate aromatic aldehyde **19** in wet DMSO to provide 2-substituted quinazolinones (**25**) [36]. Subsequent reactions were conducted as described in Scheme 1 and accomplished the desired hydroxamates (**13–15**) in rather good overall yields (30–53%, three steps) (Table 1).

### 2.2. Biological Evaluation

#### 2.2.1. Inhibition Screening

The MCF-7 cell line was obtained from Korean Cell Line Bank (KCLB, Seoul, Korea). The cells were cultured in vitro in DMEM (Invitrogen, Paisley, UK) containing 10% fetal bovine serum (Gibco, Thermo Fisher Scientific, Waltham, MA, USA) and 1% penicillin. Cells were treated with different concentrations of compounds (**1–15**) in a 1:3 the highest concentration was 250 µM (before dilution) and further incubated for 48 hrs. The screening results are summarized in Table 1. As a result, four compounds (**9**, **10**, **13** and **14**) showed moderate cytotoxicity with IC_50_ ranging from 14.2~37.8 µM. Remarkably, compound **8** exhibited strong activity with an IC_50_ value of 3.4 µM, comparable to the positive control Belinostat (IC_50_ = 2.6 µM). We evaluated the antiproliferative activity of compound 8 against a variety of cells. Compound **8** showed inhibitory effects against different cell lines (Supporting Information S2).

Based on the antiproliferative activities of the compounds (Table 1), structure activity relationships (SAR) were suggested. All compounds with one carbon length alkyl chain as the linker (compounds **1–6**) as well as quinazolinones bearing a 3-piperazinylethyl linker (compounds **11–12**) or saturated four carbon linker (compounds **13–15**) were generally inactive. In the case of quinazolinone-based hydroxamates with four carbon length alkyl chain as a linker, the presence of the biphenyl group on the benzene ring at the N-3 position (compound **10**) resulted in moderate activity (IC_50_ = 14.2 µM). Notably, substitution of the phenyl group of **10** by fluorine (compound **8**) led to remarkable increase in activity (4-fold decrease in IC_50_ value) which was comparable to the positive control Belinostat. In contrast, the introduction of an additional fluorine group (compound **9**) only led to the loss of activity. Based on these findings, the hydroxamate **8** bearing 2-mercaptoquinazolinone as the cap pharmacophore, a halogen (F) group on the benzene ring at the N-3 position of the heterocycle, and a four-carbon length alkyl chain as a linker was defined as a lead compound.

#### 2.2.2. In Vitro HDAC Isoform Inhibition Activity of Compound **8**

**Figure 3:**
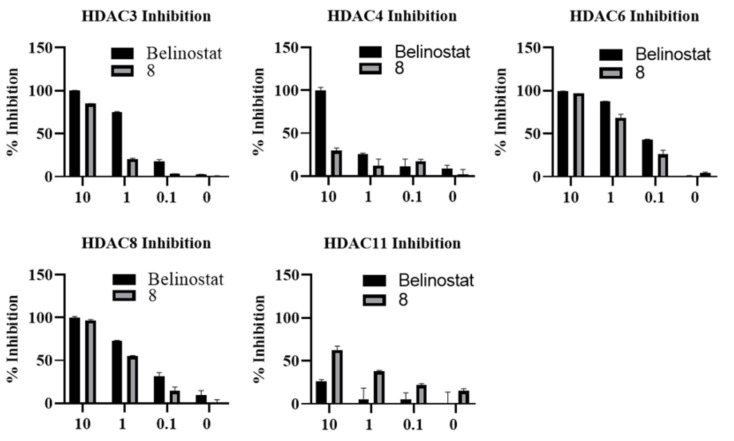
HDAC inhibition activity of compound **8**. Representatives DRCs (dose–response curves) of **8** (closed bars) and reference Belinostat (open bars). Percentage inhibition is shown in each graph.

Compound **8** was introduced to the inhibition study against the HDAC3, HDAC4, HDAC6, HDAC8, and HDAC11 isoforms, and Belinostat was used as a control (Figure 3, Table 2). As shown in Figure 3, compound **8** showed inhibitory activity against all HDACs in a dose-dependent manner. Remarkably, compound **8** with a 2-mercaptoquinazolinone cap moiety exhibited the highest selectivity for HDAC6 (IC_50_ value of 0.3 µM) compared to class I HDAC3 (7.9-fold) and HDAC8 (2.3-fold), class IIa HDAC4 (>29.4-fold), and class IV HDAC11 (>29.4-fold). These results indicated that compound **8** is the most potent and selective HDAC6 inhibitor. Interestingly, compound **8** showed higher activity against HDAC11 compared to the reference in a dose-dependent manner.

#### 2.2.3. Selective Upregulation in the Acetylation Level of Tubulin

**Figure 4:**
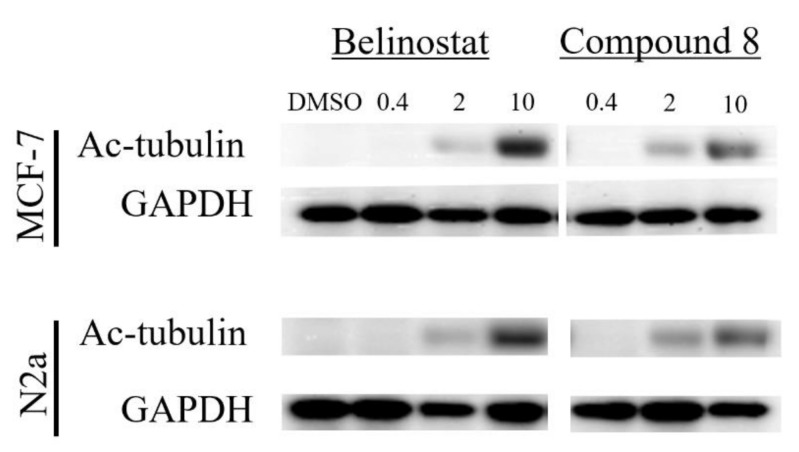
Western blot analysis of acetylated tubulin (HDAC6 substrate) in MCF-7 and N2a after 6 h of treatment with compound **8** and Belinostat at 10, 2, and 0.2 µM. GAPDH was used as a loading control.

The effect of compound **8** and Belinostat on the acetylation of tubulin (substrate for HDAC6), a biomarker of HDAC inhibition, in MCF7 and N2a cells was measured by Western blotting (Figure 4). As shown in Figure 4, compound **8** induced acetylated tubulin levels in a dose-dependent manner.

### 2.3. Molecular Docking Studies

**Figure 5:**
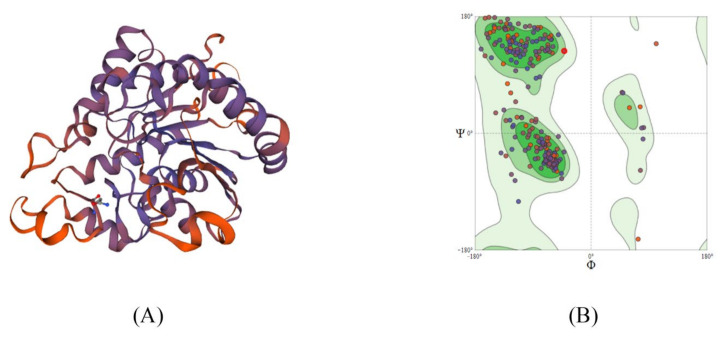
(**A**) Swiss-Model of HDAC11; (**B**) Ramachandran plot analysis of the structure of HDAC11 model (Figures were prepared using Chimera 1.14 and SWISS–MODEL webserver).

The three-dimensional structure of HDAC11 was built by homology modeling on the Swiss-Model webserver (Figure 5A). In general, a good quality model is defined as more than 90% of the amino acids located in the most favored regions [37]. The obtained data from the Ramachandran plot showed that 91.69% amino acids of the HDAC11 model are located in the most favored regions (Figure 5B), thus, our model was considered as a liable model for further docking studies.

AutoDock4 is one of the most popular docking software that has been widely used with more than 6000 citations since 2010. This is a useful tool to rapidly predict the binding affinity of ligands toward a specific protein/enzyme target [38,39,40]. Given the rapid increase in the number of metalloprotein studies recently, AutoDock4Zn has been developed with improvements in the AutoDock force field for small-molecule docking to zinc metalloproteins [41]. As HDACs are well-known metalloproteins with zinc ions included within the active site, AutoDock4Zn was selected to study the binding mode of the test compounds with these enzymes.

**Figure 6:**
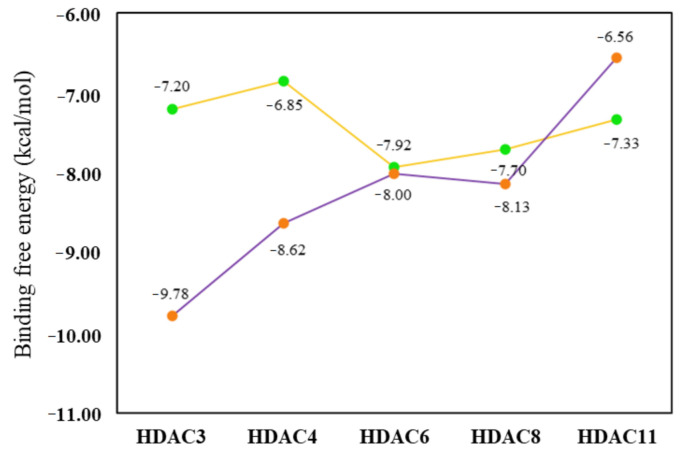
Plot of ligand binding free energy between compound **8** (yellow line and green dot) and Belinostat (purple line and orange dot) toward studied HDAC enzymes.

Table 3 presents the docking results of the studied compounds with HDAC enzymes. According to the ranking criteria of Autodock4Zn, the more negative docking energy suggests the higher binding affinity of the compound towards the targeted receptor [38,42]. According to the obtained data, both compound **8** and Belinostat were observed to create common hydrogen bonds with essential residues within the active site of HDAC3 (His172) and HDAC8 (Cys153, Asp178). Notably, other studies have reported that HDACIs, which form interactions with residues His142, Cys153, Asp178, His180, and Phe208, can lead to enzyme malfunction [43,44]. In this study, the binding free energies of compound **8** towards the target enzymes were −7.20 kcal/mol for HDAC3 and −7.70 kcal/mol for HDAC8, whereas the values of Belinostat were −9.78 and −8.13 kcal/mol, respectively (Figure 6). These data coincide with the IC_50_ values obtained from the in vitro HDAC inhibition assay; while Belinostat exhibits high binding affinity toward HDAC3 and HDAC8, its IC_50_ values on these enzymes are lower than those of compound **8**.

**Figure 7:**
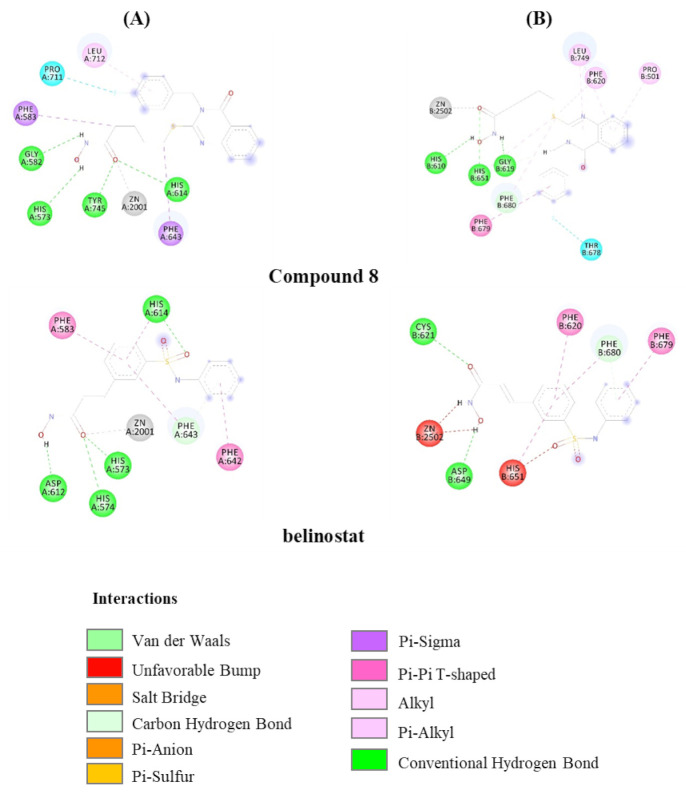
Docking conformation of compound **8** and Belinostat with HDAC enzymes. (**A**) Docking pose of studied ligand with HDAC6; (**B**) docking pose of studied ligand with HDAC11. (Docking images were prepared by using the Discovery Studio Vizualizer version 2021).

Binding orientation analysis of the studied ligands with HDAC4 revealed that Belinostat docked to this enzyme at higher binding affinity than compound **8** (−8.62 kcal/mol and −6.85 kcal/mol, respectively) (Table 3). In addition, dock pose analysis revealed that the amino acid residues involved in forming H-bond interactions with compound **8** were Leu943, Gly975, and His976. It is notable that these residues did not participate in constituting the active site of HDAC4, thus the interaction formed between compound **8** and HDAC4 residues might not lead to enzyme malfunction. These results suggest that compound **8** might not be the potential inhibitor of HDAC4 (Figure 7).

Regarding enzyme HDAC6, the obtained results showed that compound **8** and the reference ligand docked to this enzyme with an approximate equivalent dock score (−7.92 and −8.00 kcal/mol, respectively). Amongst the formed interactions, compound **8** and the reference ligand created the same H-bonding towards residues His573 and His614 within the active site of HDAC6, thus suggesting these compounds might inhibit the function of this enzyme in the same mechanism of action (Figure 7). In addition, the in vitro inhibition data were in correlation with the docking results since the IC_50_ value of compound **8** was recorded at 0.3 μM and 0.2 μM for Belinostat.

Dock score analysis of the studied compounds with HDAC11 showed interesting results when compound **8** proved itself as the best candidate to interact with this enzyme with high binding affinity (−7.33 kcal/mol) in comparison to Belinostat (−6.56 kcal/mol). Docking conformation results indicated that while Belinostat formed hydrogen bonds with Cys621 and Asp649, compound **8** created three hydrogen bonds with other residues including His610, Gly619 and His651. In addition, the interaction between the carbonyl group with the zinc ion within the active site of HDAC11 was not observed in the case of Belinostat. The obtained data might suggest an explanation for the observed low IC_50_ value and selective inhibitory activity of compound **8** (about 3.0 μM) towards the targeted enzyme as compared to the control Belinostat.

In summary, the designed compound **8** was found to selectively target HDAC6 over HDAC3, HDAC4, HDAC8, and HDAC11, and strongly inhibited tubulin acetylation. In addition, compound **8** also displayed stronger inhibition activity against HDAC11 than the drug Belinostat. These findings proved that the novel compound **8** might serve as a potent HDAC6 selective inhibitor as well as a potent inhibitor of HDAC11. However, we should note that linear hydroxamic acids linked to quinazolin-4(3*H*)-one moieties at different positions via different types of linkages have inhibitory activity against bromo-domain-containing protein 4 (BRD4), matrix metalloproteinases (MMPs) and HDACs [25]. Additionally, studies are ongoing in our lab for more characterization of compound **8** with specific organs, such as lung and breast cancer, or whether it exerts other non-HDAC inhibitory activity which will be reported in due course.

## 3. Conclusions

To develop novel HDAC6 selective inhibitors with a quinazolinone cap group, a series of hydroxamic acid analogues was prepared and evaluated for their bioactivities. Compound **8**, with a cap of 2-mercaptoquinazolinone, showed the highest anticancer effect on cells. The antiproliferation assay revealed the potency of compound **8** against seven diverse cancer cell lines. The inhibitory activities of the synthesized compound on HDAC isoforms (3, 4, 6, 8, and 11) and SAR analysis confirmed that the strategy successfully achieved the goal of finding a novel selective and highly efficient small-molecule inhibitor of HDAC6. Compound **8** displayed the highest potency (at least eight-fold higher selectivity) for HDAC6 over other isoforms with an IC_50_ value of 0.3 µM. Western blot analysis further confirmed the increased acetylation level of tubulin at 10 µM concentration. Molecular docking studies indicated that compound **8** had the highest binding affinity toward HDAC6 among the investigated HDAC enzymes (−7.92 kcal/mol), which was close to the dock score of the reference ligand (Belinostat). Dock poses analysis further suggested that compound **8** inhibited HDAC6 through the H-bond interactions with key residues His573, Gly582, His614 and Tyr745 within the active site of the enzyme. Thus, compound **8** could serve as a promising candidate to inhibit HDAC6 enzyme activity. On the other hand, the obtained data also revealed that compound **8** might be a selective inhibitor of HDAC11.

## 4. Experimental Section

### 4.1. Chemistry

#### General Information

Reactions were monitored by thin-layer chromatography (TLC) on 0.2 mm pre-coated silica-gel 60 F254 plates (Merck). ^1^H nuclear magnetic resonance (NMR) and ^13^C NMR spectra were measured with Bruker Avance 300 MHz, Bruker Avance 500 MHz, and Bruker Avance 600 MHz spectrometers. Mass spectrometry (MS) data were recorded on an 1100 series LC-MSD-Trap-LS Agilent spectrometer, and high-resolution electrospray ionization mass spectrometry (HRESI-MS) observations were performed on a Bruker MicrOTOF-Q mass spectrometer. Fourier transform infrared (FT-IR) spectroscopy was conducted as per the KBr pellet method on a Thermo Nicolet 6700 spectrometer. Chemical shifts are shown in parts per million (ppm) relative to tetramethyl silane (Me_4_Si, *δ* = 0); *J* values are given in Hertz.

### 4.2. Molecular Docking Studies

#### 4.2.1. Ligand and Receptor Preparation

The three-dimensional structures of compounds were prepared using MarvinSketch version 19.27.0 and PyMOL version 1.3r1 [45]. Energy minimization of the studied ligands was conducted using MM2 force field and quantum chemical calculations were performed at the B3LYP/6-31g(d,p) level implemented in Gaussian 09 [46]. Belinostat, a well-known inhibitor of histone deacetylase, was selected as reference ligand [47].

The protein models chosen for docking studies were obtained from Protein Data Bank archive with PDB IDs as follows: 4A69 (HDAC3), 4CBT (HDAC4) [15], 5EEI (HDAC6), 1T67 (HDAC8) [43,48,49,50]. The amino acid sequence of HDAC11 is publicly available at NCBI, National Center for Biotechnology Information, archived under entry ID: NP_079103.2. The Swiss-Model webserver (http://swissmodel.expasy.org, accessed on 15 December 2021) was utilized for the prediction of HDAC11 model. The Graphical User Interface program named Autodock Tools 1.5.6rc3 (ADT) was employed to set up input data. Details of molecular docking simulation are given in Supplementary Materials.

#### 4.2.2. Docking Using AutoDock4Zn

The binding site was enclosed in a grid box with zinc ion in the center. Search space was defined in x, y and z dimensions (64 × 64 × 64) and the spacing between grid points was 0.375 Å. Grid input file also included the parameter file AD4Zn.dat, which is a specific parameter file for zinc ion. Initially, AutoGrid was run to generate the grid map of various atoms of the ligands and receptors. Upon completion of AutoGrid, molecular docking study was performed utilizing AutoDock4Zn with Lamarckian genetic algorithm (LGA) for searching the optimum dock pose together with scoring function to calculate the binding affinity [42]. A total of 50 runs were performed for each docking and the rest of the parameters were set to default values.

### 4.3. Biological Methods

#### 4.3.1. Antiproliferative Assay

The MCF-7 cell line was obtained from ATCC. The cells were cultured in vitro in DMEM (Invitrogen, Paisley, UK) containing 10% fetal bovine serum (Gibco, Thermo Fisher Scientific, Waltham, MA) and 1% penicillin. For cytotoxicity assays, MCF-7 cells were seeded into 96-well culture plates at 3 × 10^5^ cells/well in 0.1 mL of DMEM and incubated at 37 °C with 5% CO_2_ and 90% humidity. After 16 h, the cells were treated with drugs (250 μM, serially diluted up to three-fold) for 48 h. Cell viability was measured using the CELLOMAXTM Viability Assay kit (Precaregene Co., Seoul, Korea). UV absorption at 450 nm wavelength was measured using an Infinite M200 microplate reader (Tecan, Zürich, Switzerland). The IC_50_ values were calculated using Prism v7.0 (GraphPad Software Inc., La Jolla, CA). The inhibitory activity of the tested compounds was evaluated by an 8-point dose–response curve (DRC) analysis in triplicates.

The antiproliferative activities of compound **8** against seven solid tumor cell lines, human ovarian cancer SKOV-3 cells, breast cancer MCF-7, MDA-MB-231, and EMT6 cell lines, liver cancer Hepa1c1c7 cells, lung cancer A549 cells, pancreatic cancer PANC1 cells, and brain N2a cells were evaluated using the CELLOMAXTM Viability Assay kit, and Belinostat was used as positive control.

#### 4.3.2. HDAC Enzyme Inhibition Assays

The compounds were diluted to the indicated concentrations and mixed with HDAC enzymes (HDAC1, HDAC3, HDAC6, HDAC8, HDAC11) and the substrate for 1 h (PBS Biosciences). The fluorescence intensity was measured according to the manufacturer’s instructions. The inhibition rates of the test compounds were calculated based on dimethyl sulfoxide (vehicle)-treated group and positive control (Belinostat).

#### 4.3.3. Western Blotting

MCF-7 and N2a cell lines were incubated with various concentrations of test agents for 6 h. Cell lysis and Western blot analyses were performed as previously described. Anti-acetylated tubulin antibody and horseradish peroxidase (HRP)-conjugated secondary antibodies (Santa Cruz Biotechnology) were used. Immunoreactive bands were visualized using enhanced chemiluminescence (Millipore, Burlington, MA, USA).

## Figures and Tables

**Table 1:** Synthesis and antiproliferative activities of hydroxamate derivatives (**1–15**) against MCF-7 cell lines.

Compounds	R^1^	R^2^	n	Yield (%)	IC_50_ (µM)	Imax (%)
**1**	Bn	-	1	32	ND	20.0
**2**	4-F-Bn	-	1	13	ND	11.0
**3**	3-F-Bn	-	1	15	ND	24.0
**4**	3F-4F-Bn	-	1	44	ND	23.0
**5**	2-NO_2_-Bn	-	1	20	ND	17.0
**6**	4-CH_3_-Bn	-	1	69	ND	22.0
**7**	CH_2_-(3-pyridinyl)	-	4	23	ND	17.0
**8**	4-F-Bn	-	4	44	3.44 ± 1.11	90.6
**9**	3F-4F-Bn	-	4	51	36.9 ± 1.12	96.8
**10**	4-phenyl-Bn	-	4	25	14.2 ± 1.14	89.7
**11**	(CH_2_)_2_-piperazinyl	4-OCH_3_-Phenyl	-	30	ND	3.0
**12**	(CH_2_)_2_-piperazinyl	4-F-Phenyl	-	40	ND	3.0
**13**	-	4-OCH_3_-Phenyl	-	46	37.26 ± 1.21	95.8
**14**	-	4-F-Phenyl	-	53	37.85 ± 1.71	100
**15**	-	2-Furyl	-	30	ND	1.5
Belinostat	-	-	-	-	2.6 ± 1.40	100

Bn: Benzyl; ND: not determined.

**Table 2:** HDAC inhibitory activities of compound **8**.

	IC50 (µM)					Selective Fold (Ratio)			
	HDAC3 (Class I)	HDAC8 (Class I)	HDAC4 (Class IIa)	HDAC6 (Class IIb)	HDAC11 (Class IV)	HDAC3/ HDAC6	HDAC4/ HDAC6	HDAC8/ HDAC6	HDAC11/ HDAC6
**8**	2.7 ± 0.08	0.8 ± 0.01	>10	0.3 ± 0.02	>3	7.94	>29.4	2.32	>29.4
Belinostat	0.6 ± 0.09	0.3 ± 0.02	2.7 ± 0.12	0.2 ± 0.01	>10	3.0	13.5	1.5	>50

**Table 3:** Docking results of studied compounds and hydrogen bond interacting residues.

Enzyme	Dock Score (kcal/mol)			H-Bond Interacting Residues	
	Compound 8	Belinostat		Compound 8	Belinostat
			Class I		
HDAC3	−7.20	−9.78		His172, Asp259, Tyr298	Gly143, Cys145, His172
HDAC8	−7.70	−8.13		Lys33, Cys153, Asp178, Phe208	Gly140, Cys153, Asp178, Gln263
			Class IIa		
HDAC4	−6.85	−8.62		Leu943, Gly975, His976	His802, His803, Phe871, Asp934, Pro942
			Class IIb		
HDAC6	−7.92	−8.00		His573, Gly582, His614, Tyr745	His573, His574, Asp612, His614
			Class IV		
HDAC11	−7.33	−6.56		His610, Gly619, His651	Cys621, Asp649

## Data Availability

Not applicable.

## Supplementary Materials

The following supporting information can be downloaded at: https://www.mdpi.com/article/10.3390/molecules27072204/s1, Scheme S1. Synthesis of quinazolinone based hydroxamates (**1–10**); Scheme S2. Synthesis of quinazolinone based hydroxamates (**11–12**); Scheme S3. Synthesis of quinazolinone based hydroxamates (**13–15**); Table S1. Antipreliferation of compounds on different cell lines; Figure S4.1–S4.75. Scanned NMR spectra of compounds (**1–15**).
