# Supplementary material for: Target Design of Novel Histone Deacetylase 6 Selective Inhibitors with 2-Mercaptoquinazolinone as the Cap Moiety
Source: Molecules. 2022 Mar 28;27(7):2204. doi: 10.3390/molecules27072204 (PMC9000625; doi:10.3390/molecules27072204)
Supplement: Supplementary file 1 [file molecules-27-02204-s001.zip › molecules-1618904-supplementary.pdf]

SUPPORTING INFORMATION

Discovery of Selective Histone Deacetylase 6 Inhibitor Using the 2-Mercaptoquinazolinone as the Cap for the Treatment of Cancer

List of supplement material

S1. Chemistry..2

S2. Antipreliferation of compounds on different cell lines……………………………..…..……12

S3. Detailed description for docking studies………………………………………….…….13

S4. Scanned NMR spectra of compounds (**1-15**)..14

S1. Chemistry

S1.1 Preparation of quinazolinone based hydroxamates (1-10)

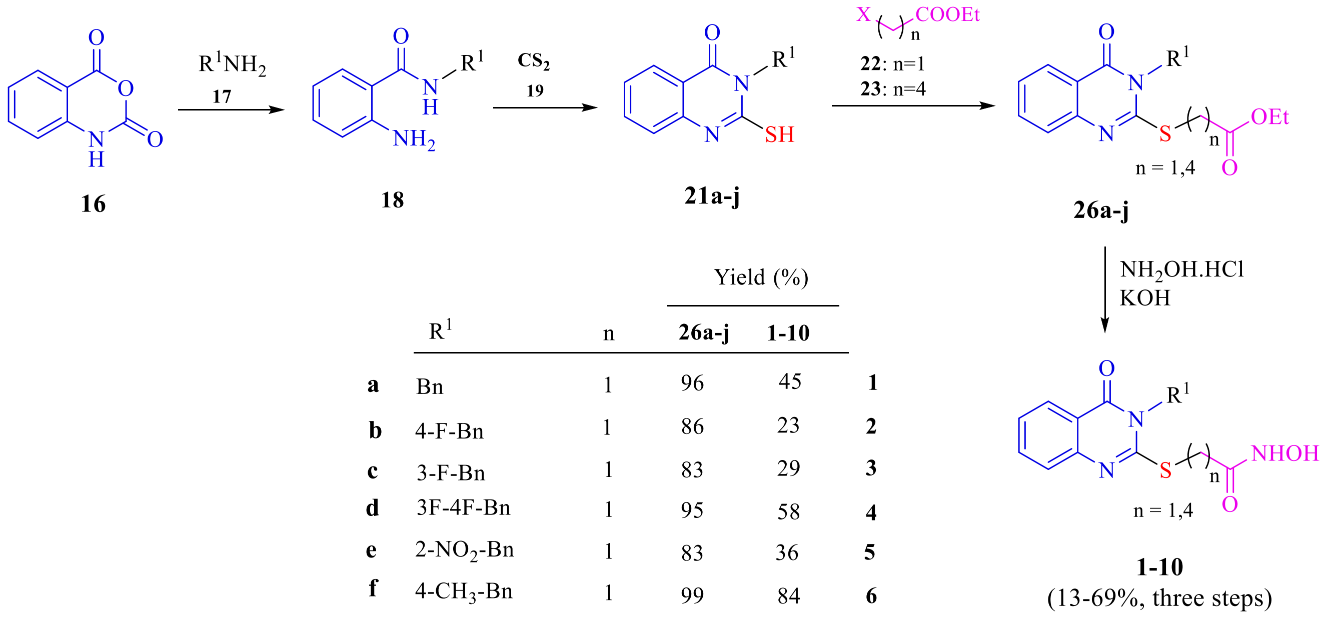

	R^1	n	Yield (%) 26a-j	Yield (%) 1-10	
**a**	Bn	1	96	45	**1**
**b**	4-F-Bn	1	86	23	**2**
**c**	3-F-Bn	1	83	29	**3**
**d**	3F-4F-Bn	1	95	58	**4**
**e**	2-NO_2-Bn	1	83	36	**5**
**f**	4-CH_3-Bn	1	99	84	**6**
**g**	CH_2-3-Pyridine	4	90	44	**7**
**h**	4-F-Bn	4	94	62	**8**
**i**	3F-4F-Bn	4	91	68	**9**
**j**	4-phenyl-Bn	4	87	46	**10**

Scheme S1. Synthesis of quinazolinone based hydroxamates (**1-10**)

General procedure for the synthesis of 2-mercaptoquinazolinone (21a-j)

A mixture of isatoic anhydride **16** (2 mmol) and amines (2.4 mmol) in water (10 mL) was stirred at room temperature for 3-4 hrs. After completion of the reaction, solid was precipitated which was filtered, washed with water and dried to give the corresponding 2-amino-*N*-arylbenzamide derivatives **18** which was used for the next step without further purification.

To a solution of KOH (1.5 mmol) in ethanol (10 mL) was added the intermediates **18** (1.5 mmol) followed by carbon disulfide (3 mmol). The reaction mixture was stirred at 80°C for 24 hrs. After completion of the reaction (monitored by TLC), excess solvent was removed under reduced

pressure and the residue was dissolved in water (10 mL) and then acidified with dilute HCl (10%) to pH 7, which led to the formation of a solid. The solid was filtered off, washed with water and dried to give the corresponding 2-mercaptoquinazolinone derivatives (**21a-j**) which was used for the next step without further purification.

General procedure for the synthesis of ester (26a-j)

The intermediates 2-mercaptoquinazolinone **(21a-j)** (0.8 mmol) was dissolved in 7 mL of acetone, then K_2CO_3 (1.2 mmol) was added and the mixture was stirred at room temperature for 15 min. Then ester (**22**) or (**23**) (0.96 mmol) was added slowly and the resulting mixture was stirred at 80°C for 2 hrs. After completion of the reaction (monitored by TLC), the solvent was evaporated under reduced pressure and the resulting mixture was poured into water (10 mL), neutralized with 5% HCl solution and the aqueous phase was extracted with ethyl acetate (3×10 mL). The combined organic layers were washed with a solution of saturated NaCl, dried over anhydrous Na_2SO_4 and filtered. After removal of the solvent under reduced pressure, the residue was purified by silica gel column chromatography give the corresponding intermediate esters **(26a-j)**.

General procedure for the synthesis of hydroxamate (1-10)

The solution of $NH_2OH.HCl$ (6 mmol) in ethanol (5 mL) was stirred at 0°C for 15 min, then KOH (7.5 mmol) was added. After stirring the mixture for further 15 min, the solution of intermediates **(26a-j)** (0.3 mmol) in ethanol (2 mL) was dropped into the mixture and the reaction mixture was stirred for 1-2 hrs. At the end of this reaction, excess solvent was removed under reduced pressure. The resulting mixture was poured into water (10 mL), neutralized with a 10% solution of HCl to pH~7, extracted with ethyl acetate (3×10 mL). The combined organic layers were washed with a solution of saturated NaCl, dried over anhydrous Na_2SO_4 and filtered. After removal of the solvent

under reduced pressure, the residue was purified by silica gel column chromatography to obtain the desired quinazolinone based hydroxamates (**1-10**).

2-((3-Benzyl-4-oxo-3,4-dihydroquinazolin-2-yl)thio)-*N*-hydroxyacetamide (1): Yield 32% as white solid. Mp: 188-191 °C. FT-IR (KBr) *V*max (cm^{-1}): 3488, 3307, 1672, 1552, 1473. HR-ESI-MS found *m/z* 342.0862 (calcd. 342.0834, $C_{17}H_{15}N_3O_3S$). ^{1}H-NMR (500 MHz, DMSO-d_6, δ ppm): 10.78 (*s*, 1H), 9.00 (*s*, 1H), 8.12 (*dd*, J_1 = 7.5 Hz, J_2 = 1.0 Hz, 1H), 7.84 (*td*, J_1 = 7.8 Hz, J_2 = 1.5 Hz, 1H), 7.62 (*d*, *J* = 8.5 Hz, 1H), 7.50 (*t*, *J* = 8.0 Hz, 1H), 7.32-7.37 (*m*, 2H), 7.28-7.30 (*m*, 2H), 5.35 (*s*, 2H), 3.91 (*s*, 2H). ^{13}C-NMR (125 MHz, DMSO-d_6, δ ppm): 163.7, 160.8, 156.2, 146.7, 135.5, 134.8, 128.6, 127.4, 126.7, 126.5, 126.1, 118.6, 46.8, 33.3.

2-((3-(4-Fluorobenzyl)-4-oxo-3,4-dihydroquinazolin-2-yl)thio)-*N*-hydroxyacetamide (2) (H13): Yield 13% as white solid. Mp: 178-181 °C. FT-IR (KBr) *V*max (cm^{-1}): 3466, 3261, 1674, 1555, 1472. HR-ESI-MS tìm found *m/z* 360.0819 (calcd. 360.0740, $C_{17}H_{14}FN_3O_3S$). ^{1}H-NMR (500 MHz, DMSO-d_6, δ ppm): 10.78 (*s*, 1H), 9.00 (*s*, 1H), 8.11 (*dd*, J_1 = 8.0 Hz, J_2 = 1.0 Hz, 1H), 7.83 (*td*, J_1 = 7.8 Hz, J_2 = 1.5 Hz, 1H), 7.60 (*d*, *J* = 8.0 Hz, 1H), 7.49 (*td*, J_1 = 7.8 Hz, J_2 = 1.0 Hz, 1H), 7.34-7.37 (*m*, 2H), 7.15-7.19 (*m*, 2H), 5.32 (*s*, 2H), 3.91 (*s*, 2H). ^{13}C-NMR (125 MHz, DMSO-d_6, δ ppm): 163.7, 162.4, 160.8, 160.5, 156.0, 166.7, 134.8, 131.8, 129.1, 129.0, 126.5, 126.1, 126.1, 118.6, 115.4, 115.3, 46.2, 33.3.

2-((3-(3-Fluorobenzyl)-4-oxo-3,4-dihydroquinazolin-2-yl)thio)-*N*-hydroxyacetamide (3): Yield 15% as white solid. Mp: 177-180 °C. FT-IR (KBr) *V*max (cm^{-1}): 3375, 3230, 1673, 1556, 1474. HR-ESI-MS found *m/z* 360.0820 $[M+H]^+$ (calcd. 360.0740, $C_{17}H_{14}FN_3O_3S$). ^{1}H-NMR (500 MHz, DMSO-d_6, δ ppm): 10.78 (*s*, 1H), 9.00 (*s*, 1H), 8.11 (*dd*, J_1 = 7.8 Hz, J_2 = 1.5 Hz, 1H), 7.84 (*td*, J_1 = 7.8 Hz, J_2 = 1.5 Hz, 1H), 7.61 (*d*, *J* = 8.5 Hz, 1H), 7.47-7.50 (*m*, 1H), 7.36-7.41 (*m*, 1H), 7.10-7.15 (*m*, 3H), 5.35 (*s*, 2H), 3.91 (*s*, 2H). ^{13}C-NMR (125 MHz, DMSO-d_6, δ ppm): 163.2,

161.2, 160.9, 156.0, 146.7, 138.5, 138.5, 134.9, 130.7, 130.6, 126.6, 126163.7,.2, 126.1, 122.7, 122.7, 118.7, 114.4, 114.2, 123.8, 113.7.

2-((3-(3,4-difluorobenzyl)-4-oxo-3,4-dihydroquinazolin-2-yl)thio)-N-hydroxyacetamide (4): Yield 44% as white solid. Mp: 184-186°C. FT-IR (KBr) *V*max (cm^{-1}): 3375, 3230, 1673, 1556, 1474. HR-ESI-MS found *m/z* 378.0665 $[M+H]^+$ (calcd. 378.0646, $C_{17}H_{13}F_2N_3O_3S$). ^{1}H-NMR (600 MHz, DMSO-d_6, δ ppm): 10.80 (*s*, 1H), 9.01 (*s*, 1H), 8.10 (*dd*, J_1 = 7.8 Hz, J_2 = 1.2 Hz, 1H), 7.84 (*td*, J_1 = 7.5 Hz, J_2 = 1.2 Hz, 1H), 7.60 (*d*, *J* = 7.8 Hz, 1H), 7.49 (*td*, J_2 = 7.8 Hz, J_2 = 1.2 Hz, 1H), 7.38-7.45 (*m*, 2H), 7.13-7.14 (*m*, 1H), 5.31 (*s*, 2H), 3.91 (*s*, 2H). ^{13}C-NMR (150 MHz, DMSO-d_6, δ ppm): 163.7, 160.9, 155.9, 150.2, 150.1, 149.6, 149.5, 148.5, 148.4, 148.0, 147.9, 146.7, 134.9, 133.4, 126.6, 126.2, 126.1, 123.8, 123.7, 123.7, 118.7, 117.7, 117.6, 116.4, 116.3, 46.0, 33.3.

***N*-Hydroxy-2-((3-(2-nitrobenzyl)-4-oxo-3,4-dihydroquinazolin-2-yl)thio)acetamide (5):** Yield 20% as white solid. Mp: 176-179°C. FT-IR (KBr) *V*max (cm^{-1}): 3455, 3261, 1676, 1554, 1472. HR-ESI-MS found *m/z* 387.0759 $[M+H]^+$ (calcd. 387.0685, $C_{17}H_{14}N_4O_5S$). ^{1}H-NMR (500 MHz, DMSO-d_6, δ ppm): 10.78 (*s*, 1H), 8.99 (*s*, 1H), 8.19 (*dd*, J_1 = 8.0 Hz, J_2 = 1.0 Hz, 1H), 8.11 (*dd*, J_1 = 8.0 Hz, J_2 = 1.5 Hz, 1H), 7.86 (*td*, J_1 = 7.8 Hz, J_2 = 1.5 Hz, 1H), 7.68 (*dd*, J_1 = 7.5 Hz, J_2 = 1.5 Hz, 1H), 7.64 (*d*, *J* = 8.0 Hz, 2H), 7.60 (*dd*, J_1 = 7.8 Hz, J_2 = 1.0 Hz, 1H), 7.51 (*td*, J_1 = 7.5 Hz, J_2 = 1.0 Hz, 1H), 7.15 (*d*, *J* = 7.5 Hz, 1H), 5.64 (*s*, 2H), 3.91 (*s*, 2H). ^{13}C-NMR (125 MHz, DMSO-d_6, δ ppm): 163.5, 160.9, 155.8, 147.6, 146.7, 135.0, 134.5, 130.5, 128.9, 127.0, 126.6, 126.3, 126.2, 125.3, 118.6, 44.6, 33.2.

***N*-hydroxy-2-((3-(4-methylbenzyl)-4-oxo-3,4-dihydroquinazolin-2-yl)thio)acetamide (6):** Yield 69% as white solid. Mp: 184-186 °C. FT-IR (KBr) *V*max (cm^{-1}): 3467, 3263, 1655, 1554, 1474. HR-ESI-MS found *m/z* 356.1070 $[M+H]^+$ (calcd. 356.0991, $C_{18}H_{17}N_3O_3S$). ^{1}H-NMR (500 MHz, DMSO-d_6, δ ppm): 10.78 (*s*, 1H), 9.00 (*s*, 1H), 8.11 (*d*, *J* = 8.0 Hz, 1H), 7.83 (*t*, *J* = 8.0 Hz,

1H), 7.61 (*d*, *J* = 8.0 Hz, 1H), 7.48 (*t*, *J* = 7.5 Hz, 1H), 7.13-7.19 (*m*, 4H), 5.29 (*s*, 2H), 3.89 (*s*, 2H), 2.27 (*s*, 3H). ^{13}C-NMR (125 MHz, DMSO-*d*6, *δ* ppm): 163.7, 160.8, 156.2, 146.7, 136.6, 134.8, 132.5, 129.1, 126.8, 126.5, 126.1, 118.7, 46.6, 33.3, 20.6.

***N*-Hydroxy-5-((4-oxo-3-(pyridin-3-ylmethyl)-3,4-dihydroquinazolin-2-yl)thio)pentanamide (7):** Yield 23% as white solid. Mp 166-168°C. FT-IR (KBr) *V*max (cm^{-1}): 3234, 3022, 2941, 1677, 1544, 1473. HR-ESI-MS found *m/z* 385.1331 [M+H]$^+$ (calcd. 385.1256, $C_{19}H_{20}N_4O_3S$). ^{1}H-NMR (400 MHz, DMSO-*d*6, *δ* ppm): 10.32 (*s*, 1H), 8.64 (*s*, 1H), 8.55 (*s*, 1H), 8.45 (*d*, *J* = 4.0 Hz, 1H), 8.09 (*d*, *J* = 8.0 Hz, 1H), 7.80 (*s*, 1H), 7.64 (*d*, *J* = 8.0 Hz, 1H), 7.57 (*d*, *J* = 8.0 Hz, 1H), 7.46 (*s*, 1H), 7.34 (*s*, 1H), 5.33 (*s*, 2H), 3.24 (*s*, 2H), 1.98 (*s*, 2H), 1.60-1.66 (*m*, 4H). ^{13}C-NMR (100 MHz, DMSO-*d*6, *δ* ppm): 168.7, 162.3, 160.9, 160.4, 156.6, 146.8, 134.8, 131.9, 129.0, 128.9, 126.5, 126.0, 125.9, 118.7, 115.4, 115.2, 46.1, 31.7, 31.2, 27.8, 24.2.

5-((3-(4-Fluorobenzyl)-4-oxo-3,4-dihydroquinazolin-2-yl)thio)-*N*-hydroxypentanamide (8): Yield 44% as white solid. Mp 138-140 °C. FT-IR (KBr) *V*max (cm^{-1}): 3244, 3057, 2922, 1672, 1550, 1474. HR-ESI-MS found *m/z* 402.1283 [M+H]$^+$ (calcd. 402.1209, $C_{20}H_{20}FN_3O_3S$). ^{1}H-NMR (500 MHz, DMSO-*d*6, *δ* ppm): 10.34 (*s*, 1H), 8.66 (*s*, 1H), 8.11 (*dd*, J_1 = 7.5 Hz, J_2 = 1.0 Hz, 1H), 7.81 (*td*, J_1 = 7.75 Hz, J_2 = 1.5 Hz, 1H), 7.58 (*d*, *J* = 8.5 Hz, 1H), 7.46 (*t*, *J* = 7.5 Hz, 1H), 7.31-7.33 (*m*, 2H), 7.16 (*t*, *J* = 9.0 Hz, 2H), 5.30 (*s*, 2H), 3.25 (*t*, *J* = 7.0 Hz, 2H), 2.00 (*t*, *J* = 7.0 Hz, 2H), 1.60-1.69 (*m*, 4H). ^{13}C-NMR (125 MHz, DMSO-*d*6, *δ* ppm): 168.7, 162.3, 160.9, 160.4, 156.6, 146.8, 134.8, 131.9, 129.0, 128.9, 126.5, 126.0, 125.9, 118.7, 115.4, 115.2, 46.1, 31.7, 31.2, 27.8, 24.2

5-((3-(3-Fluorobenzyl)-4-oxo-3,4-dihydroquinazolin-2-yl)thio)-*N*-hydroxypentanamide (9): Yield 51% as white solid. Mp 145-147 °C. FT-IR (KBr) *V*max (cm^{-1}): 3251, 3060, 2950, 1671, 1550, 1473. HR-ESI-MS found *m/z* 420.1189 [M+H]$^+$ (calcd. 420.1115, $C_{20}H_{19}F_2N_3O_3S$). ^{1}H-

NMR (600 MHz, DMSO-d_6, δ ppm): 10.34 (*s*, 1H), 8.66 (*s*, 1H), 8.10 (*dd*, J_1 = 7.8 Hz, J_2 = 1.2 Hz, 1H), 7.80-7.83 (*m*, 1H), 7.59 (*d*, J = 7.8 Hz, 1H), 7.48 (*td*, J_1 = 8.1 Hz, J_2 = 1.2 Hz, 1H), 7.36-7.40 (*m*, 2H), 7.08-7.09 (*m*, 1H), 5.30 (*s*, 2H), 3.25 (*t*, J = 7.2 Hz, 2H), 1.99 (*t*, J = 7.2 Hz, 2H), 1.68 (*quin*, J = 7.2 Hz, 2H), 1.62 (*quin*, J = 7.2 Hz, 2H). ^{13}C-NMR (125 MHz, DMSO-d_6, δ ppm): 168.8, 161.0, 156.5, 146.9, 134.9, 134.9, 126.6, 126.1, 123.6, 118.7, 117.7, 117.6, 116.3, 116.2, 45.9, 31.7, 31.3, 27.9, 24.3.

5-((3-([1,1'-Biphenyl]-4-ylmethyl)-4-oxo-3,4-dihydroquinazolin-2-yl)thio)-*N*-hydroxypentanamide (10): Yield 25% as white solid. Mp 180-182°C. FT-IR (KBr) *V*max (cm^{-1}): 3450, 3043, 2940, 1676, 1544, 1471. HR-ESI-MS found *m/z* 460.1695 $[M+H]^+$ (calcd. 460.1617, $C_{26}H_{25}N_3O_3S$). ^{1}H-NMR (600 MHz, DMSO-d_6, δ ppm): 10.34 (*s*, 1H), 8.66 (*s*, 1H), 8.10 (*dd*, J_1 = 8.1 Hz, J_2 = 1.8 Hz, 1H), 7.82 (*td*, J_1 = 7.8 Hz, J_2 = 1.8 Hz, 1H), 7.59-7.63 (*m*, 5H), 7.48 (*td*, J_1 = 7.8 Hz, J_2 = 1.2 Hz, 1H), 7.45 (*t*, J = 8.4, 2H), 7.33-7.36 (*m*, 3H), 5.30 (*s*, 2H), 3.26 (*t*, J = 7.2 Hz, 2H), 1.99 (*t*, J = 7.2 Hz, 2H), 1.69 (*quin*, J = 7.2 Hz, 1H), 1.63 (*quin*, J = 7.2 Hz, 1H). ^{13}C-NMR (150 MHz, DMSO-d_6, δ ppm): 168.8, 161.0, 156.8, 146.9, 139.7, 139.3, 134.9, 134.9, 134.9, 128.9, 127.5, 127.3, 126.9, 126.6, 126.1, 126.0, 118.7, 46.6, 31.7, 31.3, 30.7, 27.9, 24.3.

S1.2 Preparation of quinazolinone based hydroxamates (11-12)

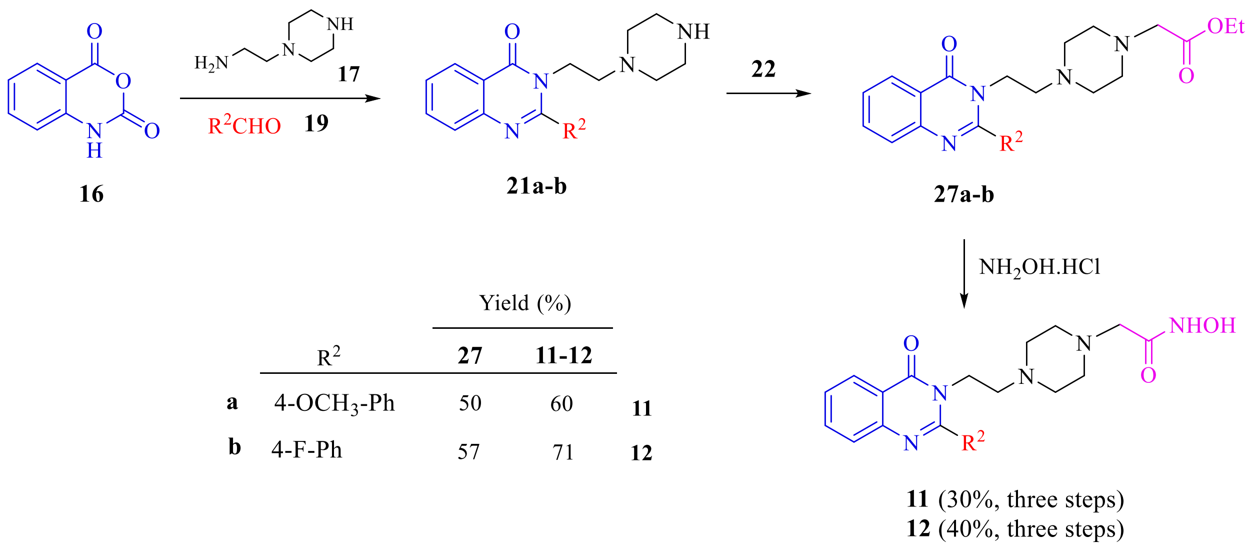

		Yield (%)		
	R^2	**27**	**11-12**	
**a**	4-OCH_3-Ph	50	60	**11**
**b**	4-F-Ph	57	71	**12**

Scheme S2. Synthesis of quinazolinone based hydroxamates (**11-12**)

General procedure for the synthesis of (21a-b)

A mixture of isatoic anhydride **16** (2 mmol) and amine **17** (2.4 mmol) in water (10 mL) was stirred at room temperature for 30 min, then aldehyde **19** was added. After stirring the mixture for further 15 min, iron (III) chloride hexahydrates (4 mmol) was added and the resulting mixture was stirred at 100°C for 3 hrs. After completion of the reaction as indicated by TLC, the mixture was cooled to room temperature. A solution of saturated $NaHCO_3$ was gradually added to adjust pH to 7. The mixture was extracted with ethyl acetate (3×20 mL). The combined organic layers were washed with a solution of saturated NaCl, dried over anhydrous Na_2SO_4 and filtered. After removal of the solvent under reduced pressure, the quinazolinone (**21a-b**) was obtained and used for the next step without further purification.

General procedure for the synthesis of ester (27a-b)

The intermediate quinazolinone **21a-b** obtained from the first step was dissolved in 10 mL of acetone, then K_2CO_3 (4 mmol) was added and the mixture was stirred at room temperature for 10 min. Then ester **22** (2.6 mmol) was dropped slowly into the reaction mixture. The resulting mixture was stirred at 75°C for 2 hrs. After completion of the reaction (monitored by TLC), the solvent was evaporated under reduced pressure and the obtained mixture was poured into water (10 mL). The aqueous phase was extracted with ethyl acetate (3×20 mL). The combined organic layers were washed with a solution of saturated NaCl, dried over anhydrous Na_2SO_4 and filtered. After removal of the solvent under reduced pressure, the residue was purified by silica gel column chromatography to provide the corresponding esters (**27a-b**).

General procedure for the synthesis of quinazolinone based hydroxamates (11-12)

A solution of $NH_2OH.HCl$ (16.65 mmol) in ethanol (10 mL) was stirred at 0°C for 15 min, then KOH (19.4 mmol) was added. After stirring the mixture for further 15 min, the solution of ester **(27a-b**) (0.5 mmol) in ethanol (2 mL) was dropped into the mixture. The resulting reaction mixture was stirred at 0°C for 2 hrs. At the end of the reaction, excess solvent was removed under reduced pressure. The obtained mixture was poured into water (10 mL), neutralized with a solution of HCl (1M) to pH 7, extracted with ethyl acetate (3×20 mL). The combined organic layers were washed with a solution of saturated NaCl, dried over anhydrous Na_2SO_4 and filtered. After removal of the solvent under reduced pressure, the residue was purified by silica gel column chromatography to obtain the desired products (**11-12**).

N-Hydroxy-2-(4-(2-(4-methoxyphenyl)-4-oxoquinazolin-3(4H)-yl)ethyl)piperazin-1-yl)acetamide (11): Yield 30% as white solid. Mp 120-122°C. FT-IR (KBr) V*max* (cm^{-1}): 3554,

3283, 2939, 2821, 1680, 1609, 1513, 1338, 1025, 779. HR-ESI-MS found m/z 438.2120 $[M+H]^+$ (calcd. 438.2063, $C_{23}H_{27}N_5O_4$). ^{1}H-NMR (500 MHz, DMSO-d_6, δ ppm): 10.30 (s, 1H), 8.71 (s, 1H), 8.17 (dd, J_1 = 8.0 Hz, J_2 = 1.0 Hz, 1H), 7.81-7.84 (m, 1H), 7.65 (d, J = 8.0 Hz, 1H), 7.58-7.61 (m, 2H), 7.53-7.56 (m, 1H), 7.07-7.10 (m, 2H), 4.08 (t, J = 6.5 Hz, 2H), 3.84 (s, 3H), 2.77 (s, 2H), 2.40 (t, J = 7.0 Hz, 2H), 2.27 (s, 4H), 2.15 (s, 4H). ^{13}C-NMR (125 MHz, DMSO-d_6, δ ppm): 165.61, 161.41, 160.03 155.98, 146.88, 134.37, 129.91, 127.70, 127.06, 126.73, 126.13, 120.17, 113.59, 58.96, 55.33, 55.26, 52.58, 52.41, 42.47.

2-(4-(2-(4-Fluorophenyl)-4-oxoquinazolin-3(4H)-yl)ethyl)piperazin-1-yl)N-hydroxyacetamide (12): Yield 40% as white solid. Mp 134-136°C. FT-IR (KBr) ν_{max} (cm^{-1}): 3477, 3190, 2836, 1667, 1606, 1338, 1228, 1047, 775. HR-ESI-MS found m/z 426.1940 $[M+H]^+$ (calcd. 426.1863, $C_{22}H_{24}FN_5O_3$). ^{1}H-NMR (500 MHz, DMSO-d_6, δ ppm): 10.30 (s, 1H), 8.71 (s, 1H), 8.18 (dd, J_1 = 8.0 Hz, J_2 = 1.0 Hz, 1H), 7.82-7.86 (m, 1H), 7.72-7.75 (m, 2H), 7.67 (d, J = 8.0 Hz, 1H), 7.55-7.58 (m, 1H), 7.36-7.41 (m, 2H), 4.02 (t, J = 7.5 Hz, 2H), 3.84 (s, 3H), 2.78 (s, 2H), 2.40 (t, J = 7.0 Hz, 2H), 2.27 (s, 4H), 2.14 (s, 4H). ^{13}C-NMR (125 MHz, DMSO-d_6, δ ppm): 165.65, 163.49, 161.53, 161.25, 155.23, 146.75, 134.50, 131.87, 130.88, 130.81, 127.13, 127.03, 126.17, 120.34, 115.40, 115.22, 59.72, 58.96, 55.24, 52.56, 52.41, 42.50.

S1.3 Preparation of quinazolinone based hydroxamates (13-15)

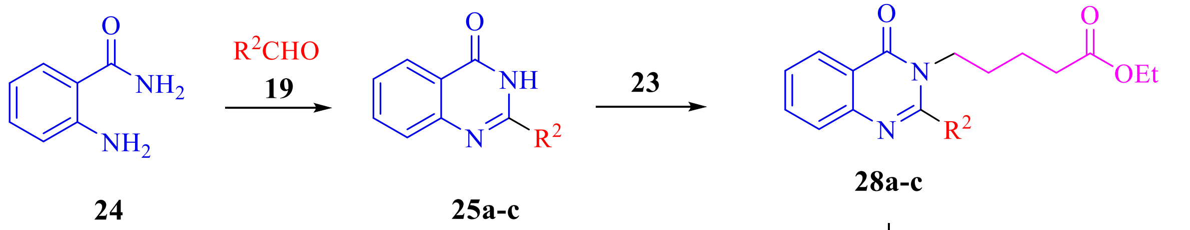

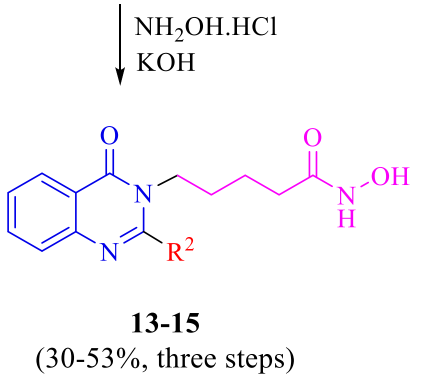

	R^2	Yield (%) 28a-c	13-15	
**a**	4-OCH_3-Ph	91	50	**13**
**b**	4-F-Ph	89	59	**14**
**c**	2-Furanyl	70	43	**15**

Scheme S3. Synthesis of quinazolinone based hydroxamates (**13-15**)

General procedure for the synthesis of (25a-c)

A mixture of anthranilamide **24** (2 mmol) and aldehyde **19** (2.4 mmol) in DMSO was stirred at 120°C for 24 hrs in the open air. After completion of the reaction (monitored by TLC), the resulting mixture was poured into water (100 mL), which led to the formation of white solid. The solids were filtered, washed with water and dried to give the corresponding quinazolinone derivatives **(25a-c)**, which was used for the next step without further purification.

General procedure for the synthesis of ester (28a-c)

The intermediates **(25a-c)** (1 mmol) and K_2CO_3 (2.5 mmol) was dissolved in 7 mL of acetone, then ethyl 5-bromopentanoate **(23)** (1.5 mmol) was dropped slowly into the mixture. The reaction mixture was stirred at 70°C for 24 hrs. After completion of the reaction (monitored by TLC), the solvent was evaporated under reduced pressure and the resulting mixture was poured into water

(10 mL), neutralized with saturated NH_4Cl solution and the aqueous phase was extracted with ethyl acetate (3×20 mL). The combined organic layers were washed with a solution of saturated NaCl, dried over anhydrous Na_2SO_4 and filtered. After removal of the solvent under reduced pressure, the residue was purified by silica gel column chromatography to afford the corresponding intermediate esters **(28a-c)**.

General procedure for the synthesis of hydroxamates (13-15)

A solution of NH_2OH.HCl (16.65 mmol) in ethanol (10 mL) was stirred at 0°C for 15 min, then KOH (19.4 mmol) was added. After stirring the mixture for further 20 min, the solution of intermediates **(28a-c)** (0.5 mmol) in ethanol (2 mL) was added into the mixture. The reaction mixture was stirred at 0°C for 2 hrs. At the end of the reaction, excess solvent was removed under reduced pressure. The resulting mixture was poured into water (10 mL), neutralized with saturated NH_4Cl to pH 7, extracted with ethyl acetate (3×20 mL). The combined organic layers were washed with a solution of saturated NaCl, dried over anhydrous Na_2SO_4 and filtered. After removal of the solvent under reduced pressure, the residue was purified by silica gel column chromatography to afford the desired products **(13-15)**.

***N*-Hydroxy-5-(2-(4-methoxyphenyl)-4-oxoquinazolin-3(4*H*)-yl)pentanamide (13):** Yield 46% as white solid. Mp: 173-175°C. FT-IR (KBr) ν_{max} (cm^{-1}): 3637, 3252, 1638, 1607, 1576, 1241. HR-ESI-MS found *m/z* 368.1553 $[M+H]^+$ (calcd. 368.1532, $C_{20}H_{21}N_3O_4$). ^{1}H-NMR (600 MHz, DMSO-d_6, δ ppm): 10.39 (*s*, 1H), 8.69 (*s*, 1H), 8.47 (*dt*, J_1 = 9.0 Hz, J_2 = 2.5 Hz, 2H), 8.12 (*d*, J = 8.0 Hz, 1H), 7.91 (*d*, J = 3.0 Hz, 2H), 7.58-7.61 (*m*, 1H), 7.09 (*dd*, J_1 = 7.0 Hz, J_2 = 2.0 Hz, 2H), 4.69 (*t*, J = 6.5 Hz, 2H), 3.85 (*s*, 3H), 2.08 (*t*, J = 7.5 Hz, 2H), 1.88-1.91 (*m*, 2H), 1.74-1.77 (*m*, 2H). ^{13}C-NMR (125 MHz, DMSO-d_6, δ ppm): 168.9, 166.1, 161.5, 158.8, 151.3, 134.1, 129.9, 129.7, 127.4, 126.6, 123.2, 114.3, 113.9, 66.3, 55.3, 31.9, 27.8, 21.8.

5-(2-(4-Fluorophenyl)-4-oxoquinazolin-3(4*H*)-yl)-*N*-hydroxypentanamide (14): Yield 53% as white solid. Mp: 156-158°C. FT-IR (KBr) ν_{max} (cm^{-1}): 3624, 3212, 1658, 1623, 1577, 1013. HR-ESI-MS found *m/z* 356.1412 $[M+H]^+$ (calcd. 356.1332, $C_{19}H_{18}FN_3O_3$). ^{1}H-NMR (600 MHz, DMSO-d_6, δ ppm): 10.38 (*s*, 1H), 8.68 (*s*, 1H), 8.55-8.58 (*m*, 2H), 8.16 (*d*, *J* = 8.0 Hz, 1H), 7.94-7.96 (*m*, 2H), 7.63-7.66 (*m*, 1H), 7.35-7.39 (*m*, 2H), 4.71 (*t*, *J* = 6.0 Hz, 2H), 2.09 (*t*, *J* = 7.5 Hz, 2H), 1.88-1.91 (*m*, 2H), 1.75-1.79 (*m*, 2H). ^{13}C-NMR (125 MHz, DMSO-d_6, δ ppm): 168.9, 166.3, 164.9, 162.9, 158.0, 151.1, 134.3, 133.9, 130.4, 130.4, 127.5, 127.1, 123.2, 115.6, 115.4, 114.5, 66.5, 31.9, 27.7, 21.8.

5-(2-(Furan-2-yl)-4-oxoquinazolin-3(4*H*)-yl)-*N*-hydroxypentanamide (15): Yield 30% as white solid. Mp: 195-197°C. FT-IR (KBr) ν_{max} (cm^{-1}): 3649, 3184, 1640, 1595, 1559. HR-ESI-MS found *m/z* 328.1296 $[M+H]^+$ (calcd. 328.1219, $C_{17}H_{17}N_3O_4$). ^{1}H-NMR (600 MHz, DMSO-d_6, δ ppm): 10.39 (*s*, 1H), 8.69 (*s*, 1H), 8.11 (*d*, *J* = 7.8 Hz, 1H), 7.89-7.93 (*m*, 3H), 7.60-7.63 (*m*, 1H), 7.38-7.39 (*m*, 1H), 6.71 (*dd*, J_1 = 3.6 Hz, J_2 = 1.8 Hz, 1H) , 4.63 (*t*, *J* = 6.0 Hz, 2H), 2.07 (*t*, *J* = 7.2 Hz, 2H), 1.85-1.88 (*m*, 2H), 1.73-1.76 (*m*, 2H). ^{13}C-NMR (150 MHz, DMSO-d_6, δ ppm): 168.9, 166.2, 152.4, 152.0,151.0, 145.8, 134.3, 127.3, 127.0, 123.3, 114.6, 114.0, 112.4, 66.5, 31.9, 27.7, 21.8.

S2. Antipreliferation of compounds on different cell lines

Table S1. Antipreliferation of compounds on different cell lines

Tumor cell	Cell line	IC_{50} (μM)	
		Belinostat	8
Breast	MDA-MB 231	3.72±0.5	0.51±0.02
Breast	EMT 6	11.3±2.6	1.30±0.3
Brain	N2A	3.66±0.7	3.2±0.1
Liver	Hepa1c1c7	10.1±0.9	1.3±0.2
Ovarian	SKOV3	32.1±5.5	4.5±0.1
Colon	SW620	14.9±1.2	0.81±0.01
Pancreas	PANC1	15.7±2.3	12.8±0.75

S3. Detailed description for docking studies

The molecular docking study utilizes AutoDock4Zn with Lamarckian genetic algorithm (LGA) for searching the optimum dock pose together with scoring function to calculate the binding affinity. AutoDock Tools (ADT) was employed to set up and performed docking calculation.

In this study, we performed the docking study assuming that having a rigid protein and consider the conformational space of the ligands to analyze the inductive effect of the hybrid compounds. To turn the protein molecule into a free receptor, the heteroatoms including water molecules were deleted and polar hydrogen atoms and Kollman charges were added. All other bonds were allowed to be rotatable. In the docking analysis, the binding site was enclosed in a box with the number of grid points in x × y × z directions (64 × 64 × 64) and a grid spacing of 0.375 Å. Initially, AutoGrid was run to generate the grid map of various atoms of the ligands and receptor. After the completion of the grid map, AutoDock was run by using autodock parameters as follows: GA population size, 300; maximum number of energy evaluations, 2 500 000; and the number of generations, 27 000. A maximum of 50 conformers were considered for each molecule, and the root-mean-square (RMS) cluster tolerance was set to 2.0 Å in each run.

The outputs from AutoDock modeling studies were analyzed using PyMOL, Discovery Studio Visualizer. PyMOL was used to calculate the distances of hydrogen bonds as measured between the hydrogen and its assumed binding partner.

S4. Scanned NMR spectra of compounds (1-15)

PerkinElmer Spectrum 10.5.2

June 15, 2021 5:09

Report Details

Report Location	C:\Users\Administrator\Desktop\TTPT\KQ 2021\150621\QT5A C17H15N3O3S
Report Creator	Administrator
Report Date	June 15, 2021 5:09

Sample Details

Filename	C:\Users\Administrator\Desktop\TTPT\KQ 2021\150621\QT5A C17H15N3O3S.sp
Creation Date	6/15/2021 4:04:40 PM
Analyst	Administrator
X-Axis Units	cm-1
X-Axis start value	4000
X-Axis end value	400
Data interval	-1
Number of points	3601
Y-Axis Units	%T
Description	Sample 252 By Administrator Date Tuesday, June 15 2021

Spectrum

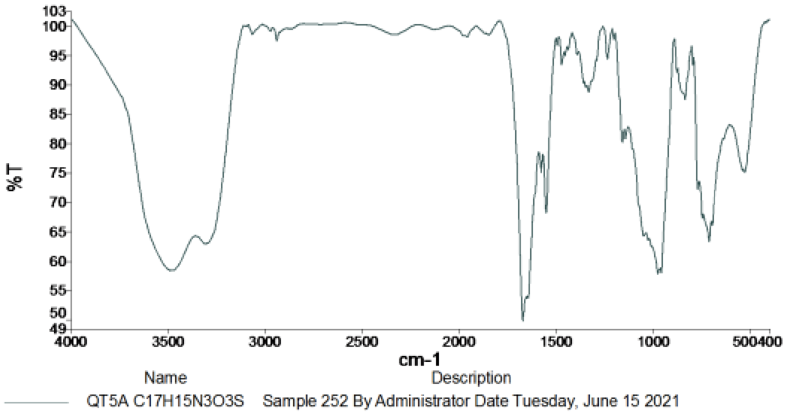

Figure S4.1 IR spectrum of compound **1**

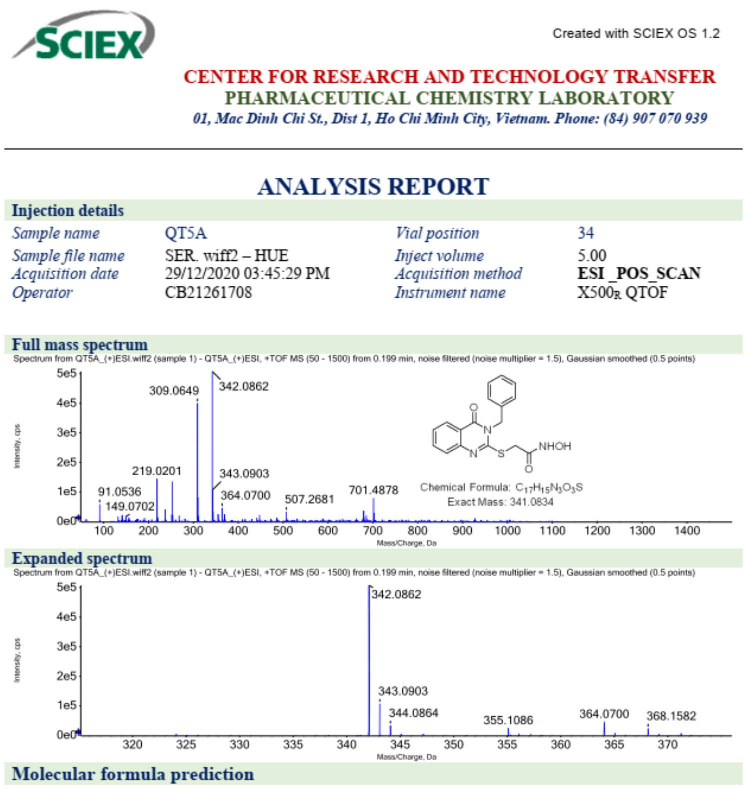

Figure S4.2 MS spectrum of compound **1**

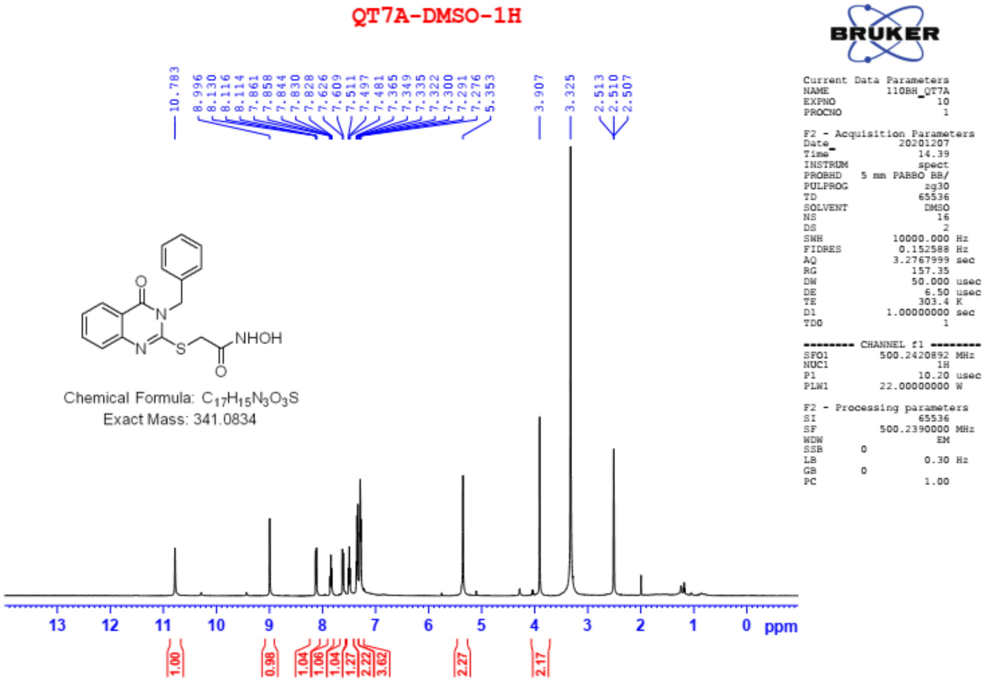

Figure S4.3 ^{1}H-NMR spectrum of compound **1**

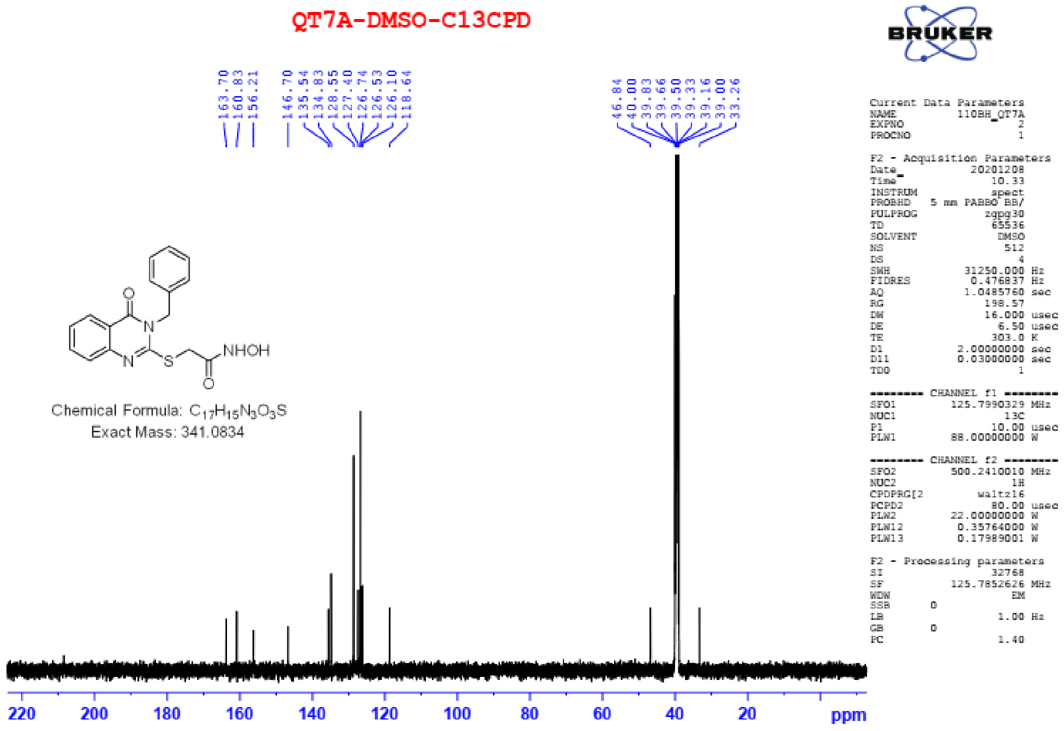

Figure S4.4 ^{13}C-NMR spectrum of compound **1**

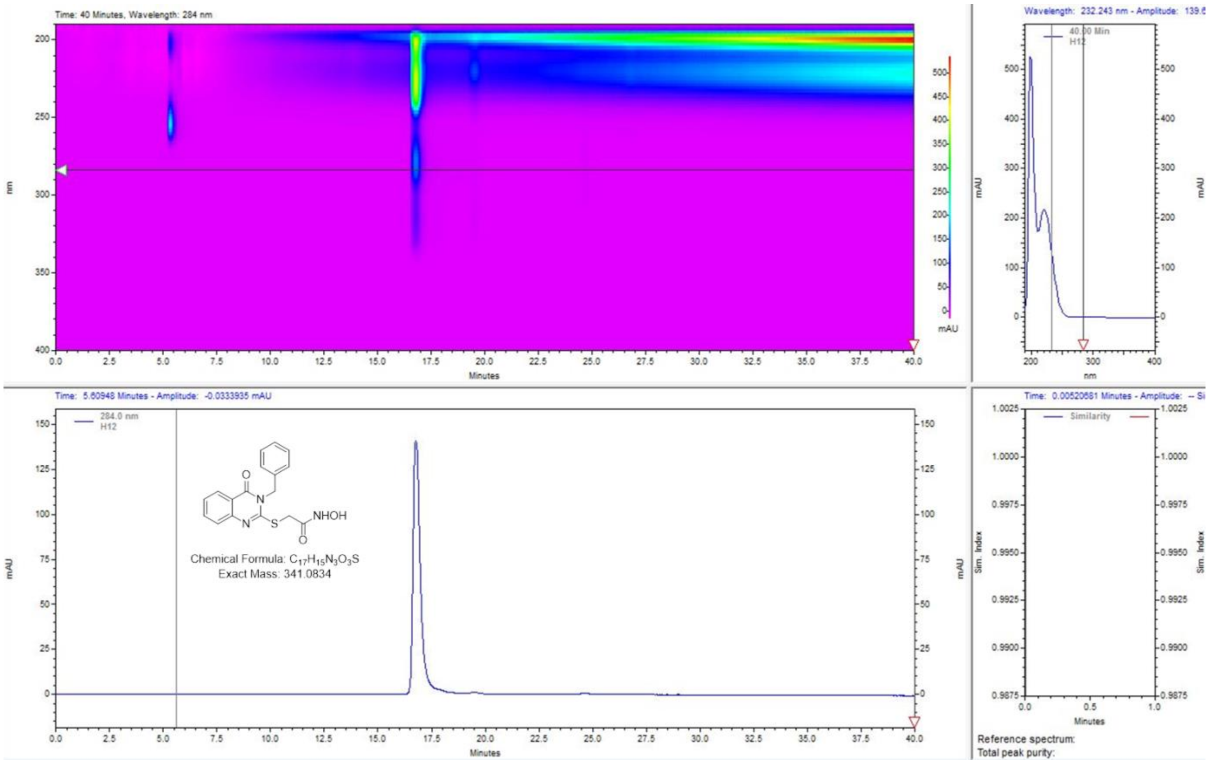

Figure S4.5 HPLC spectrum of compound **1**

PerkinElmer Spectrum 10.5.2

June 15, 2021 4:59

Report Details

Report Location	C:\Users\Administrator\Desktop\TTPT\KQ 2021\150621\QT5B C17H14FN3O
Report Creator	Administrator
Report Date	June 15, 2021 4:59

Sample Details

Filename	C:\Users\Administrator\Desktop\TTPT\KQ 2021\150621\QT5B C17H14FN3O.sp
Creation Date	6/15/2021 3:54:49 PM
Analyst	Administrator
X-Axis Units	cm-1
X-Axis start value	4000
X-Axis end value	400
Data interval	-1
Number of points	3601
Y-Axis Units	%T
Description	Sample 252 By Administrator Date Tuesday, June 15 2021

Spectrum

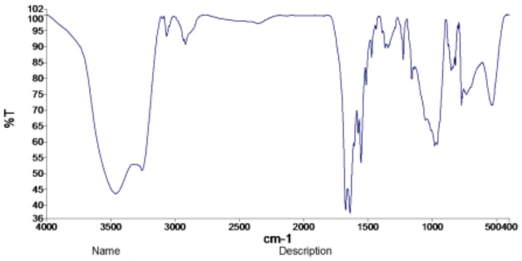

Name Description

QT5B C17H14FN3O Sample 252 By Administrator Date Tuesday, June 15 2021

Figure S4.6 IR spectrum of compound **2**

Created with SCIEX OS 1.2

CENTER FOR RESEARCH AND TECHNOLOGY TRANSFER

PHARMACEUTICAL CHEMISTRY LABORATORY

01, Mac Dinh Chi St., Dist 1, Ho Chi Minh City, Vietnam. Phone: (84) 907 070 939

ANALYSIS REPORT

Injection details

Sample name	QT5b	*Vial position*	24
Sample file name	SER. wiff2 – HUE	*Inject volume*	5.00
Acquisition date	04/11/2020 10:53:32 AM	*Acquisition method*	**ESI _POS_SCAN**
Operator	CB21261708	*Instrument name*	X500R QTOF

Full mass spectrum

Spectrum from QT5b_(+)ESI 2020-11-04-10-53-32.wiff2 (sample 1) - QT5b_(+)ESI, +TOF MS...m 0.171 min, noise filtered (noise multiplier = 1.5), Gaussian smoothed (0.5 points)

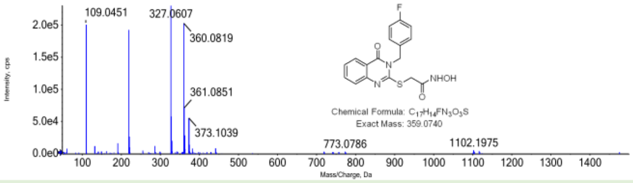

Expanded spectrum

Spectrum from QT5b_(+)ESI 2020-11-04-10-53-32.wiff2 (sample 1) - QT5b_(+)ESI, +TOF MS...m 0.171 min, noise filtered (noise multiplier = 1.5), Gaussian smoothed (0.5 points)

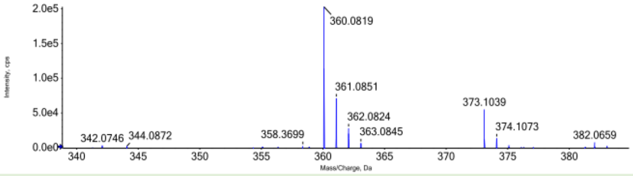

Molecular formula prediction

Figure S4.7 MS spectrum of compound **2**

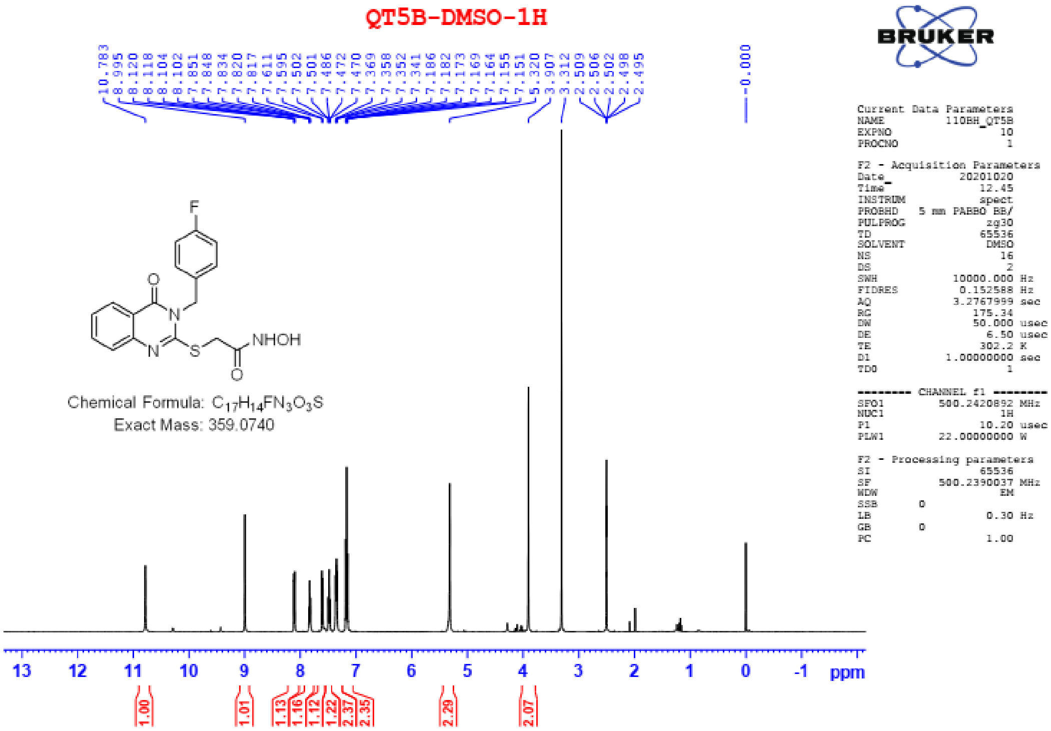

Figure S4.8 ^{1}H-NMR spectrum of compound **2**

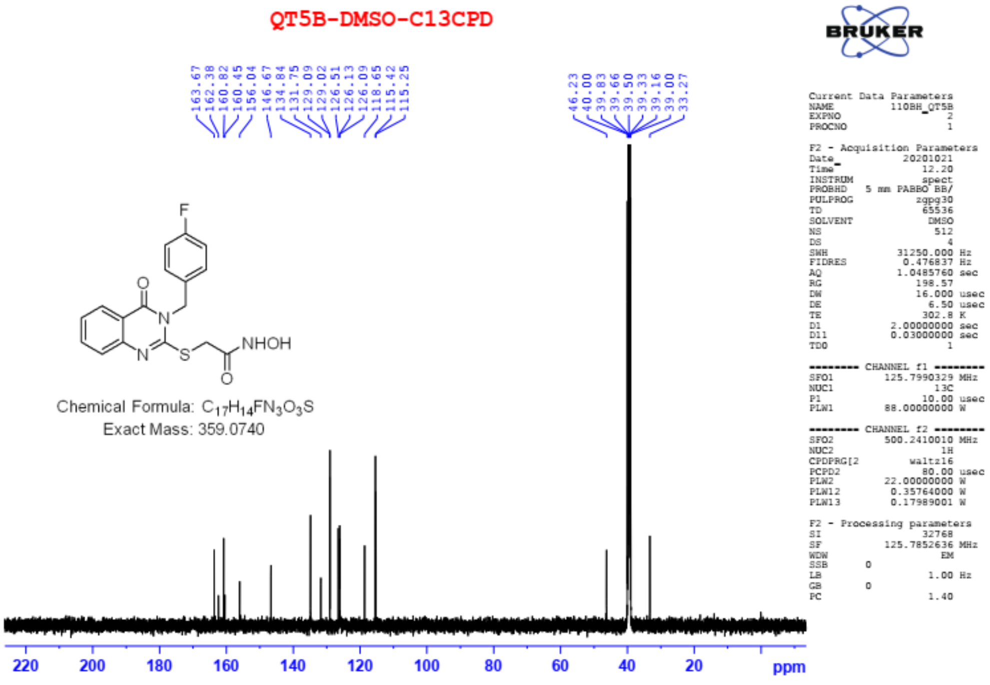

Figure S4.9 ^{13}C-NMR spectrum of compound **2**

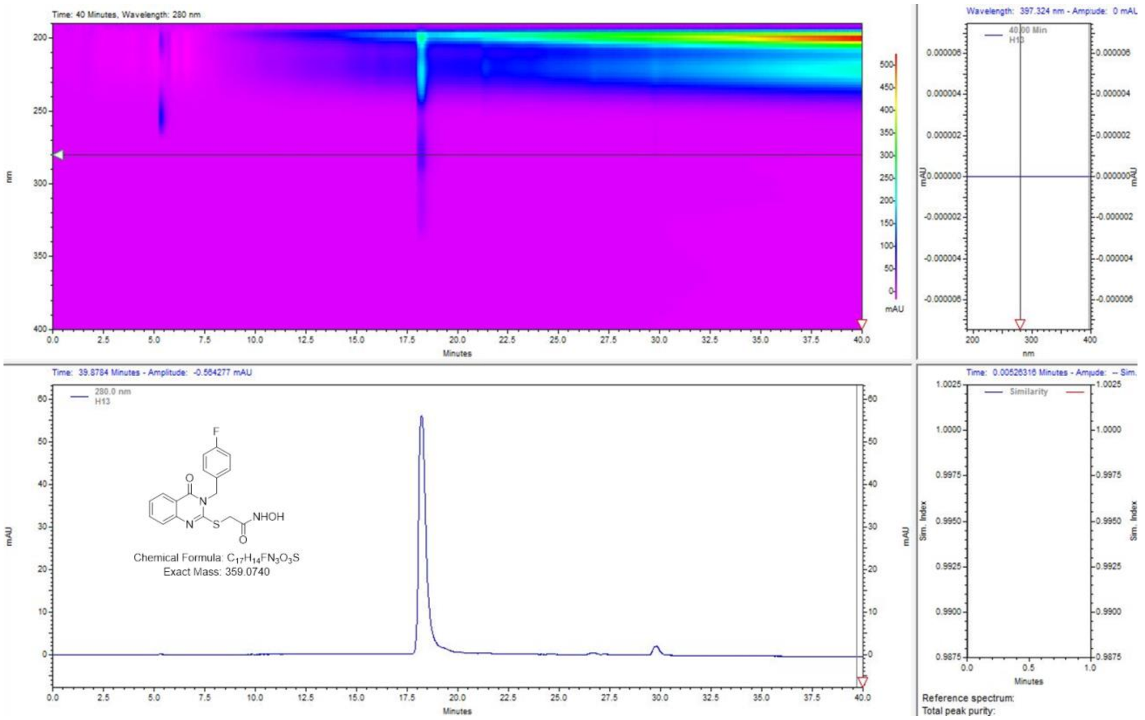

Figure S4.10 HPLC spectrum of compound **2**

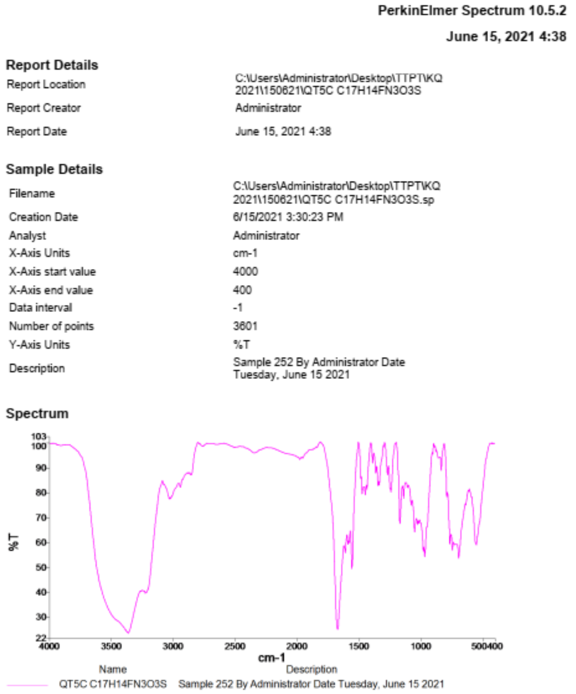

Figure S4.11 IR spectrum of compound **3**

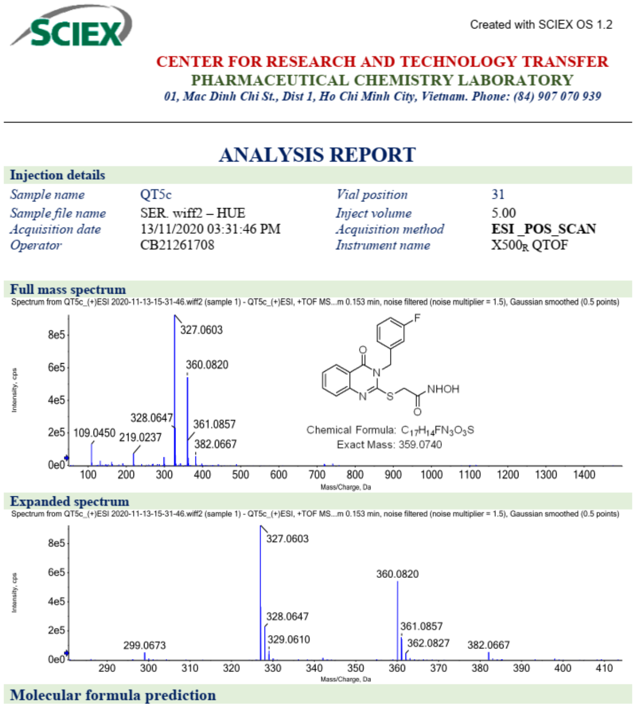

Figure S4.12 MS spectrum of compound **3**

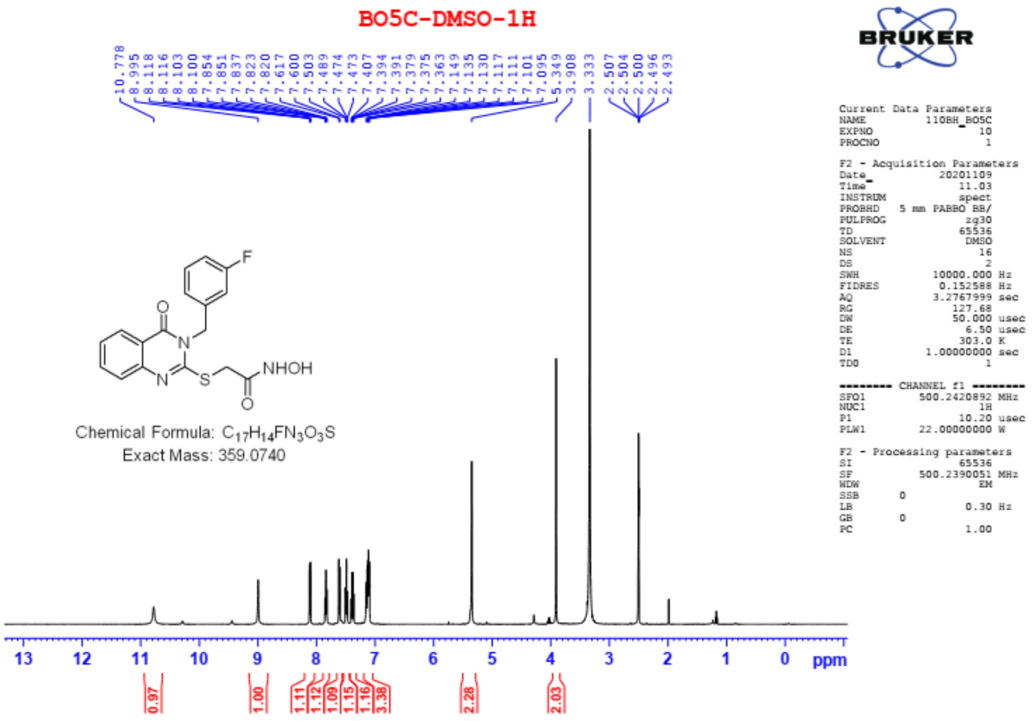

Figure S4.13 ^{1}H-NMR spectrum of compound **3**

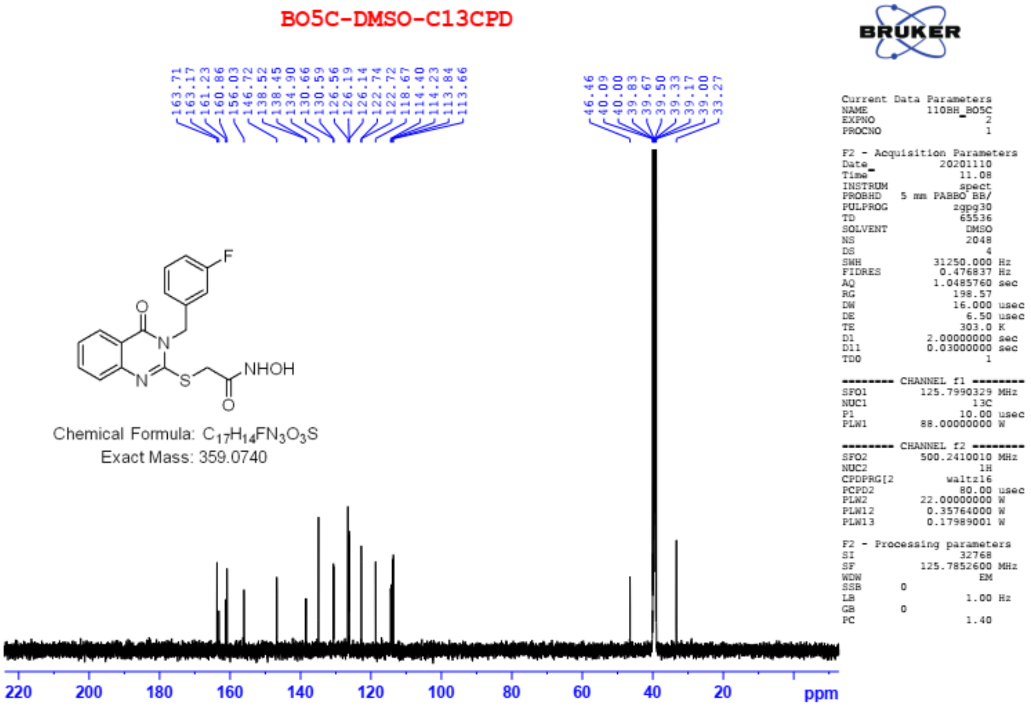

Figure S4.14 ^{13}C-NMR spectrum of compound **3**

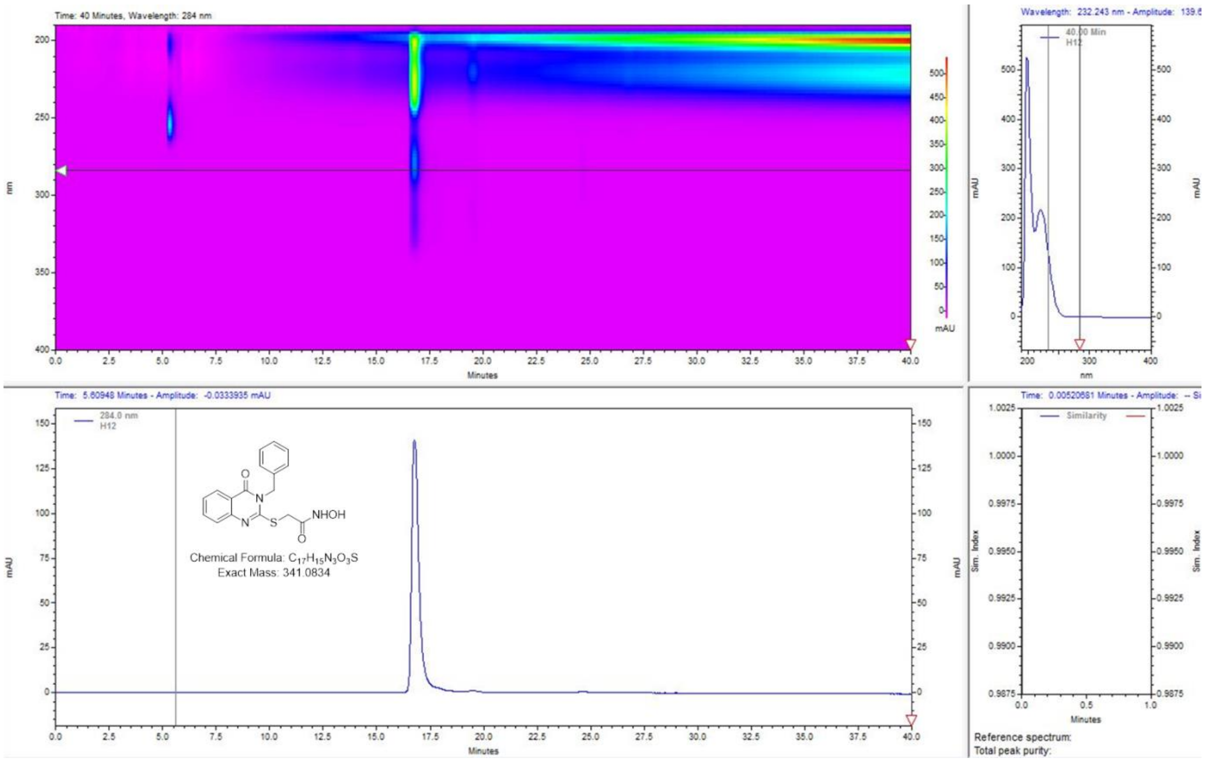

Figure S4.15 HPLC spectrum of compound **3**

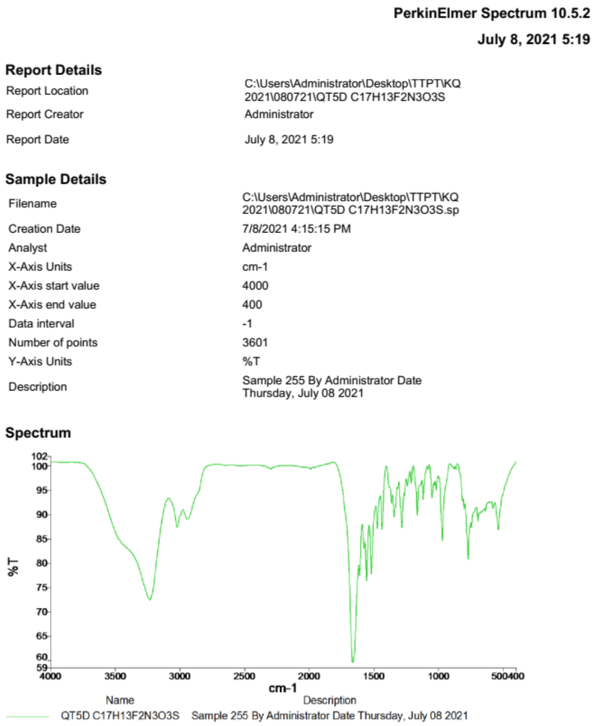

Figure S4.16 IR spectrum of compound **4**

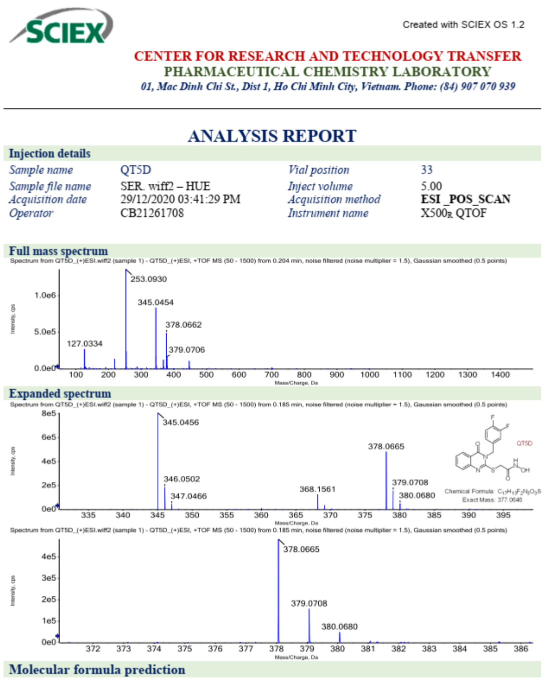

Figure S4.17 MS spectrum of compound **4**

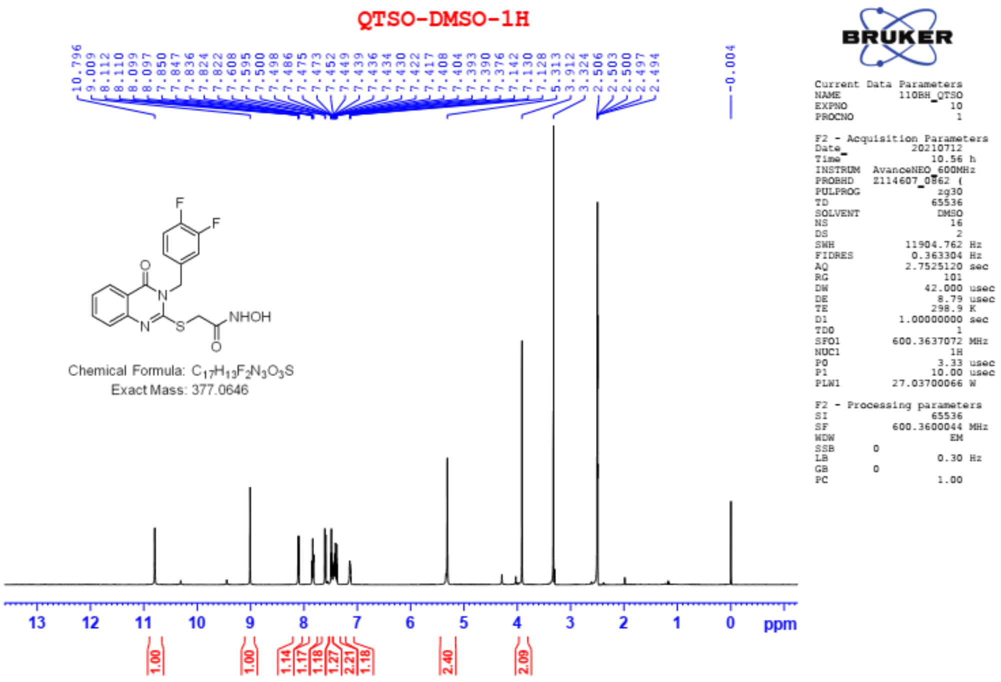

Figure S4.18 ^{1}H-NMR spectrum of compound **4**

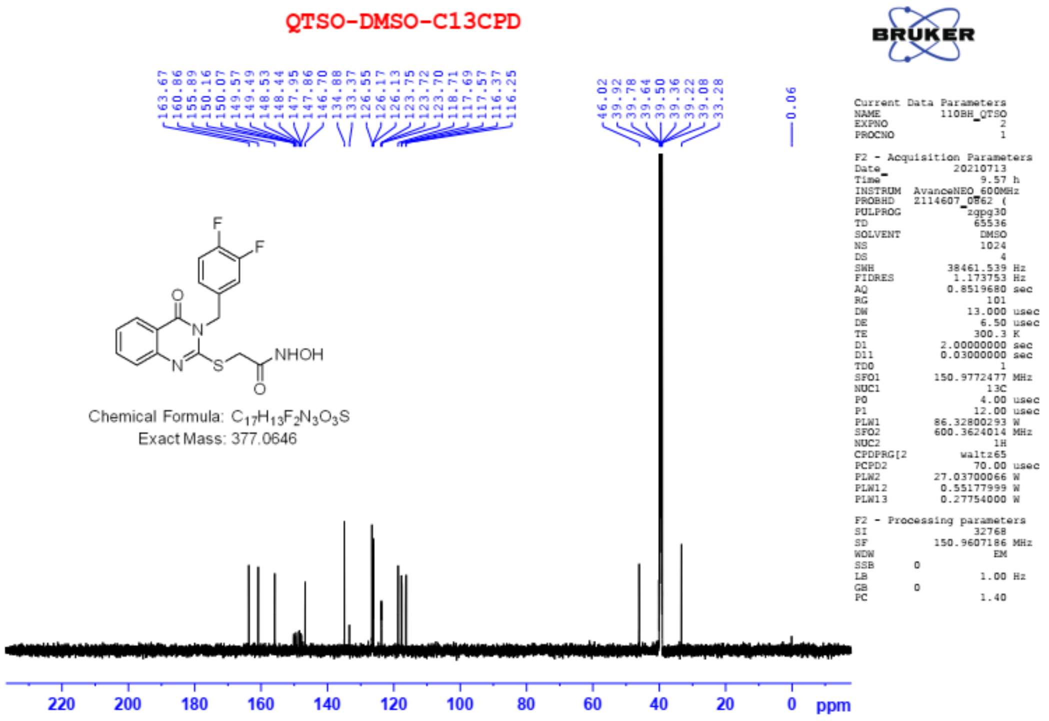

Figure S4.19 ^{13}C-NMR spectrum of compound **4**

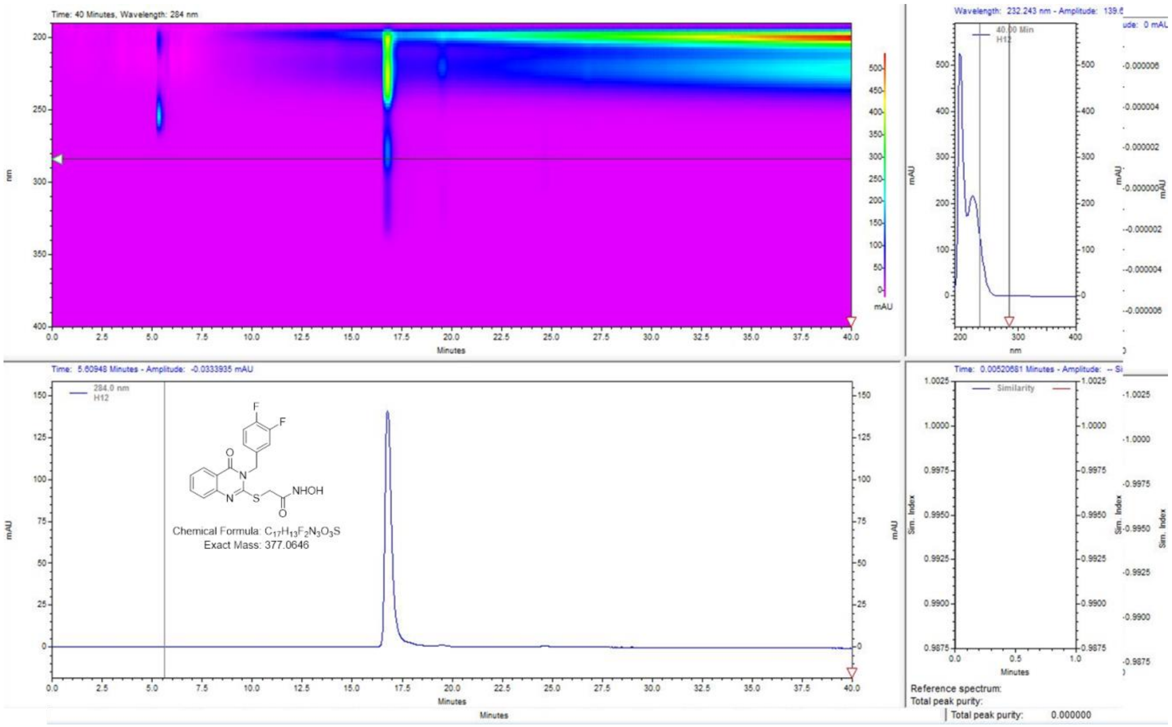

Figure S4.20 HPLC spectrum of compound **4**

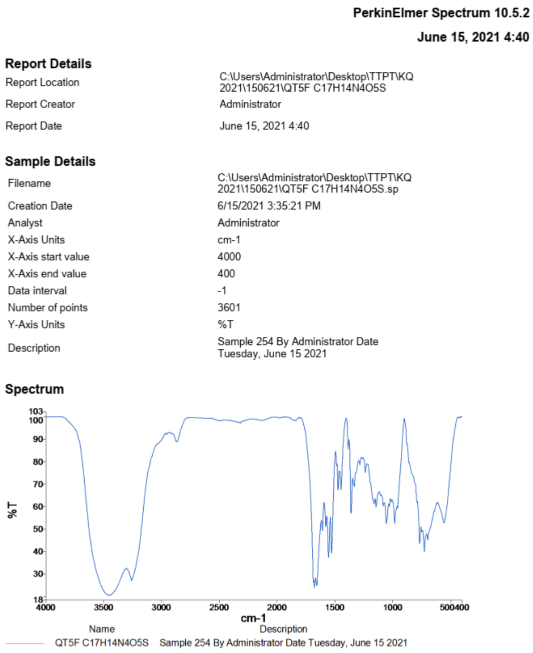

Figure S4.21 IR spectrum of compound **5**

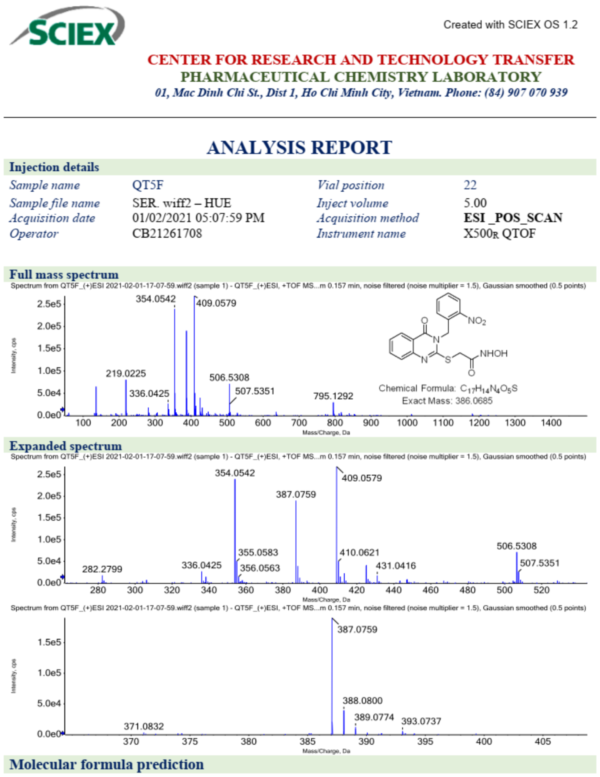

Figure S4.22 MS spectrum of compound **5**

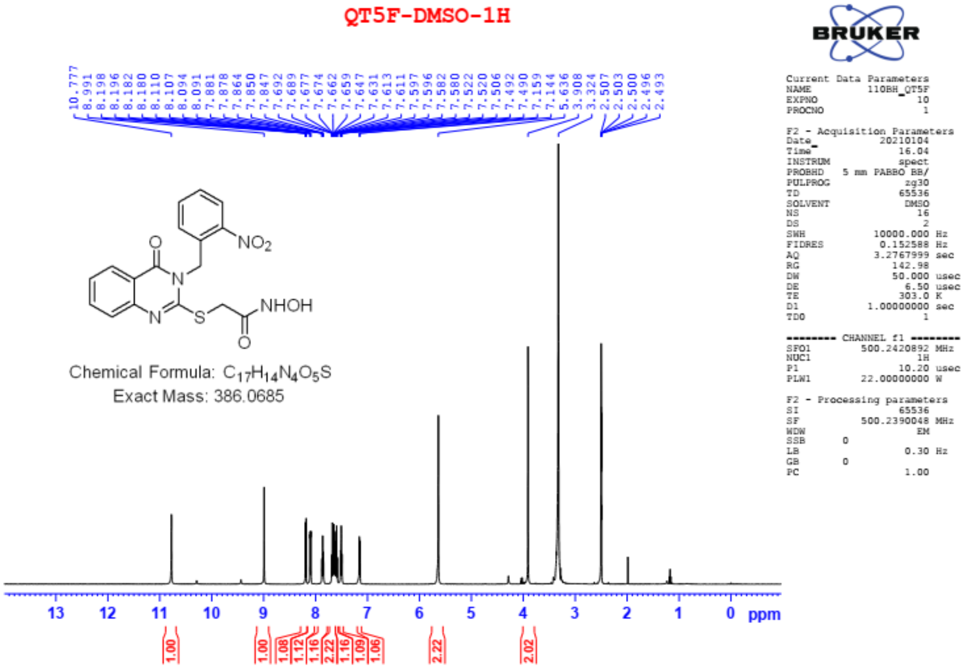

Figure S4.23 ^{1}H-NMR spectrum of compound **5**

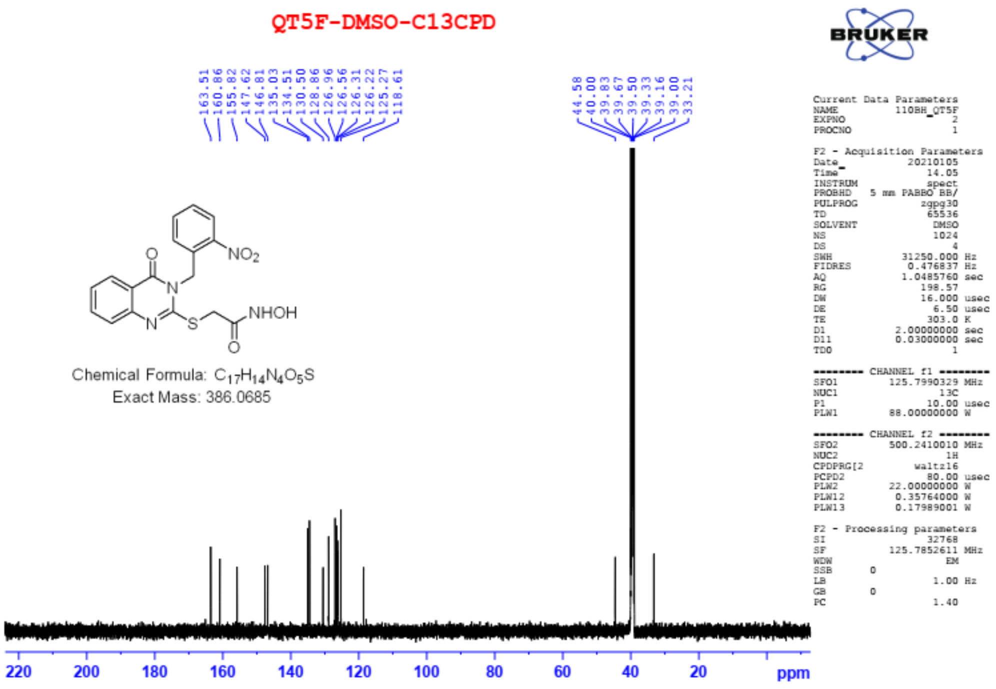

Figure S4.24 ^{13}C-NMR spectrum of compound **5**

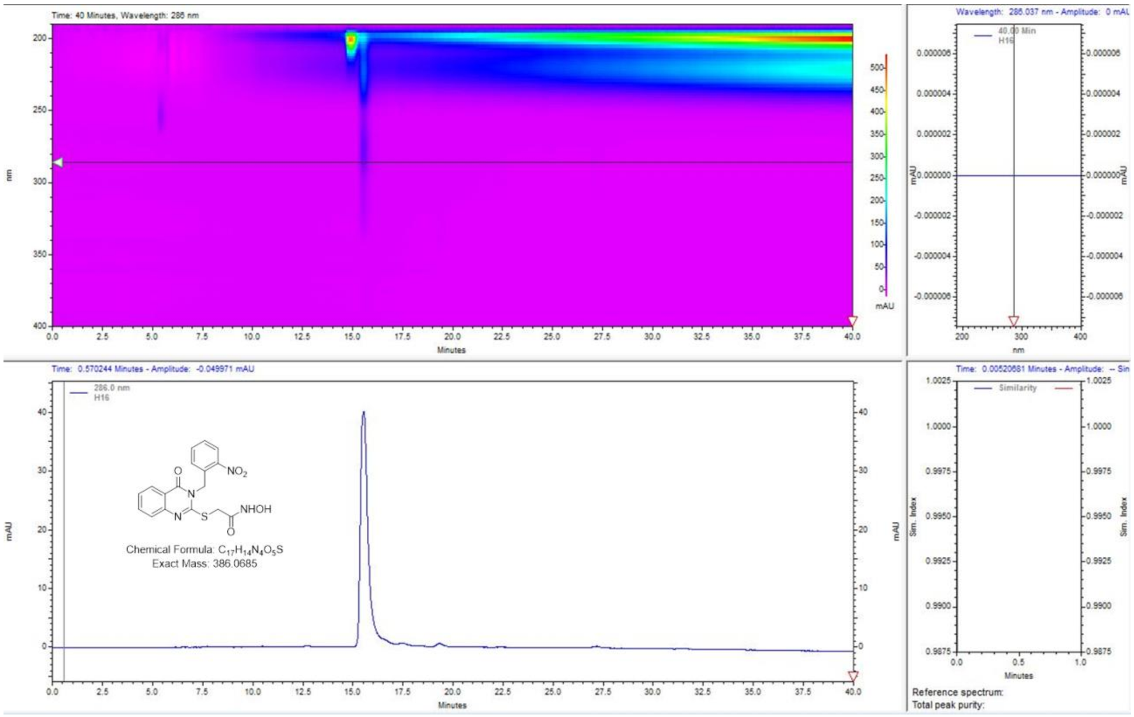

Figure S4.25 HPLC spectrum of compound **5**

PerkinElmer Spectrum 10.5.2

June 15, 2021 5:04

Report Details

Report Location	C:\Users\Administrator\Desktop\TTPT\KQ 2021\150621\QT5E C18H17N3O3S
Report Creator	Administrator
Report Date	June 15, 2021 5:04

Sample Details

Filename	C:\Users\Administrator\Desktop\TTPT\KQ 2021\150621\QT5E C18H17N3O3S.sp
Creation Date	6/15/2021 3:58:20 PM
Analyst	Administrator
X-Axis Units	cm-1
X-Axis start value	4000
X-Axis end value	400
Data interval	-1
Number of points	3601
Y-Axis Units	%T
Description	Sample 254 By Administrator Date Tuesday, June 15 2021

Spectrum

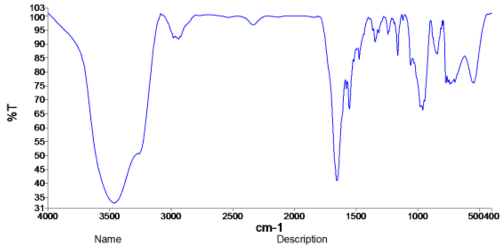

Name	Description
QT5E C18H17N3O3S	Sample 254 By Administrator Date Tuesday, June 15 2021

Figure S4.26 IR spectrum of compound **6**

Created with SCIEX OS 1.2

CENTER FOR RESEARCH AND TECHNOLOGY TRANSFER

PHARMACEUTICAL CHEMISTRY LABORATORY

01, Mac Dinh Chi St., Dist 1, Ho Chi Minh City, Vietnam. Phone: (84) 907 070 939

ANALYSIS REPORT

Injection details

Sample name	QT7E	*Vial position*	23
Sample file name	SER. wiff2 – HUE	*Inject volume*	5.00
Acquisition date	01/02/2021 05:11:34 PM	*Acquisition method*	**ESI _POS_SCAN**
Operator	CB21261708	*Instrument name*	X500R QTOF

Full mass spectrum

Spectrum from QT7E_(+)ESI 2021-02-01-17-11-34.wiff2 (sample 1) - QT7E_(+)ESI, +TOF MS...m 0.171 min, noise filtered (noise multiplier = 1.5), Gaussian smoothed (0.5 points)

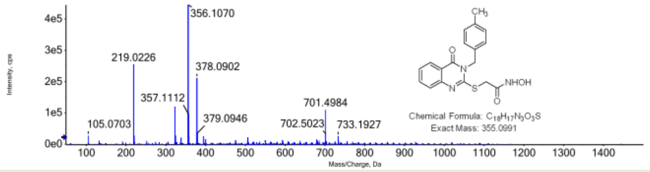

Expanded spectrum

Spectrum from QT7E_(+)ESI 2021-02-01-17-11-34.wiff2 (sample 1) - QT7E_(+)ESI, +TOF MS...m 0.171 min, noise filtered (noise multiplier = 1.5), Gaussian smoothed (0.5 points)

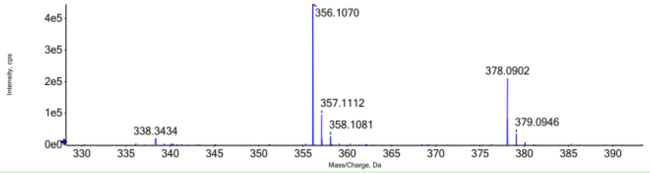

Molecular formula prediction

Figure S4.27 MS spectrum of compound **6**

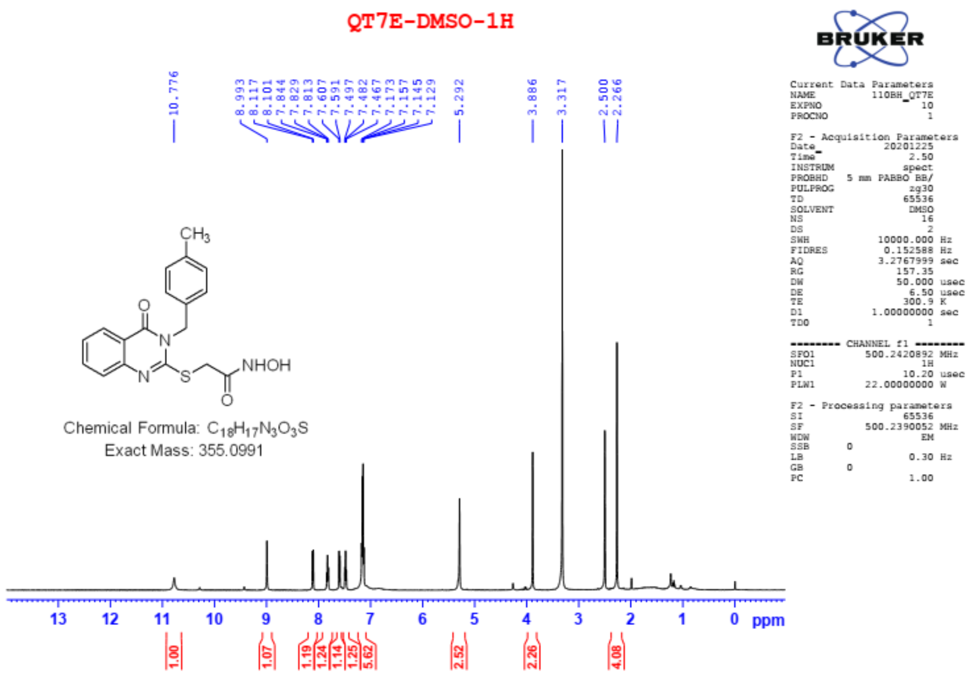

Figure S4.28 ^{1}H-NMR spectrum of compound **6**

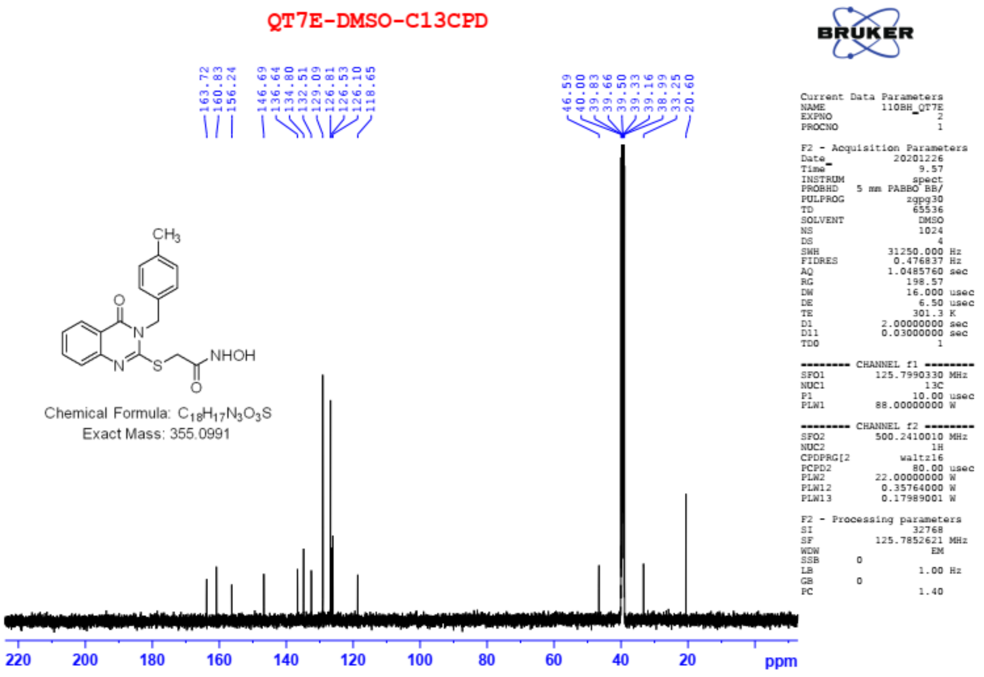

Figure S4.29 ^{13}C-NMR spectrum of compound **6**

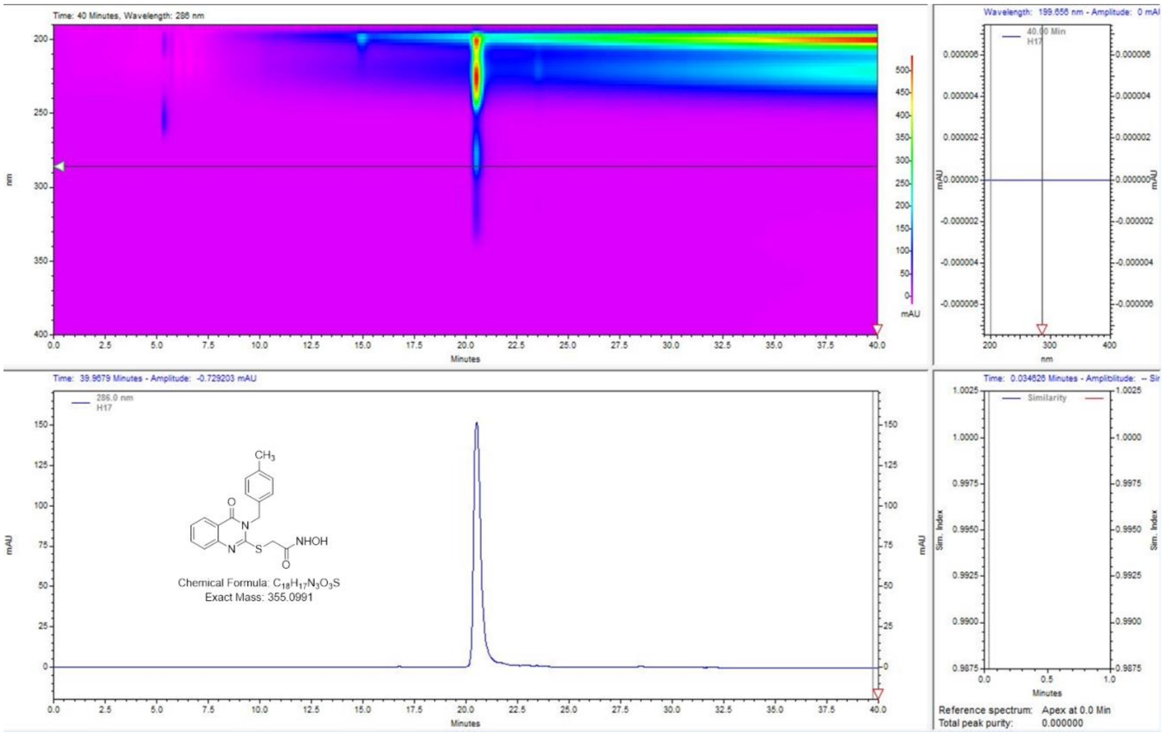

Figure S4.30 HPLC spectrum of compound **6**

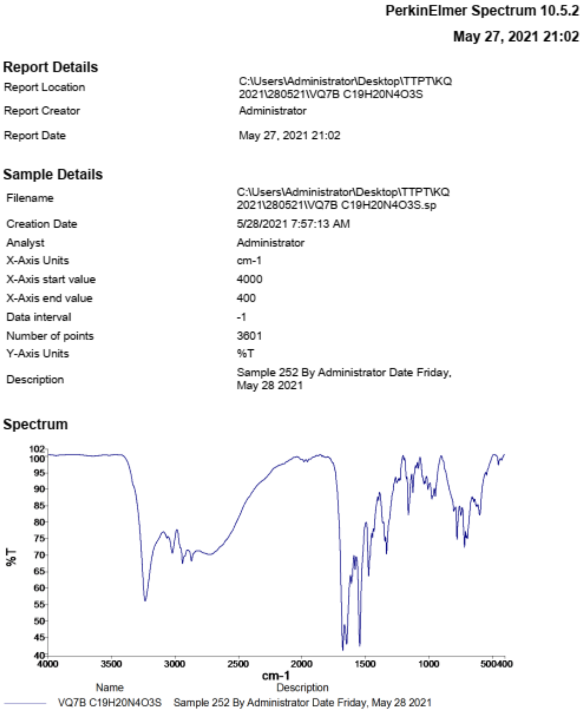

Figure S4.31 IR spectrum of compound **7**

Created with SCIEX OS 1.2

CENTER FOR RESEARCH AND TECHNOLOGY TRANSFER
PHARMACEUTICAL CHEMISTRY LABORATORY
1B, Thanh Loc 29 St., Dist 12, Ho Chi Minh City, Vietnam. Phone: (84) 907 070 939

ANALYSIS REPORT

Injection details

Sample name	VQ7B	*Vial position*	30
Sample file name	SER. wiff2 – HUE	*Inject volume*	5.00
Acquisition date	09/06/2021 17:25:54 PM	*Acquisition method*	**ESI _POS_SCAN**
Operator	CB21261708	*Instrument name*	X500R QTOF

Full mass spectrum

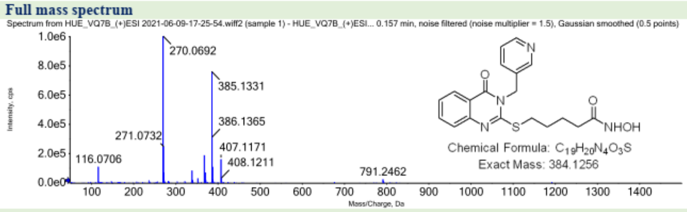

Expanded spectrum

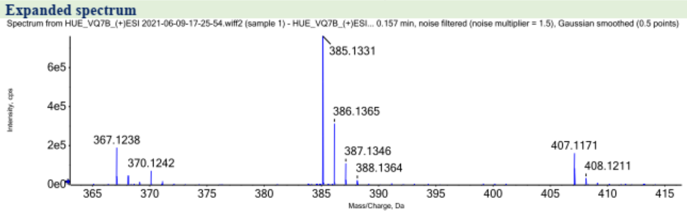

Figure S4.32 MS spectrum of compound **7**

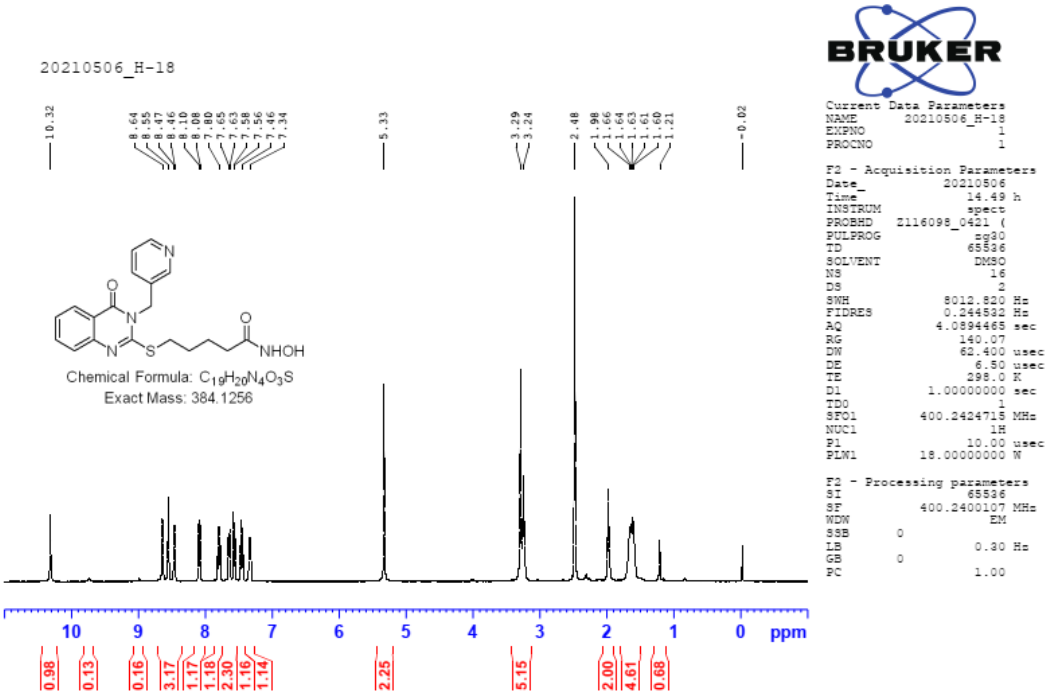

Figure S4.33 ^{1}H-NMR spectrum of compound **7**

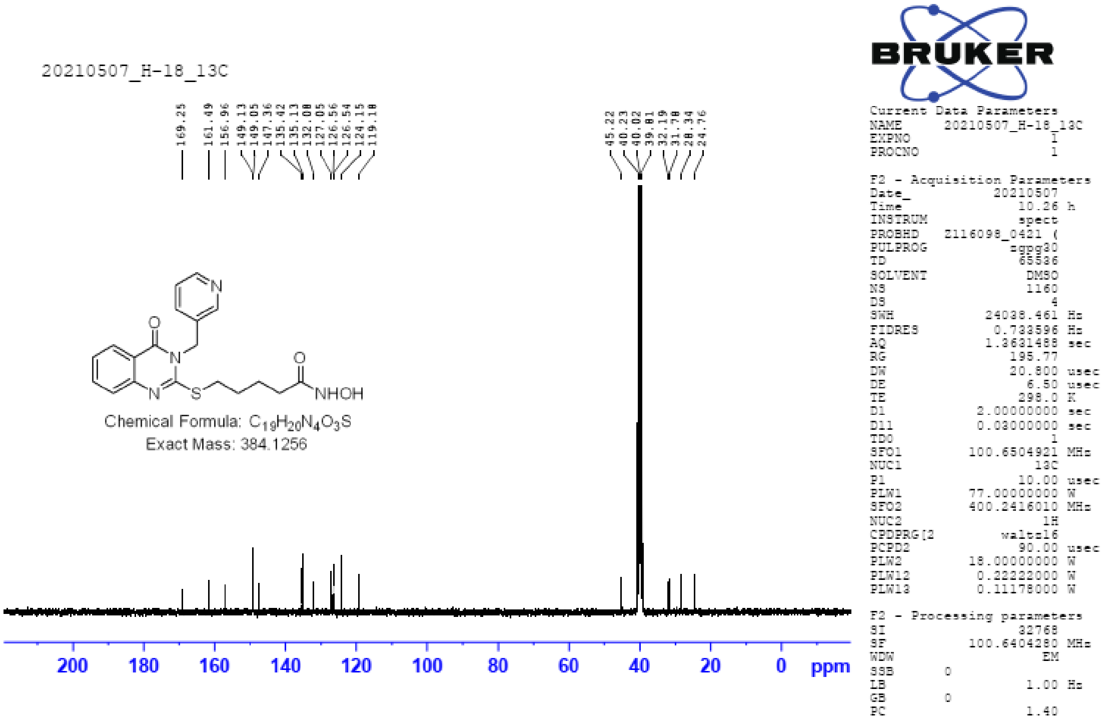

Figure S4.34 ^{13}C-NMR spectrum of compound **7**

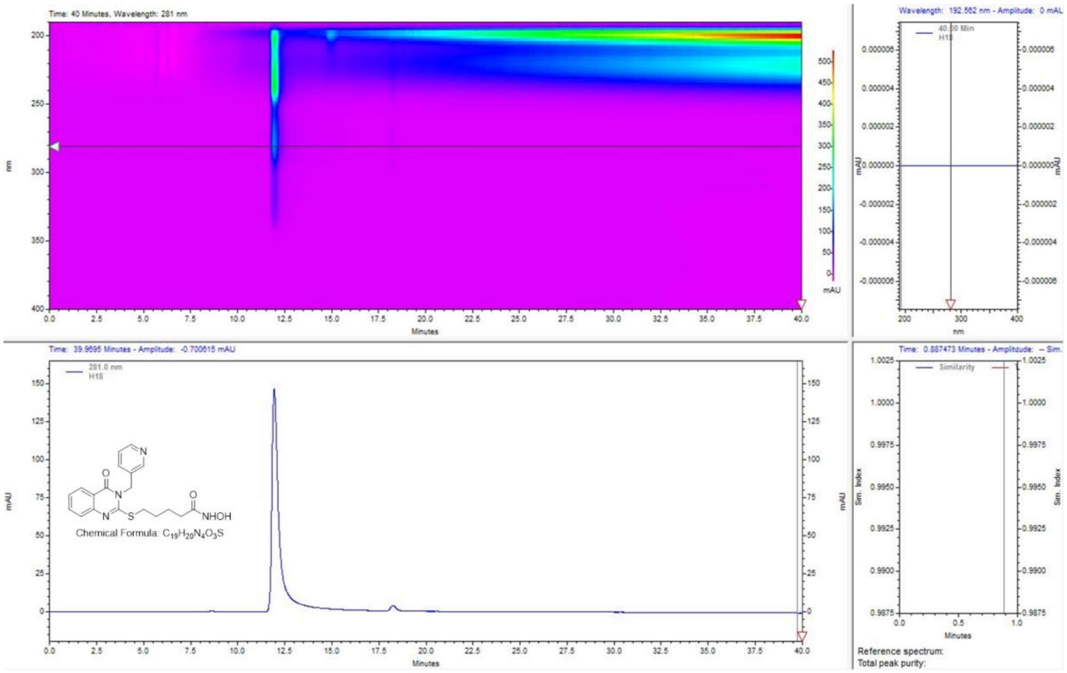

Figure S4.35 HPLC spectrum of compound **7**

PerkinElmer Spectrum 10.5.2

May 27, 2021 20:51

Report Details

Report Location	C:\Users\Administrator\Desktop\TTPT\KQ 2021\280521\VQ7A C20H20FN3O3S
Report Creator	Administrator
Report Date	May 27, 2021 20:51

Sample Details

Filename	C:\Users\Administrator\Desktop\TTPT\KQ 2021\280521\VQ7A C20H20FN3O3S.sp
Creation Date	5/28/2021 7:47:32 AM
Analyst	Administrator
X-Axis Units	cm-1
X-Axis start value	4000
X-Axis end value	400
Data interval	-1
Number of points	3601
Y-Axis Units	%T
Description	Sample 252 By Administrator Date Friday, May 28 2021

Spectrum

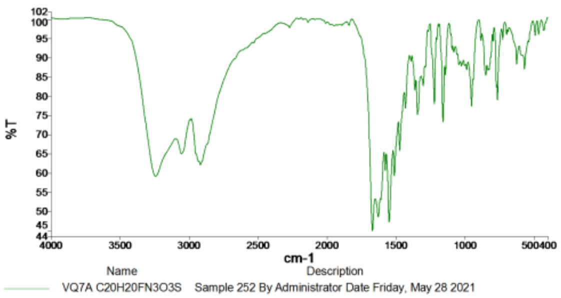

Figure S4.36 IR spectrum of compound **8**

Created with SCIEX OS 1.2

CENTER FOR RESEARCH AND TECHNOLOGY TRANSFER
PHARMACEUTICAL CHEMISTRY LABORATORY
1B, Thanh Loc 29 St., Dist 12, Ho Chi Minh City, Vietnam. Phone: (84) 907 070 939

ANALYSIS REPORT

Injection details

Sample name	VQ7A	*Vial position*	28
Sample file name	SER. wiff2 – HUE	*Inject volume*	5.00
Acquisition date	21/05/2021 15:32:18 PM	*Acquisition method*	**ESI _POS_SCAN**
Operator	CB21261708	*Instrument name*	$X500_R$ QTOF

Full mass spectrum

Spectrum from HUE_VQ7A_(+)ESI 2021-05-21-15-32-18.wiff2 (sample 1) - HUE_VQ7A_(+)ESI... 0.213 min, noise filtered (noise multiplier = 1.5), Gaussian smoothed (0.5 points)

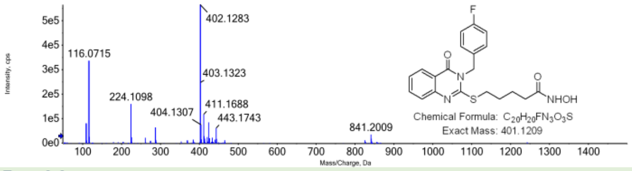

Expanded spectrum

Spectrum from HUE_VQ7A_(+)ESI 2021-05-21-15-32-18.wiff2 (sample 1) - HUE_VQ7A_(+)ESI... 0.213 min, noise filtered (noise multiplier = 1.5), Gaussian smoothed (0.5 points)

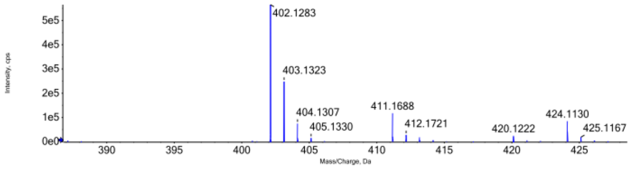

Figure S4.37 MS spectrum of compound **8**

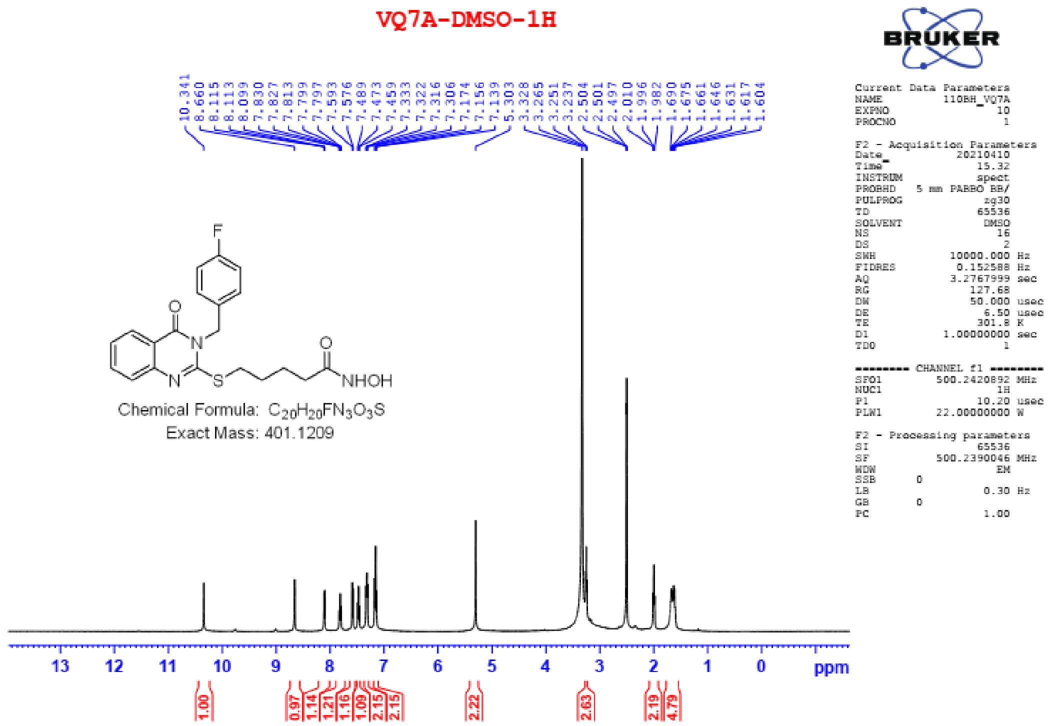

Figure S4.38 ^{1}H-NMR spectrum of compound **8**

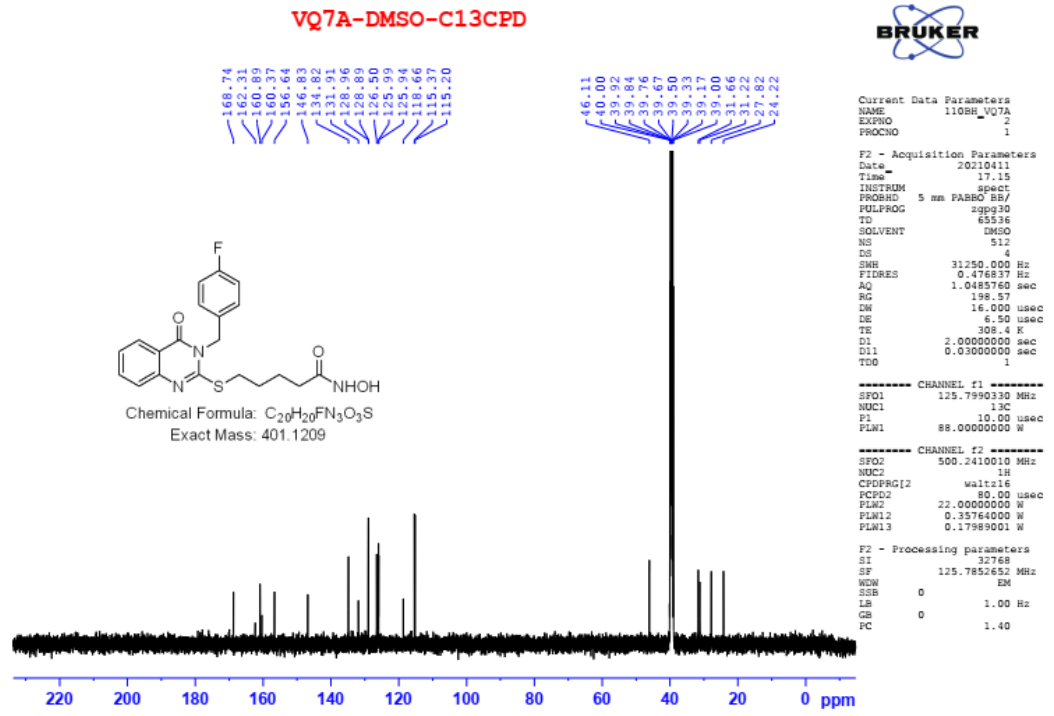

Figure S4.39 ^{13}C-NMR spectrum of compound **8**

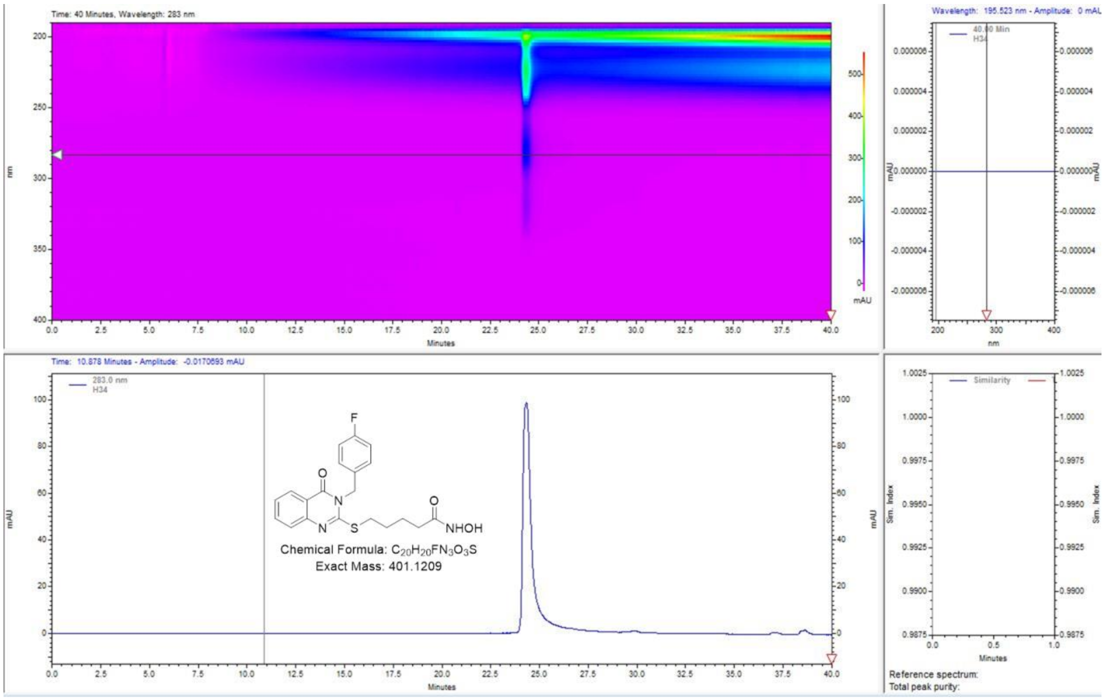

Figure S4.40 HPLC spectrum of compound **8**

PerkinElmer Spectrum 10.5.2

May 27, 2021 20:56

Report Details

Report Location	C:\Users\Administrator\Desktop\TTPT\KQ 2021\280521\VQ7C C20H19F2N3O3S
Report Creator	Administrator
Report Date	May 27, 2021 20:56

Sample Details

Filename	C:\Users\Administrator\Desktop\TTPT\KQ 2021\280521\VQ7C C20H19F2N3O3S.sp
Creation Date	5/28/2021 7:52:20 AM
Analyst	Administrator
X-Axis Units	cm-1
X-Axis start value	4000
X-Axis end value	400
Data interval	-1
Number of points	3601
Y-Axis Units	%T
Description	Sample 252 By Administrator Date Friday, May 28 2021

Spectrum

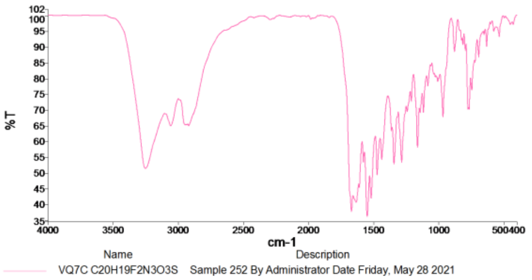

Figure S4.41 IR spectrum of compound **9**

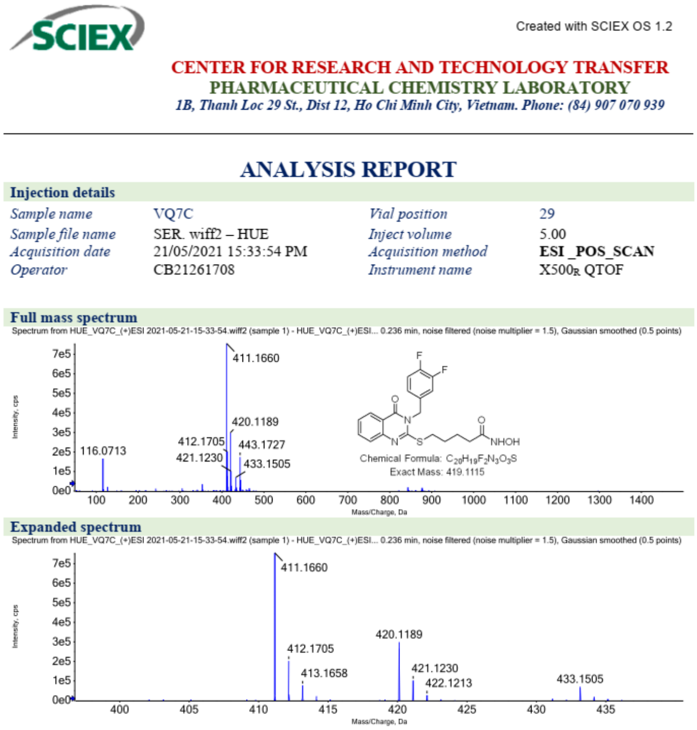

Figure S4.42 MS spectrum of compound **9**

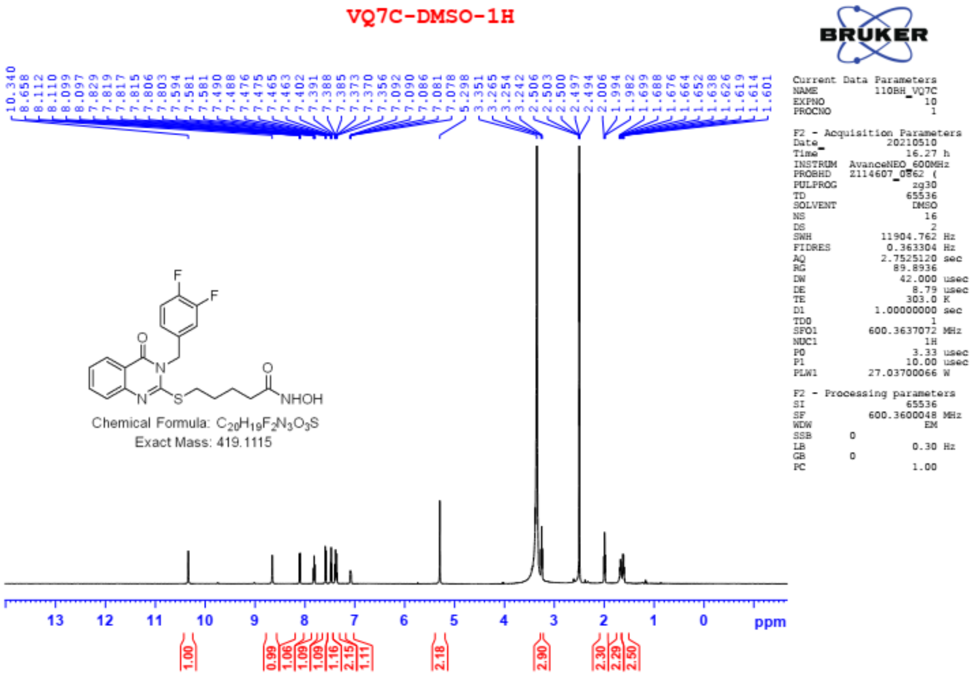

Figure S4.43 ^{1}H-NMR spectrum of compound **9**

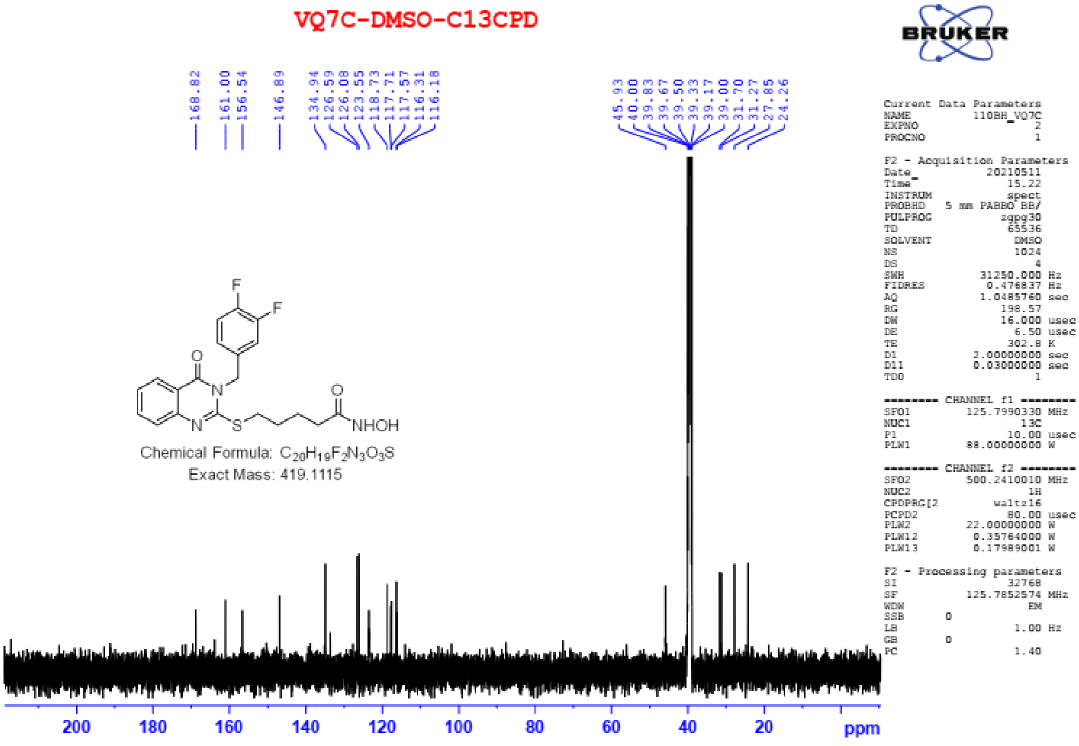

Figure S4.44 ^{13}C-NMR spectrum of compound **9**

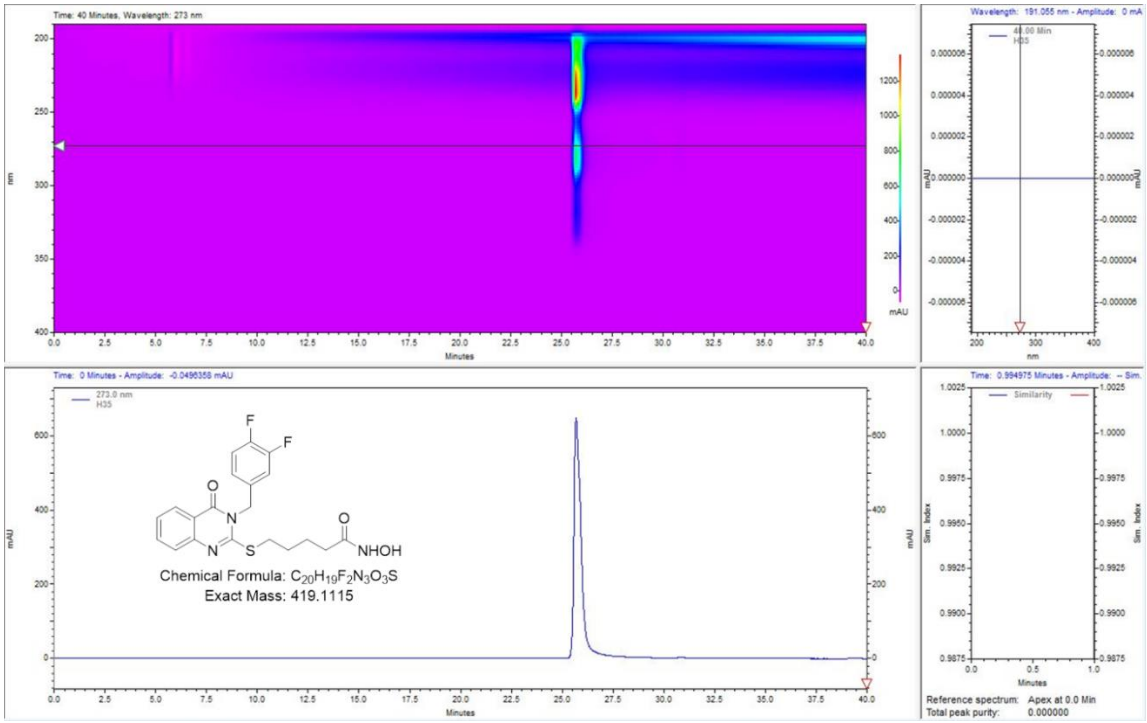

Figure S4.45 HPLC spectrum of compound **9**

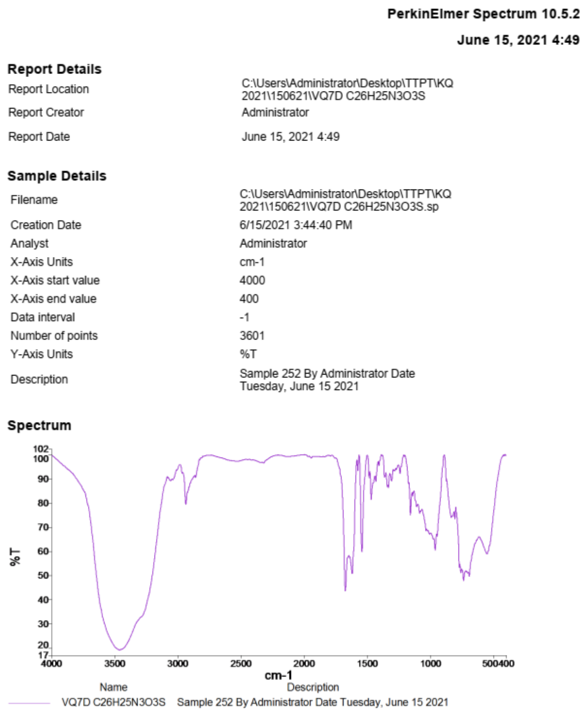

Figure S4.46 IR spectrum of compound **10**

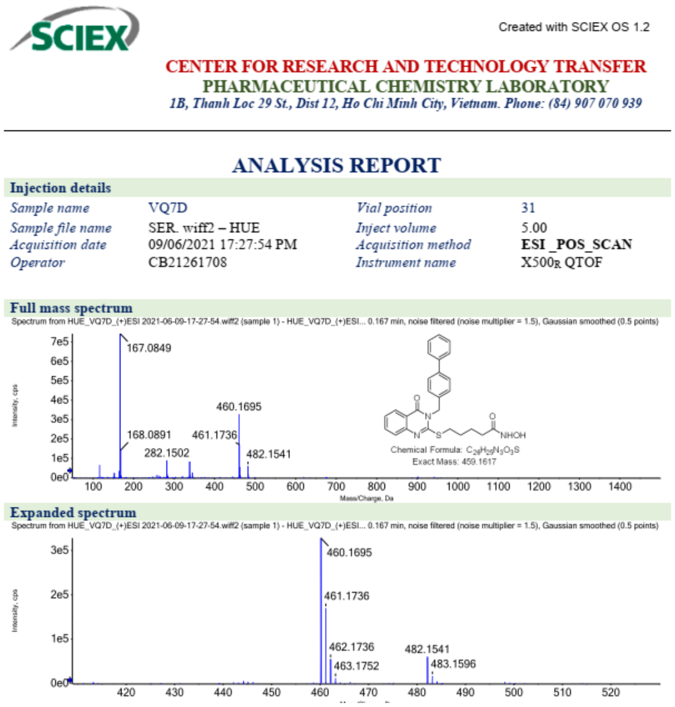

Figure S4.47 MS spectrum of compound **10**

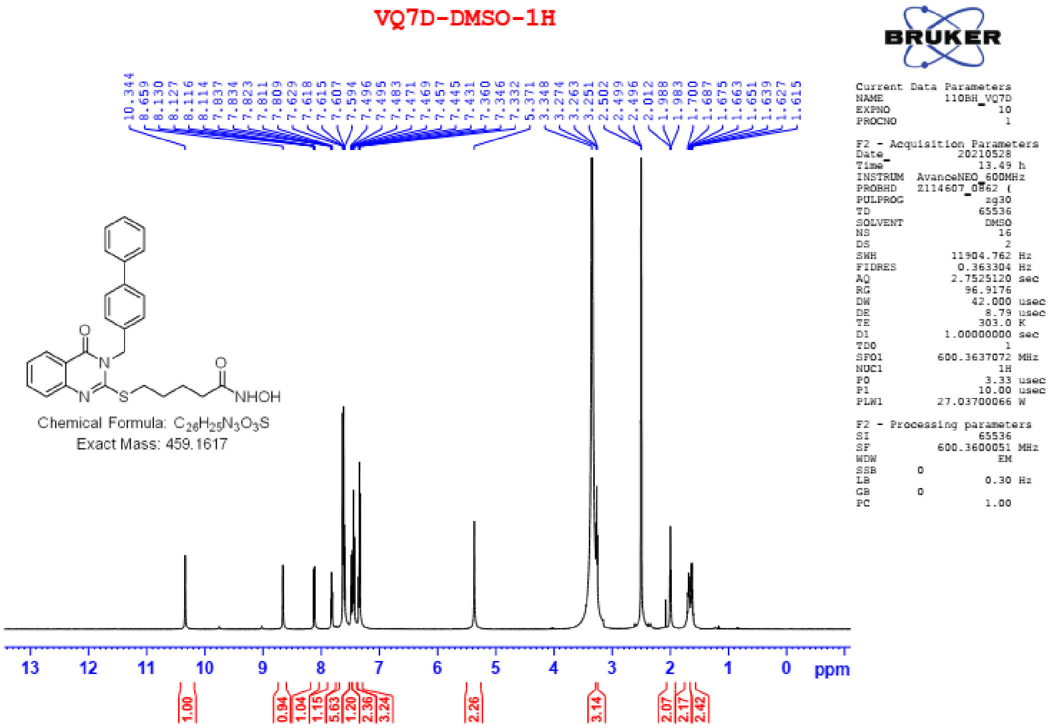

Figure S4.48 ^{1}H-NMR spectrum of compound **10**

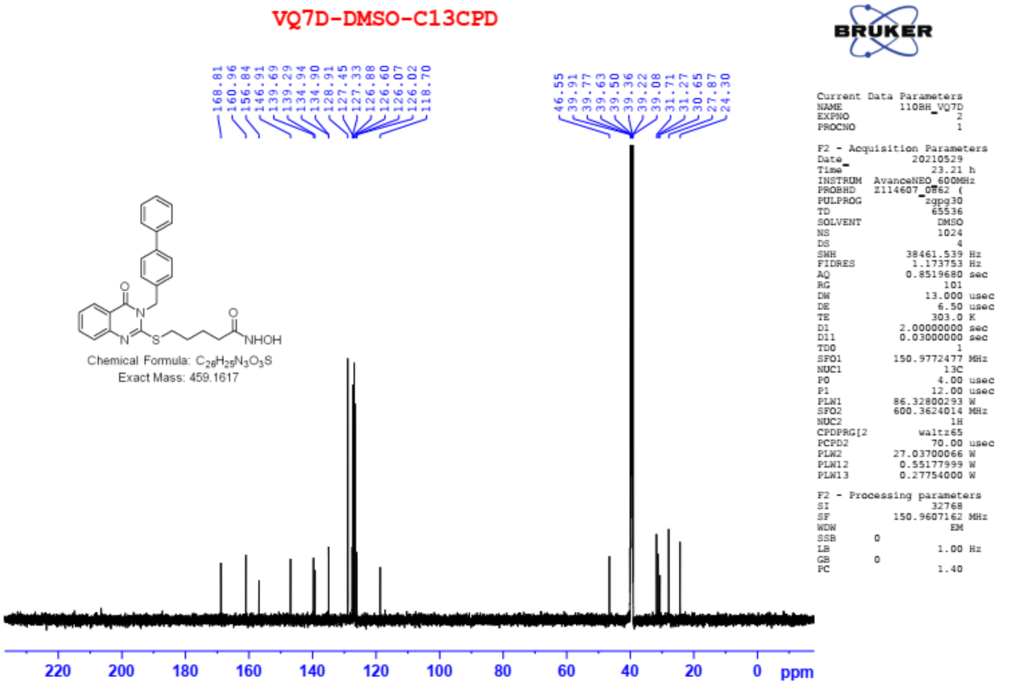

Figure S4.49 ^{13}C-NMR spectrum of compound **10**

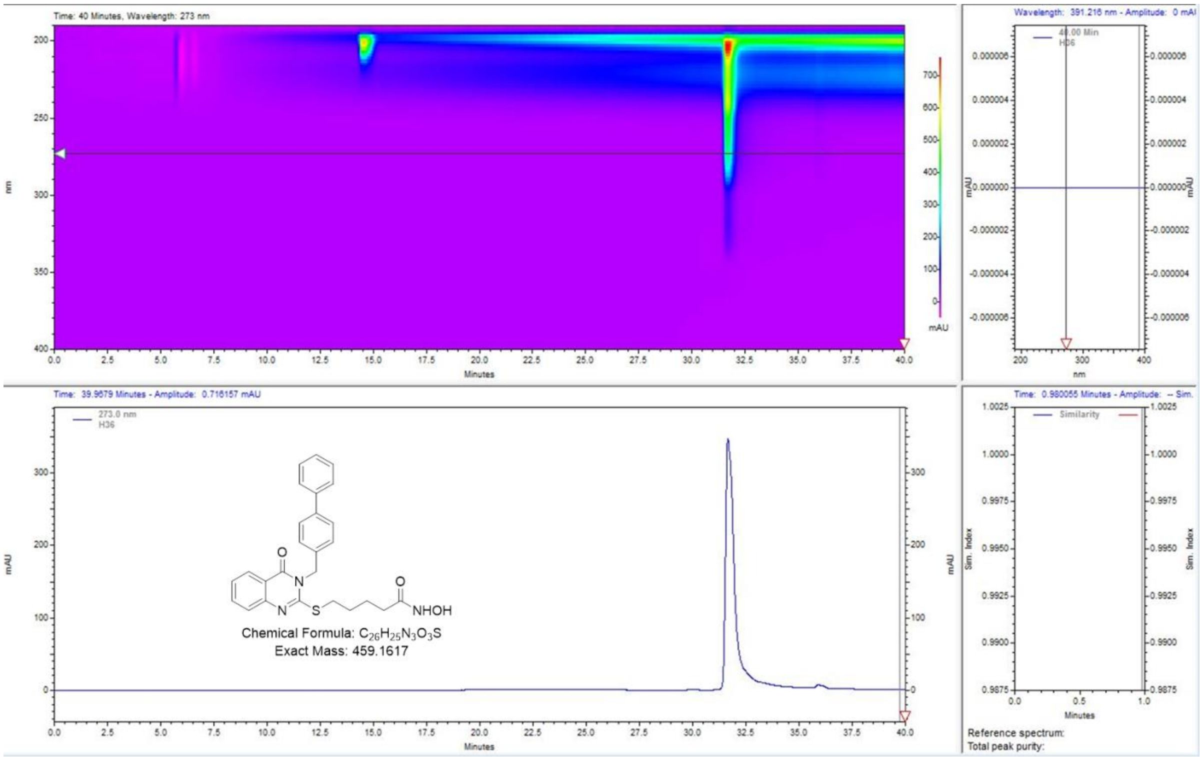

Figure S4.50 HPLC spectrum of compound **10**

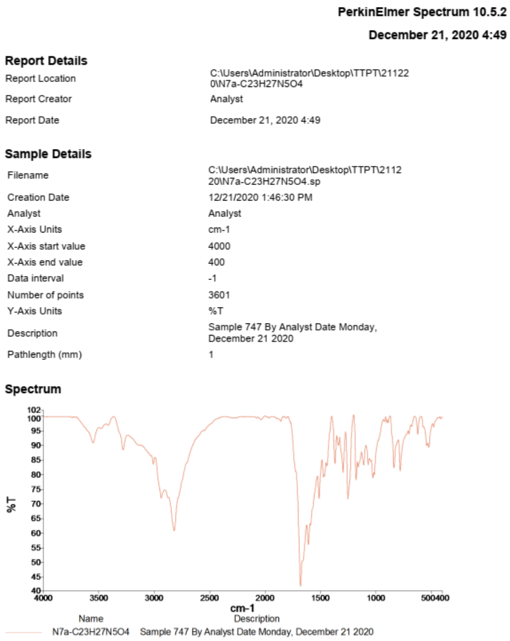

Figure S4.51 IR spectrum of compound **11**

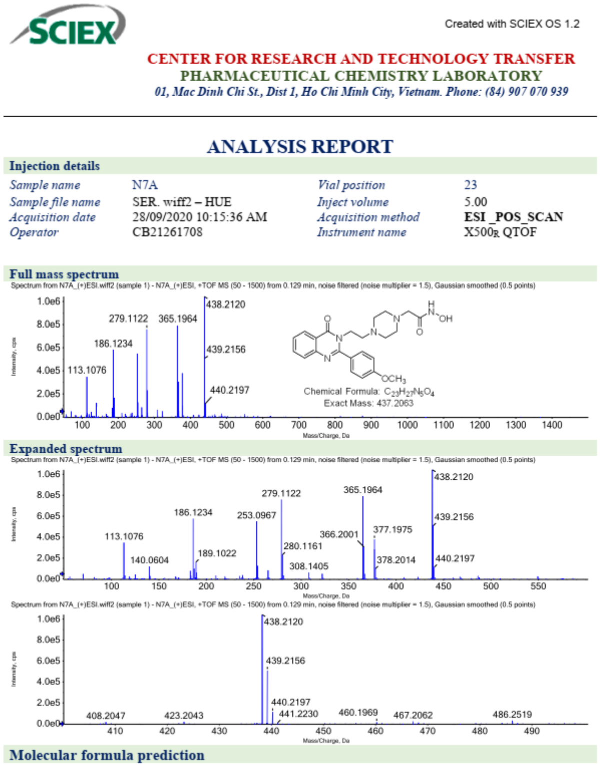

Figure S4.52 MS spectrum of compound **11**

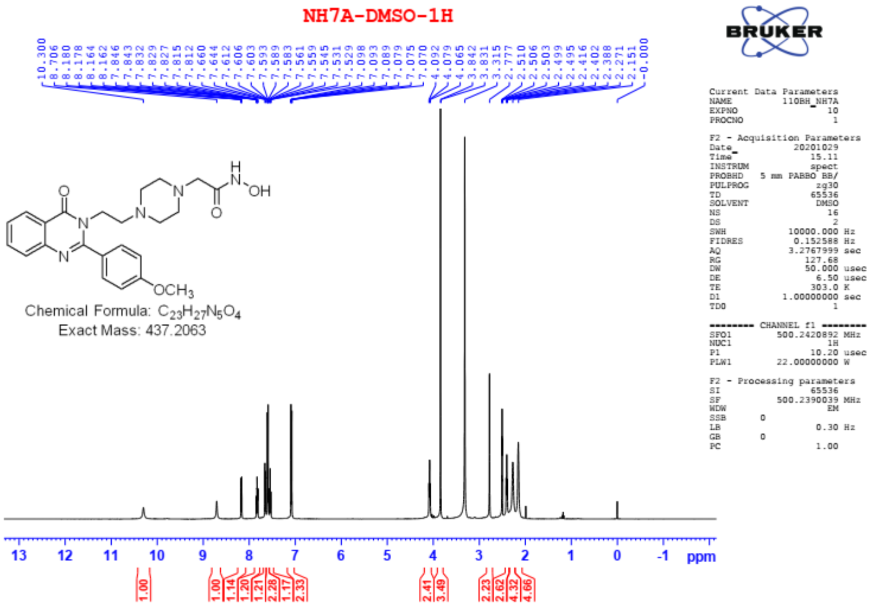

Figure S4.53 ^{1}H-NMR spectrum of compound **11**

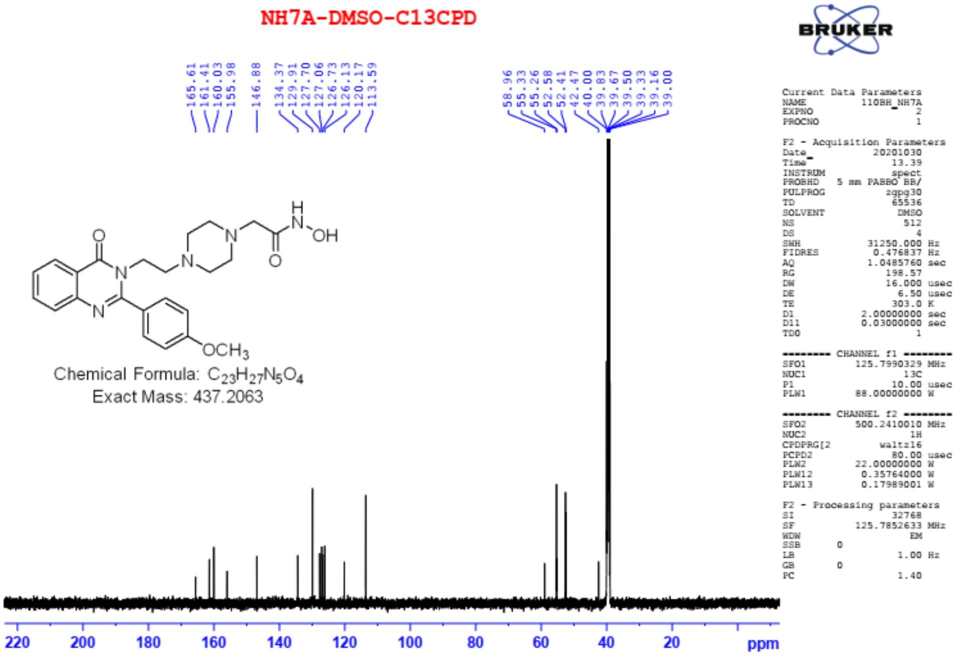

Figure S4.54 ^{13}C-NMR spectrum of compound **11**

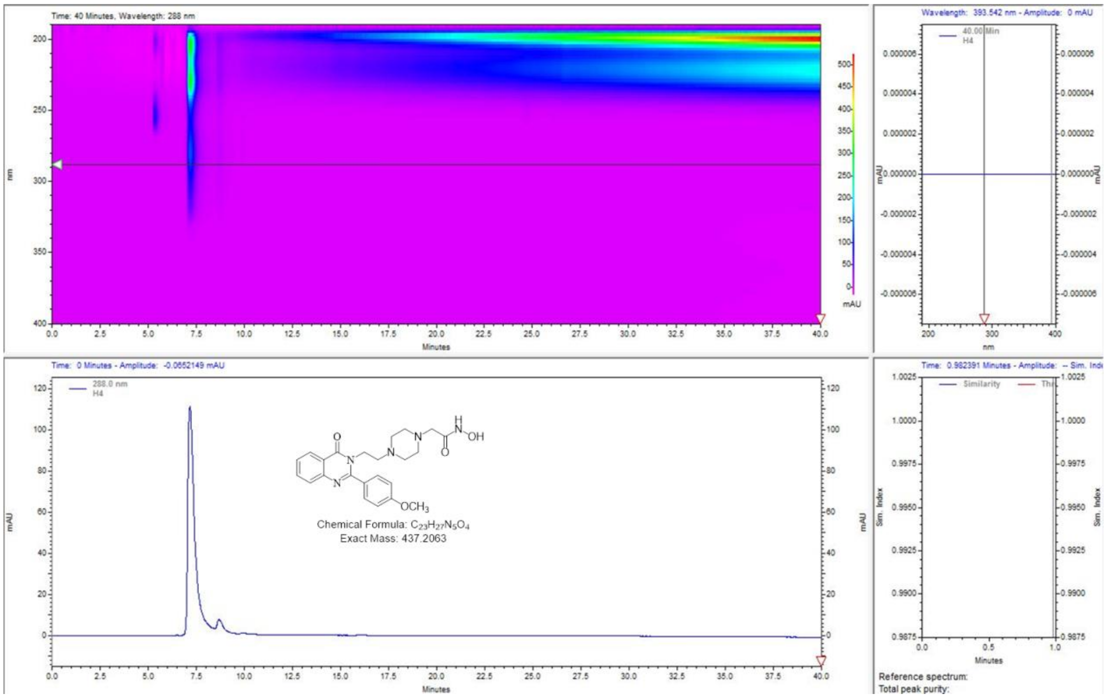

Figure S4.55 HPLC spectrum of compound **11**

PerkinElmer Spectrum 10.5.2

December 21, 2020 4:51

Report Details

Report Location	C:\Users\Administrator\Desktop\TTPT\21122 0\N7b-C22H24FN5O3
Report Creator	Analyst
Report Date	December 21, 2020 4:51

Sample Details

Filename	C:\Users\Administrator\Desktop\TTPT\2112 20\N7b-C22H24FN5O3.sp
Creation Date	12/21/2020 1:50:44 PM
Analyst	Analyst
X-Axis Units	cm-1
X-Axis start value	4000
X-Axis end value	400
Data Interval	-1
Number of points	3601
Y-Axis Units	%T
Description	Sample 748 By Analyst Date Monday, December 21 2020
Pathlength (mm)	1

Spectrum

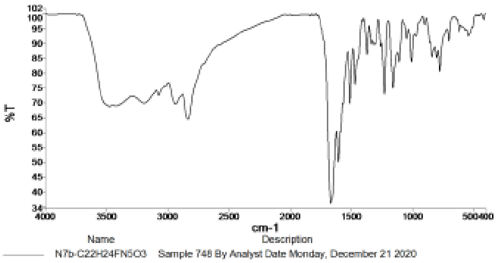

Figure S4.56 IR spectrum of compound **12**

SCIEX

Created with SCIEX OS 1.2

CENTER FOR RESEARCH AND TECHNOLOGY TRANSFER
PHARMACEUTICAL CHEMISTRY LABORATORY
01, Mac Dinh Chi St., Dist 1, Ho Chi Minh City, Vietnam. Phone: (84) 907 070 939

ANALYSIS REPORT

Injection details

Sample name	N7B	*Vial position*	34
Sample file name	SER. wiff2 – HUE	*Inject volume*	5.00
Acquisition date	14/10/2020 15:25:21 PM	*Acquisition method*	**ESI _POS_SCAN**
Operator	CB21261708	*Instrument name*	X500$_R$ QTOF

Full mass spectrum

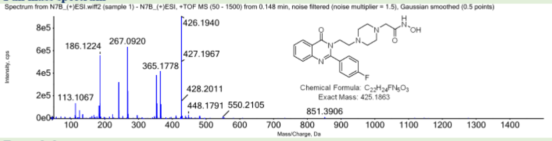

Expanded spectrum

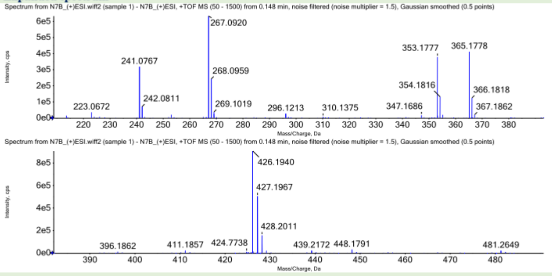

Molecular formula prediction

Figure S4.57 MS spectrum of compound **12**

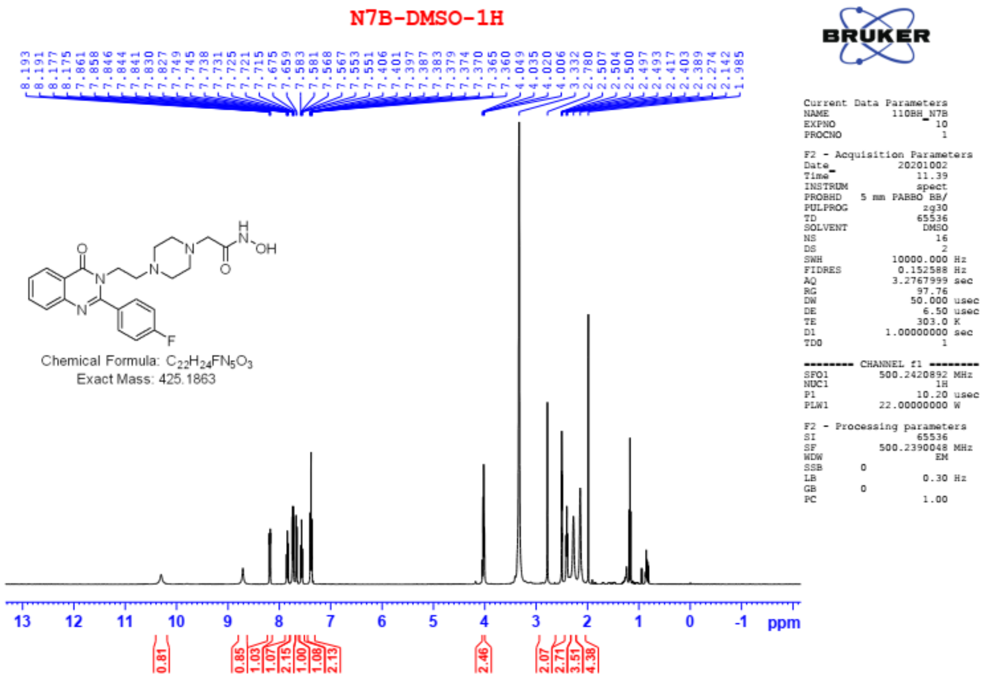

Figure S4.58 ^{1}H-NMR spectrum of compound **12**

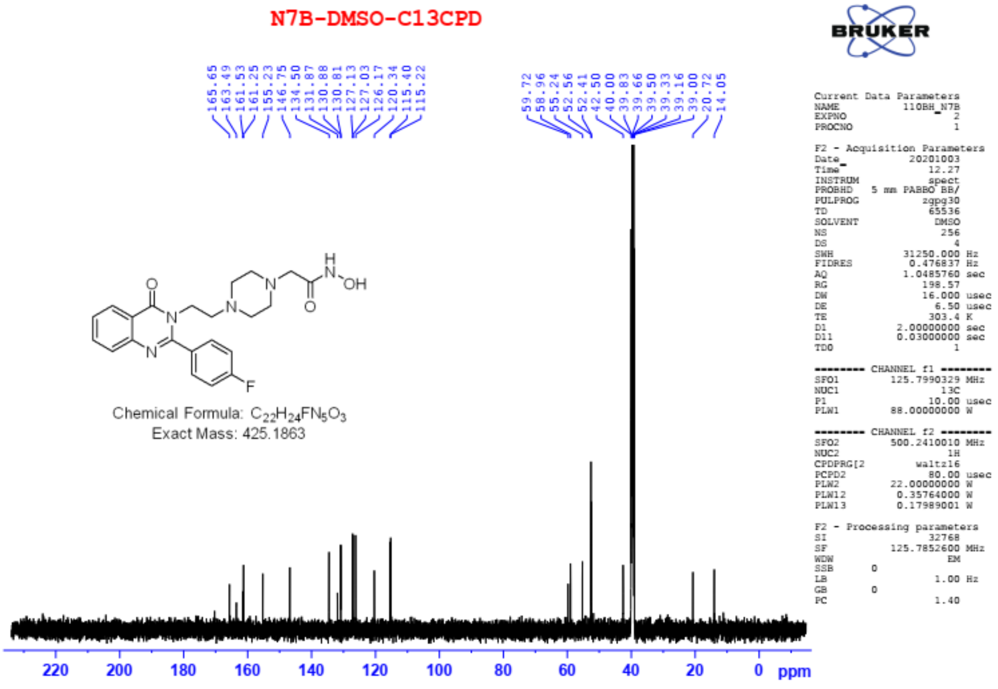

Figure S4.59 ^{13}C-NMR spectrum of compound **12**

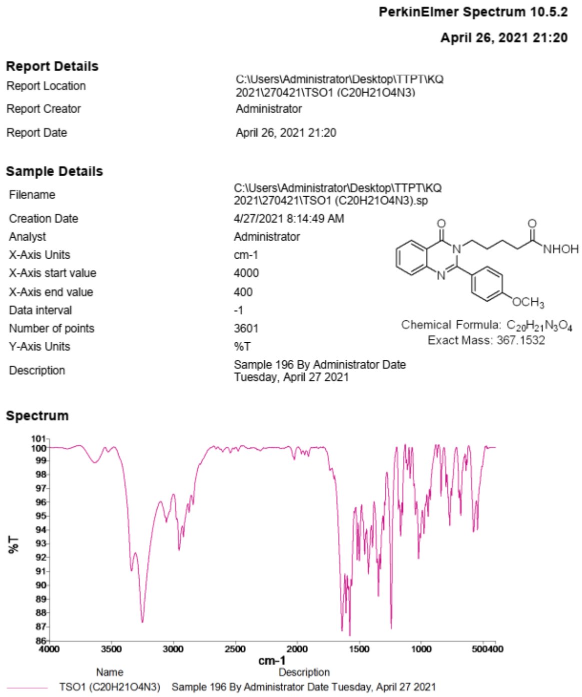

Figure S4.61 IR spectrum of compound **13**

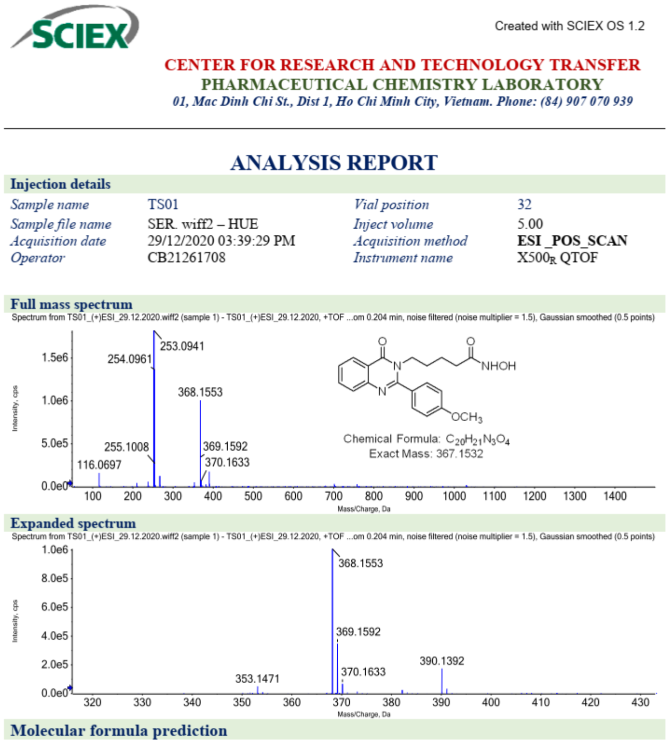

Figure S4.62 MS spectrum of compound **13**

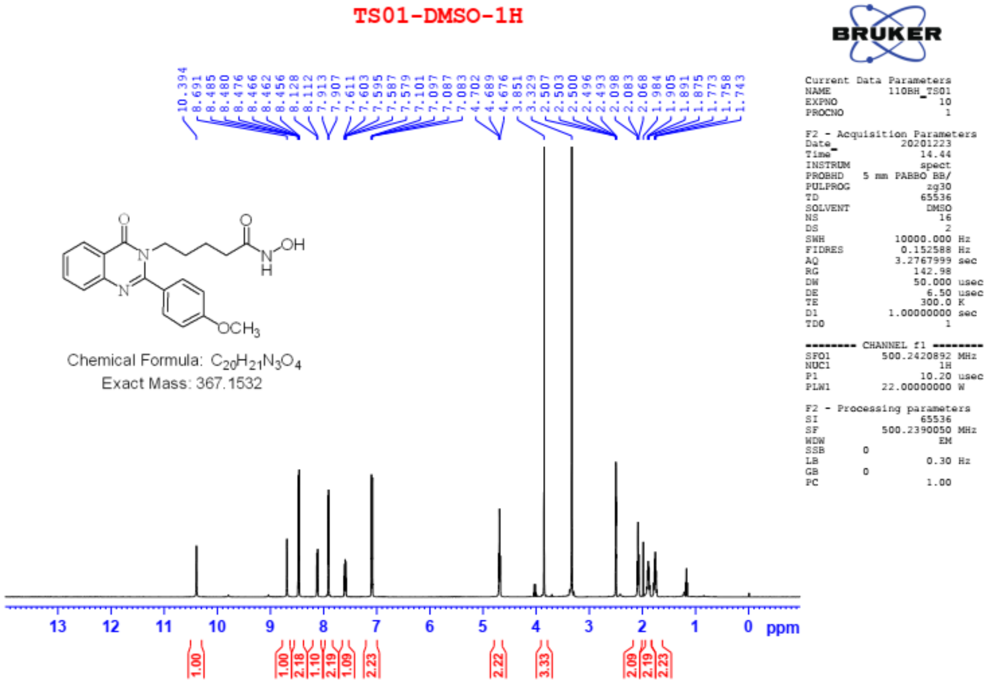

Figure S4.63 ^{1}H-NMR spectrum of compound **13**

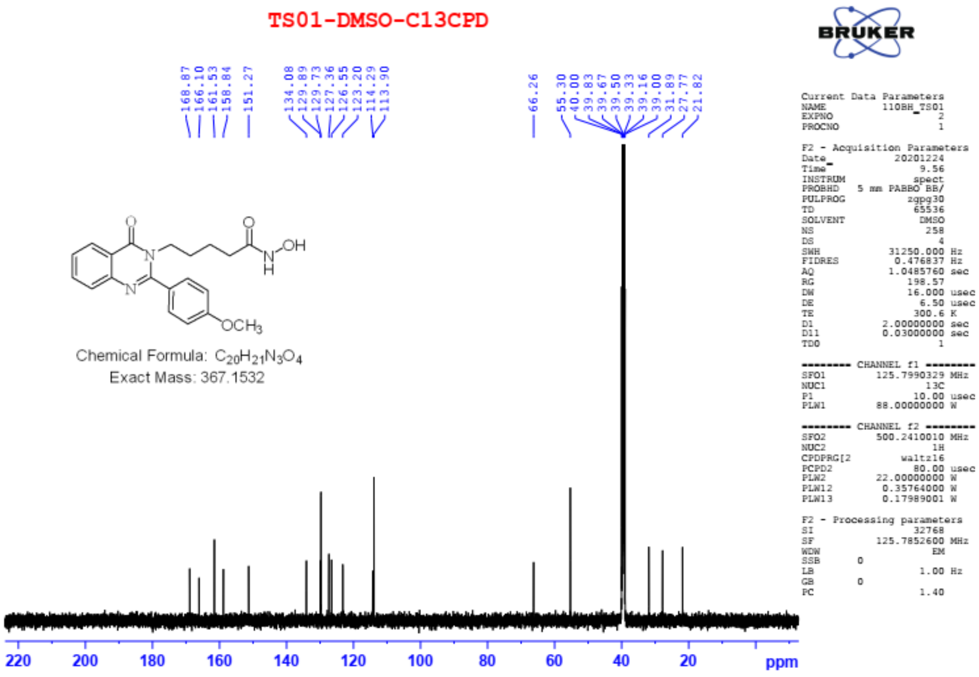

Figure S4.64 ^{13}C-NMR spectrum of compound **13**

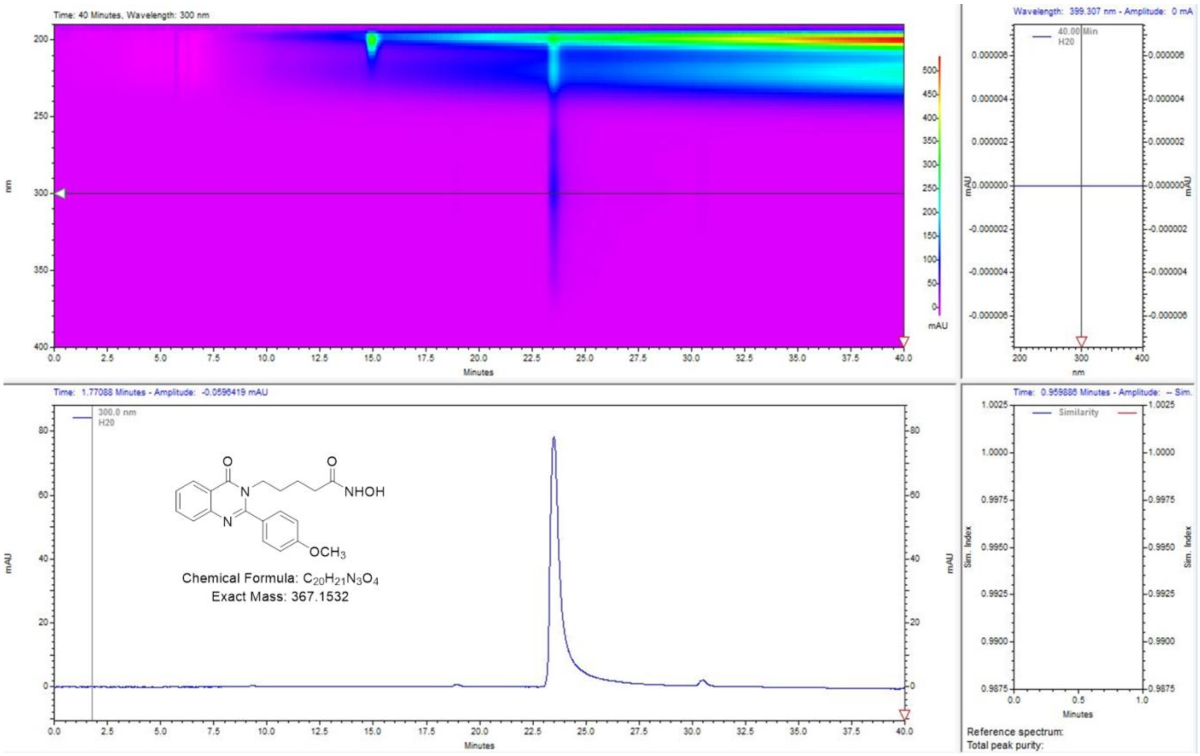

Figure S4.65 HPLC spectrum of compound **13**

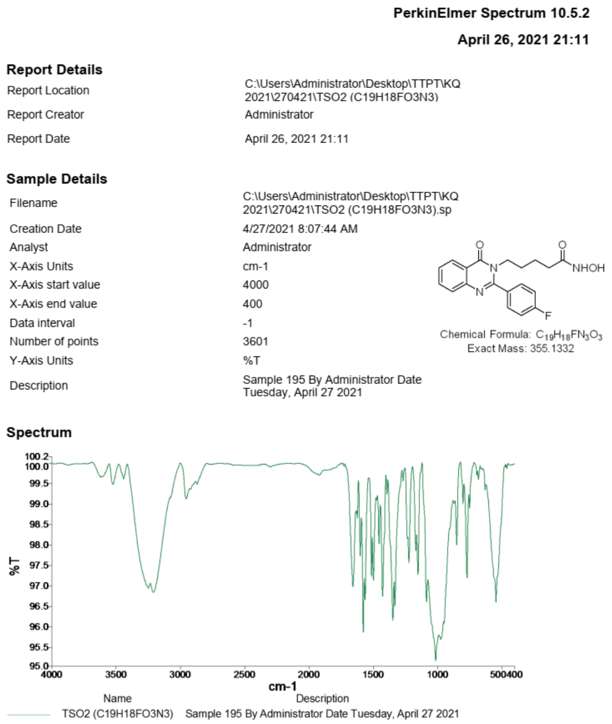

Figure S4.66 IR spectrum of compound **14**

Created with SCIEX OS 1.2

CENTER FOR RESEARCH AND TECHNOLOGY TRANSFER
PHARMACEUTICAL CHEMISTRY LABORATORY
1B, Thanh Loc 29 St., Dist 12, Ho Chi Minh City, Vietnam. Phone: (84) 907 070 939

ANALYSIS REPORT

Injection details

Sample name	TS02	*Vial position*	23
Sample file name	SER. wiff2 – HUE	*Inject volume*	5.00
Acquisition date	19/05/2021 14:23:08 PM	*Acquisition method*	**ESI _POS_SCAN**
Operator	CB21261708	*Instrument name*	X500R QTOF

Full mass spectrum

Spectrum from HUE_TS02_(+)ESI 2021-05-19-14-23-08.wiff2 (sample 1) - HUE_TS02_(+)ESI...m 0.171 min, noise filtered (noise multiplier = 1.5), Gaussian smoothed (0.5 points)

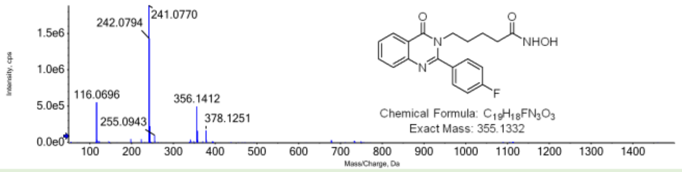

Expanded spectrum

Spectrum from HUE_TS02_(+)ESI 2021-05-19-14-23-08.wiff2 (sample 1) - HUE_TS02_(+)ESI...m 0.171 min, noise filtered (noise multiplier = 1.5), Gaussian smoothed (0.5 points)

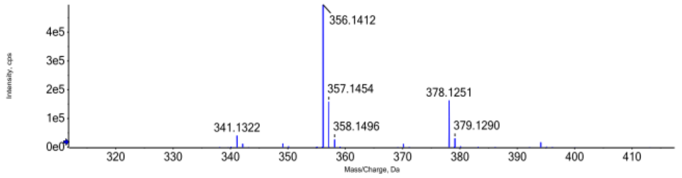

Figure S4.67 MS spectrum of compound **14**

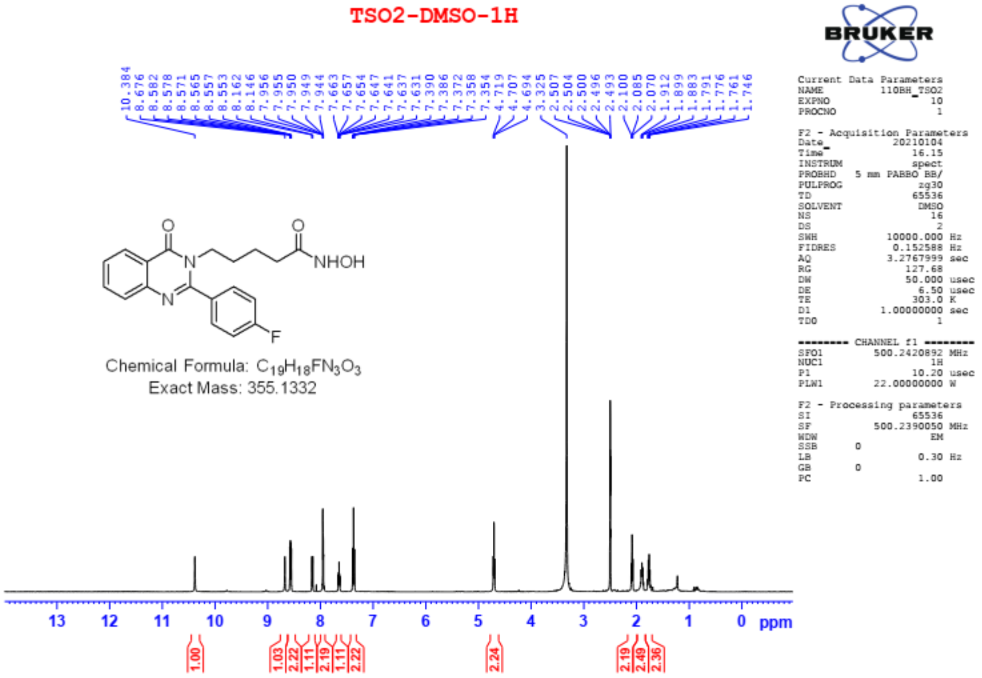

Figure S4.68 ^{1}H-NMR spectrum of compound **14**

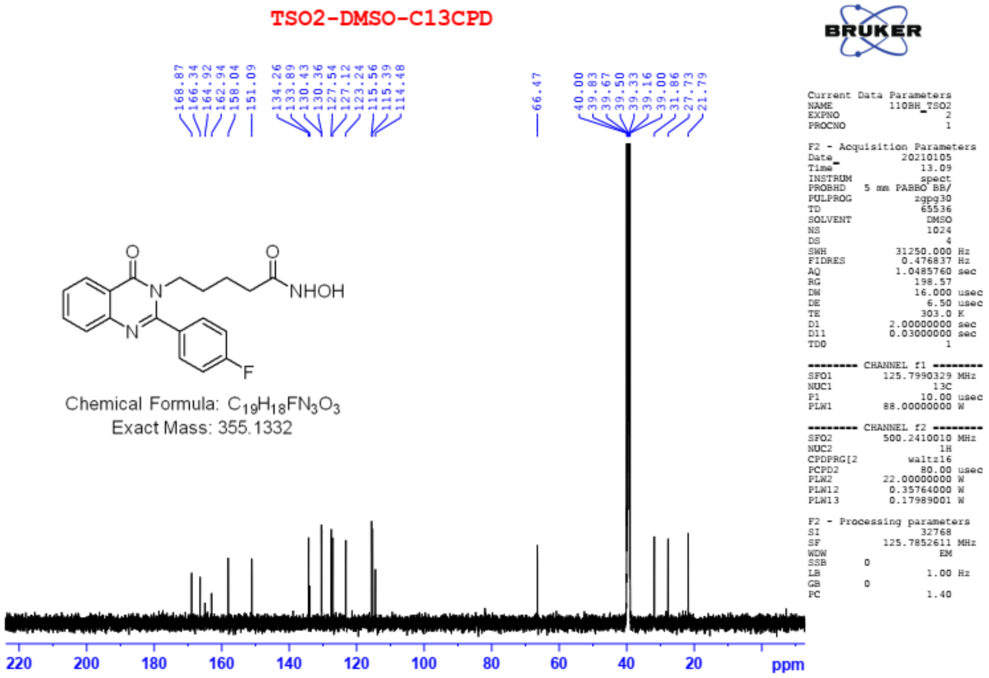

Figure S4.69 ^{13}C-NMR spectrum of compound **14**

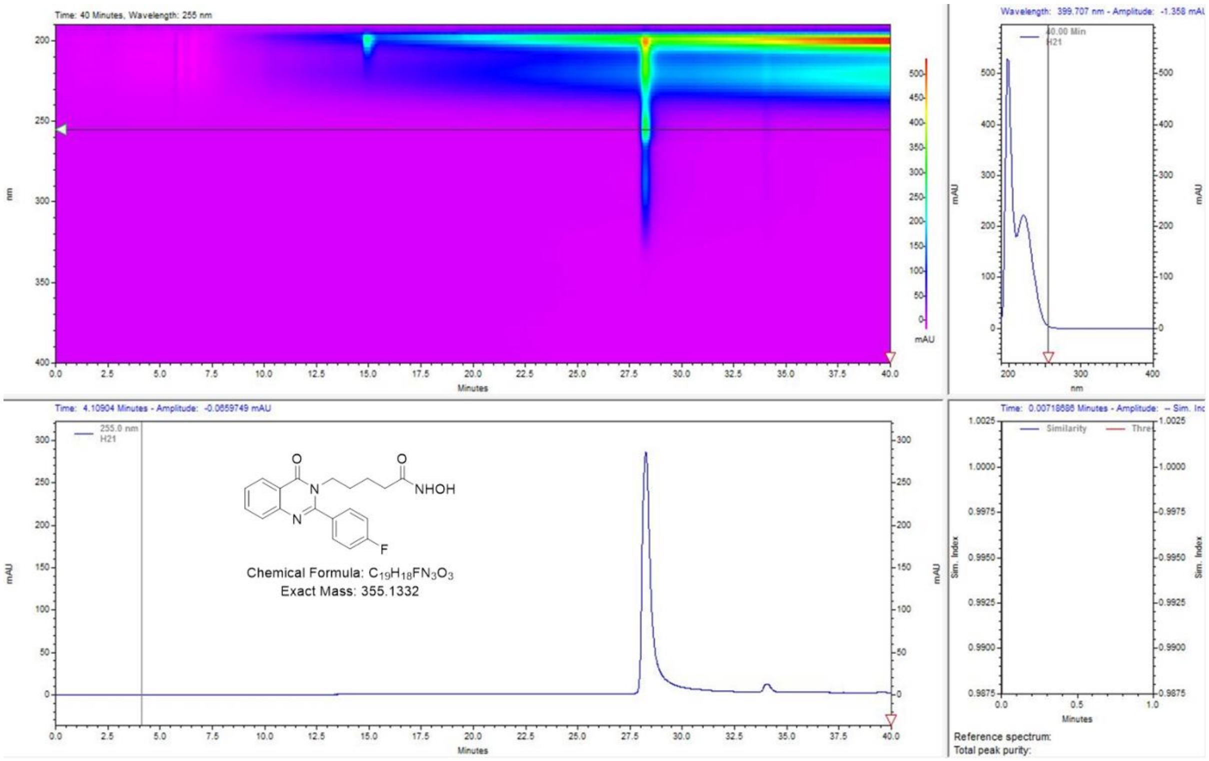

Figure S4.70 HPLC spectrum of compound **14**

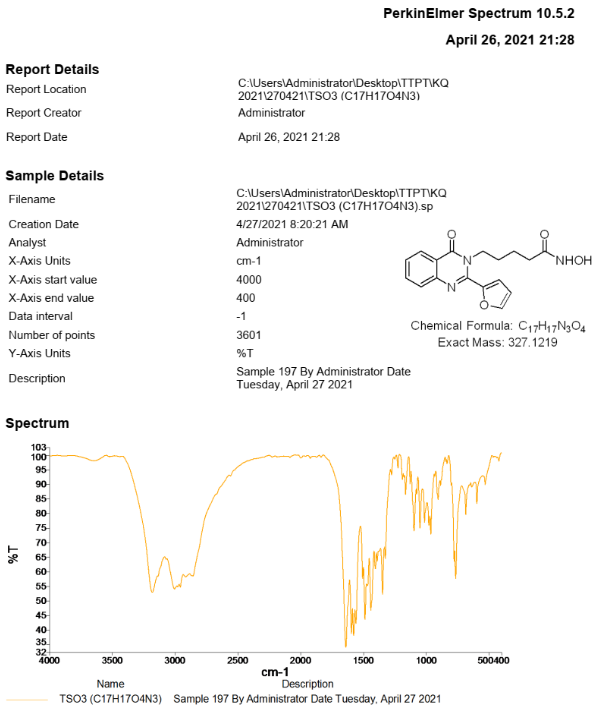

Figure S4.71 IR spectrum of compound **15**

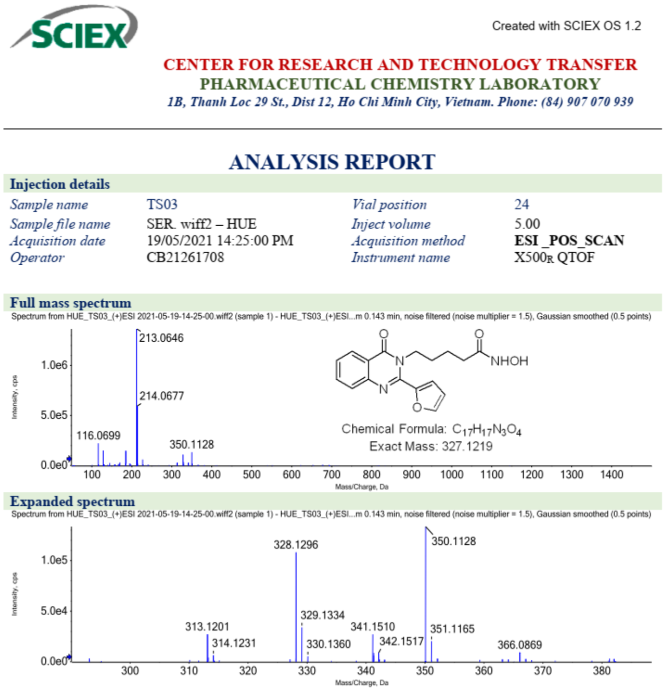

Figure S4.72 MS spectrum of compound **15**

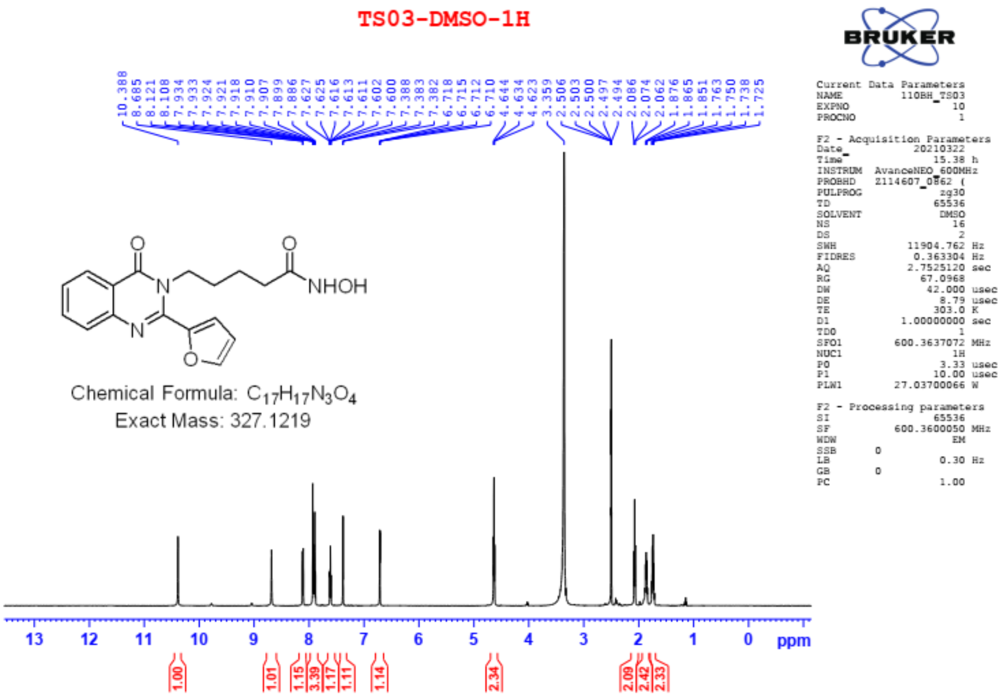

Figure S4.73 ^{1}H-NMR spectrum of compound **15**

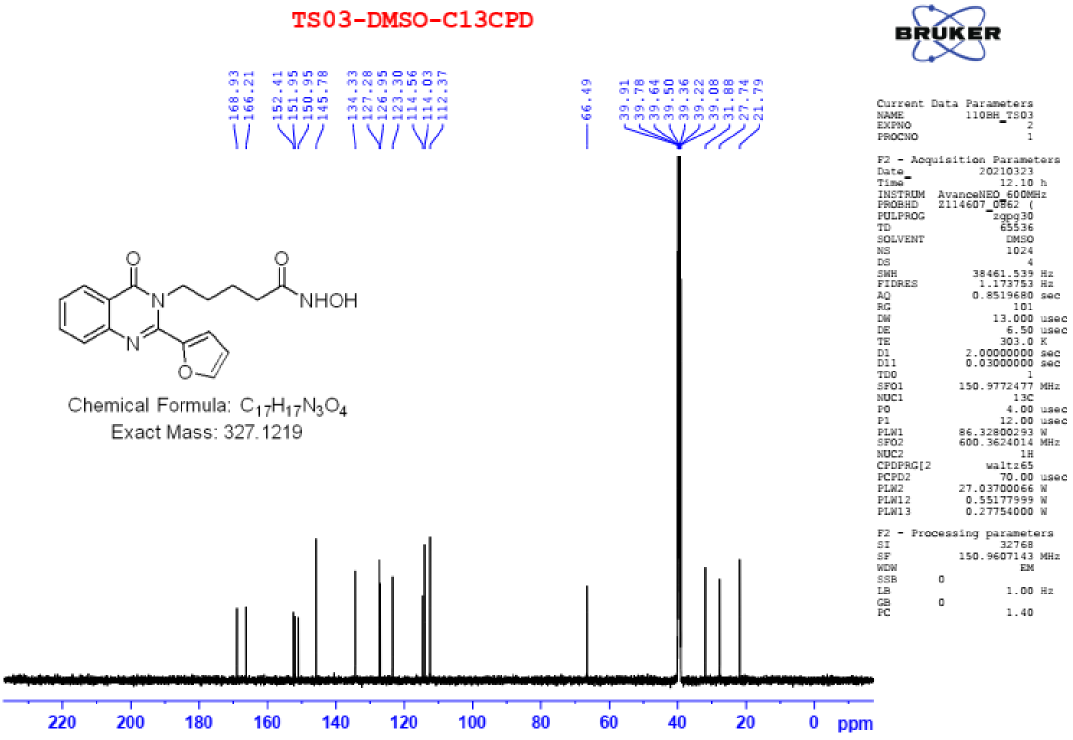

Figure S4.74 ^{13}C-NMR spectrum of compound **15**

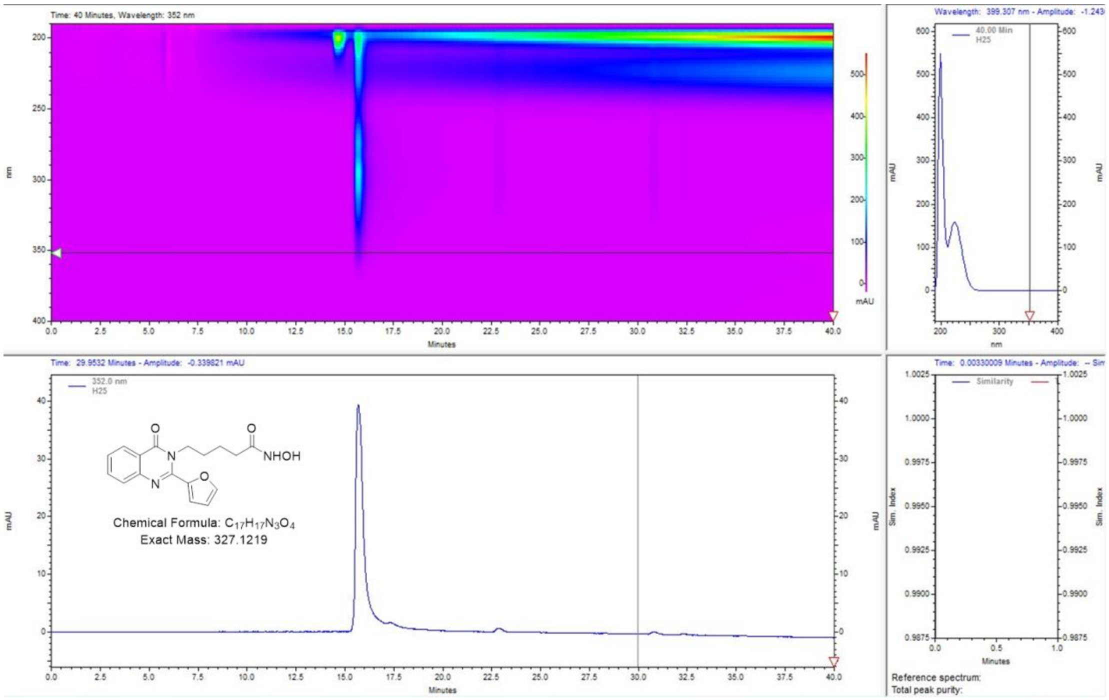

Figure S4.75 HPLC spectrum of compound **15**
